# Normative and mechanistic model of an adaptive circuit for efficient encoding and feature extraction

**DOI:** 10.1073/pnas.2117484120

**Published:** 2023-07-10

**Authors:** Nikolai M. Chapochnikov, Cengiz Pehlevan, Dmitri B. Chklovskii

**Affiliations:** ^a^Center for Computation Neuroscience, Flatiron Institute, New York, NY 10010; ^b^Department of Neurology, New York University School of Medicine, New York, NY 10016; ^c^John A. Paulson School of Engineering and Applied Sciences, Harvard University, Cambridge, MA 02138; ^d^Center for Brain Science, Harvard University, Cambridge, MA 02138; ^e^Kempner Institute for the Study of Natural and Artificial Intelligence, Harvard University, Cambridge, MA 02138; ^f^Neuroscience Institute, New York University School of Medicine, New York, NY 10016

**Keywords:** olfaction, connectome, encoding, clustering, normative approach

## Abstract

One major question in neuroscience is how to relate connectomes to neural activity, circuit function, and learning. We offer an answer in the peripheral olfactory circuit of the *Drosophila* larva, composed of olfactory receptor neurons (ORNs) connected through feedback loops with interconnected inhibitory local neurons (LNs). We combine structural and activity data and, using a holistic normative framework based on similarity-matching, we formulate biologically plausible mechanistic models of the circuit. In particular, we consider a linear circuit model, for which we derive an exact theoretical solution, and a nonnegative circuit model, which we examine through simulations. The latter largely predicts the ORN → LN synaptic weights found in the connectome and demonstrates that they reflect correlations in ORN activity patterns. Furthermore, this model accounts for the relationship between ORN → LN and LN–LN synaptic counts and the emergence of different LN types. Functionally, we propose that LNs encode soft cluster memberships of ORN activity, and partially whiten and normalize the stimulus representations in ORNs through inhibitory feedback. Such a synaptic organization could, in principle, autonomously arise through Hebbian plasticity and would allow the circuit to adapt to different environments in an unsupervised manner. We thus uncover a general and potent circuit motif that can learn and extract significant input features and render stimulus representations more efficient. Finally, our study provides a unified framework for relating structure, activity, function, and learning in neural circuits and supports the conjecture that similarity-matching shapes the transformation of neural representations.

## Significance

The brain represents information with neural activity patterns. At the periphery, these patterns contain correlations, which are detrimental to stimulus discrimination. We study the peripheral olfactory circuit of the *Drosophila* larva, which preprocesses neural representations before relaying them downstream. A comprehensive understanding of this preprocessing is, however, lacking. We formulate a principle-driven framework based on similarity-matching and, using neural input activity, derive a circuit model that largely explains the biological circuit’s synaptic organization. It also predicts that inhibitory neurons cluster odors and facilitate decorrelation and normalization of neural representations. If equipped with Hebbian synaptic plasticity, the circuit model autonomously adapts to different environments. Our work provides a comprehensive approach to deciphering the relationship between structure and function in neural circuits.

Technological advances in connectomics (1, 2) and neural population activity imaging (3) enable the anatomical and physiological characterization of neural circuits at unprecedented scales and detail. However, it remains unclear how to combine these datasets to advance our understanding of brain computation. To address this, we focus on the peripheral olfactory system of the first instar *Drosophila* larva—a small and genetically tractable circuit with available connectivity and activity imaging datasets (4, 5).

**Fig. 1.**
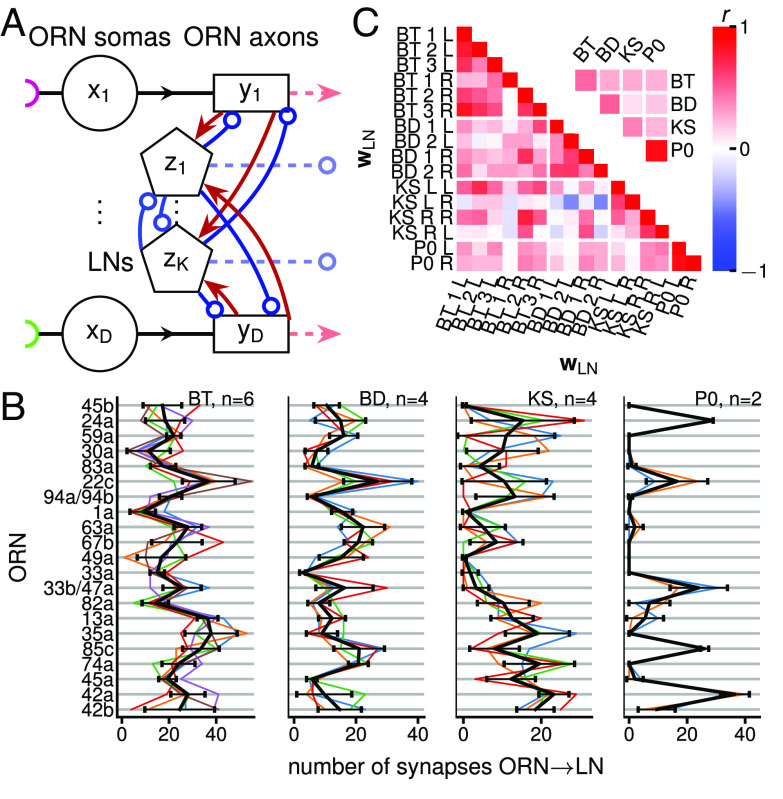
Circuit connectivity and LN types. (*A*) ORN-LN circuit diagram. $x_{i}$, $y_{i}$, $z_{i}$: activity each ORN soma (circle), axonal terminal (rectangle), and LN (pentagon). Each ORN is depicted as a two-compartment unit with a soma and an axon. Half-circles: different types of chemical receptors. Red lines with arrowheads, blue lines with open circles: excitatory and inhibitory connections. LNs reciprocally connect with ORN axons and between themselves. ORN axons and LNs synapse onto neurons downstream (dashed lines). (*B*) Feedforward ORNs → LN synaptic count vectors, $w_{\text{LN}}$ (colored lines), and average feedforward ORNs → LN${}_{\text{type}}$ synaptic count vectors, $w_{\text{LNtype}}$ (black lines, mean ± SD) for each LN type (*SI Appendix*, Fig. S2*A*). (*C*) Correlation coefficients *r* between all $w_{\text{LN}}$. L, R: left and right side of the Drosophila larva. The numerical indices of BT and BD are arbitrary, and there is no correspondence between the left and right side indices. Although BT 1 R is of the same type as other BT, its connection vector has a correlation of 0 with other BT in the connectome data. Inset: Mean rectified correlation coefficient ${\bar{r}}_{+}$ ($r_{+}:=\max\left[0,r\right]$, i.e., negative values are set to 0) between LN types calculated by averaging the rectified values in each region delimited by a white border, excluding the diagonal entries of the full matrix.

This circuit is an analogous but simpler version of the well-studied olfactory circuit in adult flies and vertebrates (6). It contains 21 olfactory receptor neurons (ORNs), each expressing a different receptor type (Fig. 1*A*). ORN axons are reciprocally connected to a web of multiple interconnected inhibitory local neurons (LNs) through feedforward excitation and feedback inhibition. The connectome dataset contains not only the presence or absence of a connection between two neurons, but also the number of synaptic contacts in parallel (4), which is an estimate of the connection strength (2, 7–9) (nonetheless, other factors like release probability and active zone properties also affect synaptic strength (10, 11)).

Previous studies examined the role of LNs in transforming the neural representation of odors from ORN somas to downstream projection neurons (PNs). In adult *Drosophila*, this circuit was suggested to perform gain control and divisive normalization (12, 13), which equalizes different odor concentrations and decorrelates input channels. In the zebrafish larva, an analogous circuit was suggested to whiten the input, leading to pattern decorrelation, which helps odor discrimination downstream (14, 15).

However, the underlying mechanistic principles of computation remain elusive. For example, while different types of LNs have different connectivity patterns with ORNs in the *Drosophila* larva (4), the role of different LN types, their multiplicity, and their specific connectivity is not yet understood. Furthermore, the peripheral olfactory circuit of adult *Drosophila* exhibits synaptic plasticity in response to changes in the olfactory environment (16–19), but the functional role of this plasticity is unclear.

**Fig. 2.**
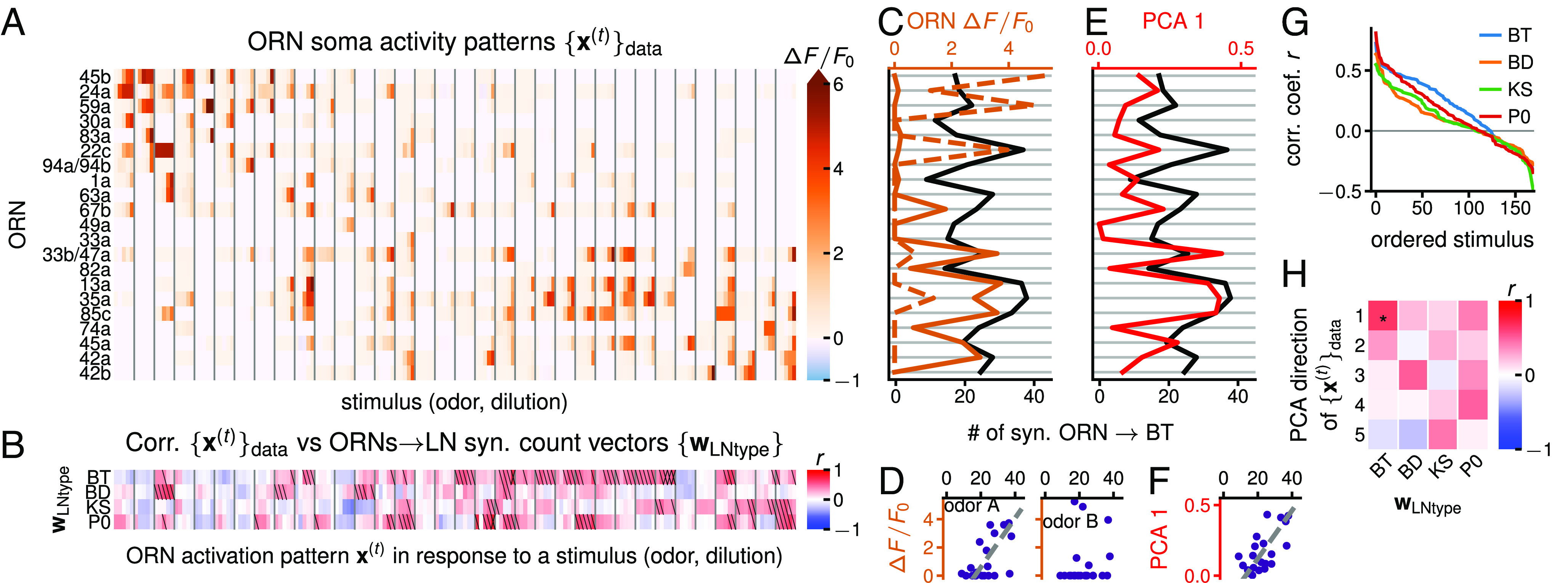
Alignment of ORNs → LN synaptic count vectors with odor representations in ORN activity. (*A*) Ca^2+^$\Delta F/F_{0}$ activity patterns ${\left\{x^{\left(t\right)}\right\}}_{\text{data}}$ in ORN somas in response to 34 odors (separated by vertical gray lines) at 5 dilutions ($10^{-8},...,10^{-4}$) from ref. 5. See *SI Appendix*, Fig. S3 for odor labels and scaled ${\left\{x^{\left(t\right)}\right\}}_{\text{data}}$. (*B*) Correlation between the four ORNs → LN${}_{\text{type}}$ synaptic count vectors ($w_{\text{LNtype}}$ for BT, BD, KS, and P0) with the odor representations ${\left\{x^{\left(t\right)}\right\}}_{\text{data}}$ from (*A*). Slash: significant at 0.05 level; cross: significant at 0.05 FDR (false discovery rate) (31). *P*-values calculated by shuffling the entries of each $w_{\text{LNtype}}$ (50,000 permutations). (*SI Appendix*, Figs. S4*A* and S5). (*C*) ORNs → Broad Trio synaptic count vector $w_{\text{BT}}$ superimposed with ORN activity patterns $x^{\text{(A)}}$ and $x^{\text{(B)}}$ in response to the ligands 2-heptanone (odor A) and 2-acetylpyridine (odor B) at dilution $10^{-4}$. y-axis: ORN, follows order of (*A*). (*D*) Scatter plot representation of (*C*). $w_{\text{BT}}$ is more strongly tuned to $x^{\text{(A)}}$ (r=0.6, P=0.004) than to $x^{\text{(B)}}$ (r=0.14, P=0.3). *P*-values not adjusted for multiple comparisons. (*E*) $w_{\text{BT}}$ superimposed on the 1^st^ PCA direction of ${\left\{x^{\left(t\right)}\right\}}_{\text{data}}$. y-axis: ORN, follows order of (*A*). (*F*) Scatter plot representation of (*E*) (r=0.65, P=0.001). *P*-values are not adjusted for multiple comparisons. (*G*) LN “connectivity tuning curves”: correlation coefficients sorted in decreasing order from (*B*) for each $w_{\text{LNtype}}$. (*H*) Correlation coefficient *r* between the top 5 PCA directions of ${\left\{x^{\left(t\right)}\right\}}_{\text{data}}$ and the four $w_{\text{LNtype}}$s (*SI Appendix*, Fig. S6 *A*, *B*, and *E*). Two-sided *P*-values calculated by shuffling the entries of each $w_{\text{LNtype}}$ (50,000 permutations). *: significance at 0.05 FDR.

**Fig. 3.**
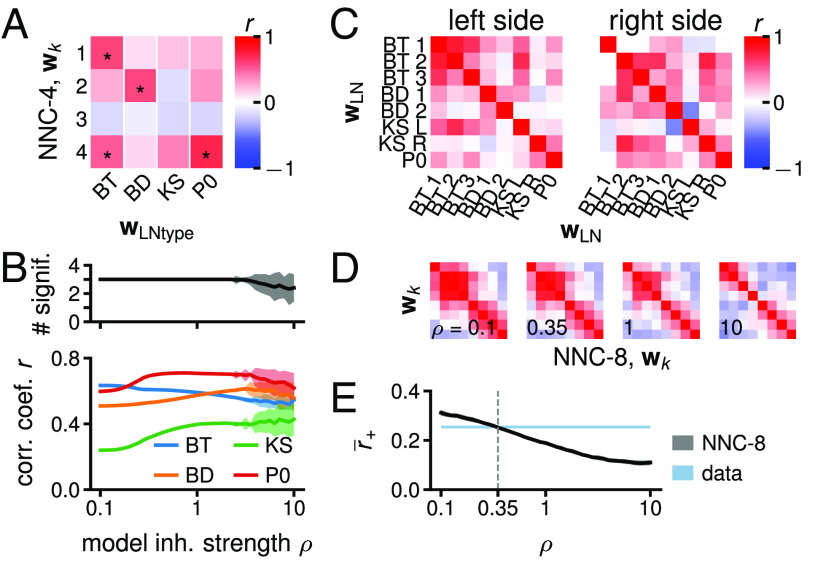
Prediction of the connectivity with the NNC and emergence of LN groups. (*A*) Correlation between the four ORNs → LN connection weight vectors $\left\{w_{k}\right\}$ from NNC-4 (ρ=1) and the four ORNs → LN_type_ synaptic count vectors $\left\{w_{\text{LNtype}}\right\}$ (*SI Appendix*, Fig. S6 *C*, *D*, *F*, *G*, and *H*). One-sided *P*-values calculated by shuffling the entries of each $w_{\text{LNtype}}$ (50,000 permutations). *: significant at 0.05 FDR. (*B*) *Bottom*: maximum correlation coefficient (mean ± SD) of the four $w_{k}$s from NNC-4 with the four $w_{\text{LNtype}}$s for different values of ρ (50 simulations per ρ), encoding the feedback inhibition strength. *Top*: number of $w_{\text{LNtype}}$s significantly correlated with at last one $w_{k}$ from NNC-4 (FDR at 5%). For ρ⪆3.1, not all simulations converge to the same $\left\{y^{\left(t\right)}\right\}$, $\left\{z^{\left(t\right)}\right\}$, and $\left\{w_{k}\right\}$, potentially due to existence of multiple global optima or simulations only finding local optima. (*C*) Correlation between the $w_{\text{LN}}$s on the left and right sides of the larva, portraying that several $w_{\text{LN}}$s are similar. (*D*) Same as (*C*) for the eight $w_{k}$s arising from NNC-8 and with ρ=0.1,0.35,1,10. Matrices ordered using hierarchical clustering and $w_{k}$s ordered accordingly (*SI Appendix*). (*E*) Mean rectified correlation coefficient ${\bar{r}}_{+}$ ($r_{+}:=\max\left[0,r\right]$) from (*C*) (blue band delimited by the value for left and right circuit) and from NNC-8 (black line, mean ± SD, 50 simulations per ρ). ${\bar{r}}_{+}$ obtained by averaging all the $r_{+}$ from a correlation matrix, i.e., (*C*) or (*D*), excluding the diagonal.

**Fig. 4.**
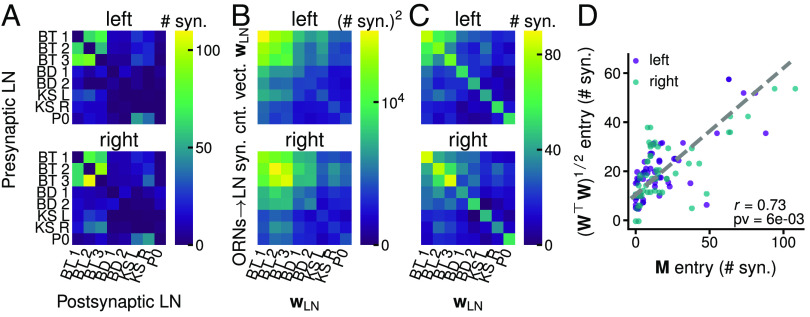
Relationship between LN–LN (M) and ORNs → LN (W) synaptic counts in the connectome reconstruction. (*A*) LN–LN connections synaptic counts M on the left and right sides of the larva. (*B*) $W^{\text{T}}W$ with $W=[w_{\text{LN1},...,}w_{\text{LN8}}]$ on the left and right sides. Thus each entry is $w_{\text{LNi}}^{\text{T}}w_{\text{LNj}}$, the scalar product between 2 ORNs → LN synaptic count vectors $w_{\text{LN}}$. (*C*) ${\left(W^{\text{T}}W\right)}^{1/2}$, i.e., the square root of the matrices in (*B*). (*D*) Entries of M vs entries of ${\left(W^{\text{T}}W\right)}^{1/2}$, excluding the diagonal, for both sides. *r*: Pearson correlation coefficient. pv: one-sided *P*-value calculated by shuffling the entries of each $w_{\text{LN}}$ independently, which assures that each LN keeps the same total number of synapses. Shuffling the entries of M in addition to shuffling each $w_{\text{LN}}$ leads to *P*-value $<10^{-4}$.

**Fig. 5.**
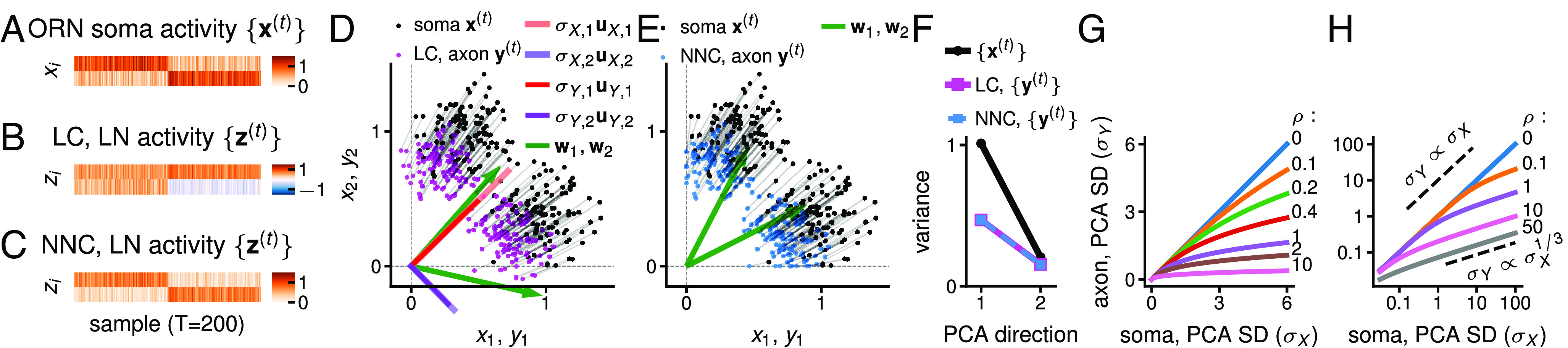
Computation in the LC and NNC. (*A*) Artificial ORN soma activity patterns ($\left\{x^{\left(t\right)}\right\}$, D=2 ORN somas), generated with two Gaussian clusters of 100 points each centered at (1, 0.3) and (0.3, 1), =0.17. This input is fed to the LC-2 (i.e., K=2 LNs) (*B*, *D*, and *F*) and the NNC-2 (*C*, *E*, and *F*), ρ=1. (*B*) LN activity, $\left\{z^{\left(t\right)}\right\}$, in the LC-2. Because of a degree of freedom in LC, LN activity can be any rotation of the activity depicted here, i.e., Q·z, where Q is a rotation (orthogonal) matrix. (*C*) LN activity, $\left\{z^{\left(t\right)}\right\}$, in the NNC-2. LNs encode cluster memberships. (*D*) Scatter plot of the activity patterns in ORN somas ($\left\{x^{\left(t\right)}\right\}$, black, from (*A*) and in ORN axons in the LC-2 ($\left\{y^{\left(t\right)}\right\}$, magenta). $\sigma_{X,i}u_{X,i}$, $\sigma_{Y,i}u_{Y,i}$: vectors of the PCA directions of uncentered $\left\{x^{\left(t\right)}\right\}$ and $\left\{y^{\left(t\right)}\right\}$ scaled by the SD of that direction. $w_{k}$ (green): direction of an ORNs → LN synaptic weight vector in the LC-2 from (*B*). Rotating the LN output $\left\{z^{\left(t\right)}\right\}$ would alter the $w_{k}$s, but not the $\left\{y^{\left(t\right)}\right\}$. (*E*) Scatter plot of the activity patterns in ORN somas ($\left\{x^{\left(t\right)}\right\}$, black, from (*A*) and in ORN axons in the NNC-2 ($\left\{y^{\left(t\right)}\right\}$, blue). All activities are nonnegative and the $w_{k}$s point toward the cluster locations, enabling the clustering observed in (*C*). (*F*) The PCA variances of the activity are less dispersed in ORN axons (output, $\left\{y^{\left(t\right)}\right\}$) than in ORN somas (input, $\left\{x^{\left(t\right)}\right\}$) for the LC and NNC. The output representation is thus partially whitened. The LC and NNC are similar in terms of their PCA variances. (*G* and *H*) Transformation of the SD ($\sigma_{X}$, $\sigma_{Y}$) of PCA directions from ORN somas ($\left\{x^{\left(t\right)}\right\}$) to ORN axons ($\left\{y^{\left(t\right)}\right\}$) in the LC model on linear and logarithmic scales, for different values of ρ (different line colors), encoding inhibition strength. When ρ=0, the output equals the input. The higher the ρ, the smaller the PCA variances in the ORN axon.

**Fig. 6.**
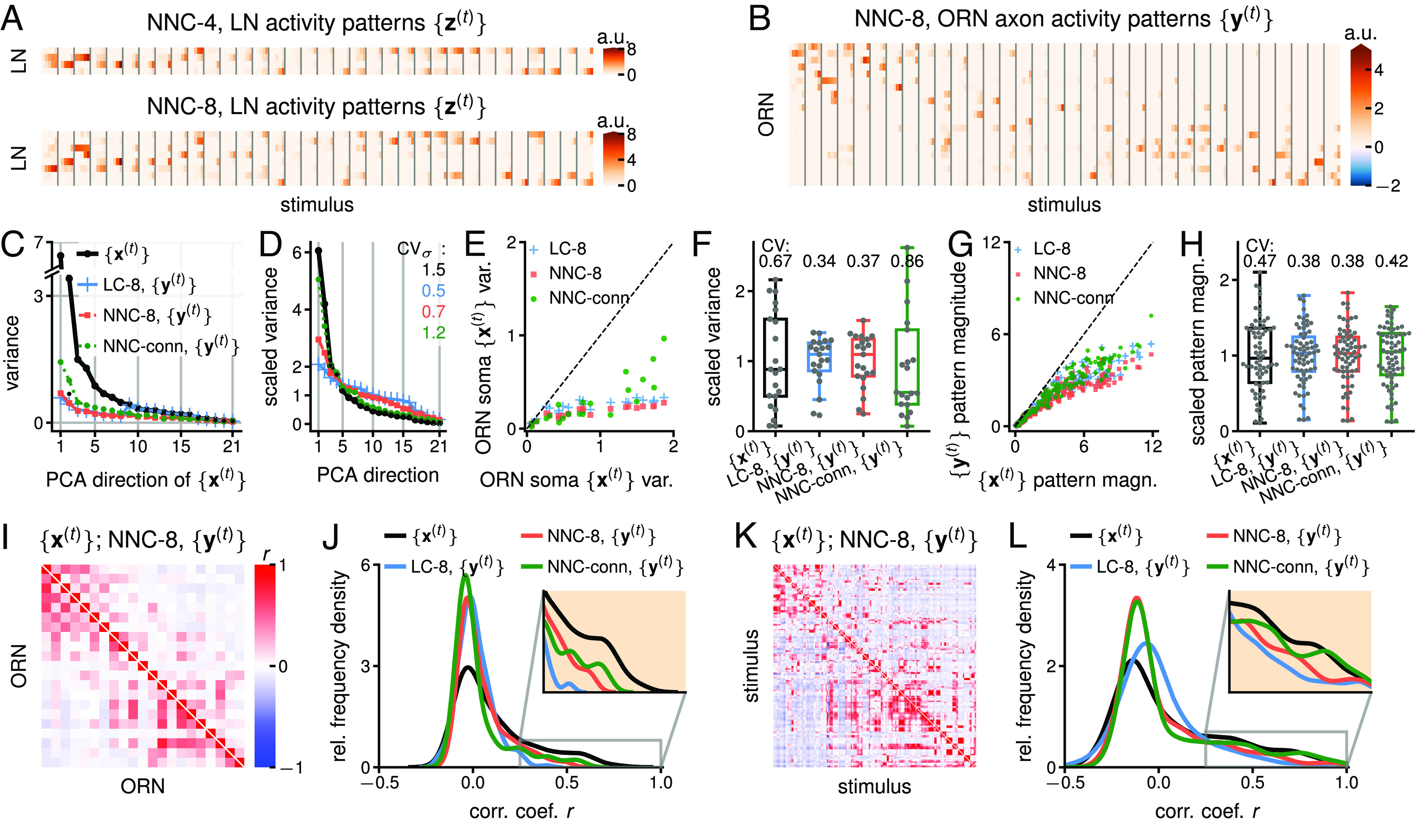
Computation in the LC, NNC, and NNC-conn models in response to ${\left\{x^{\left(t\right)}\right\}}_{\text{data}}$ (Fig. 2*A*): clustering, partial whitening, normalization, and decorrelation. (*A*) LN activity, $\left\{z^{\left(t\right)}\right\}$, for the NNC-4 and NNC-8 models (*SI Appendix*, Fig. S11). LNs are mostly active in response to the odors to which their connectivity is the most aligned (*SI Appendix*, Fig. S8*A*). (*B*) ORN axon activity, $\left\{y^{\left(t\right)}\right\}$, in the NNC-8. (*C*) Variances of odor representations in ORN somas (${\left\{x^{\left(t\right)}\right\}}_{\text{data}}$) and axons ($\left\{y^{\left(t\right)}\right\}$) in the PCA directions of uncentered (${\left\{x^{\left(t\right)}\right\}}_{\text{data}}$). The variances decrease the strongest in the directions of the highest initial variance. (*D*) Uncentered PCA variances ${\left\{x^{\left(t\right)}\right\}}_{\text{data}}$ and $\left\{y^{\left(t\right)}\right\}$ scaled by their mean to portray the spread of variances. (*E*) Uncentered variances of activity at ORN axons ($\left\{y^{\left(t\right)}\right\}$, output) vs. in ORN somas (${\left\{x^{\left(t\right)}\right\}}_{\text{data}}$, input). (*F*) Box plot of the ORN activity variances from (*E*) scaled by their mean to show the spread of variances. (*G*) Magnitude of the 170 activity patterns in ORN axons $\left\{y^{\left(t\right)}\right\}$ vs in somas ${\left\{x^{\left(t\right)}\right\}}_{\text{data}}$. (*H*) Box plot of the activity pattern magnitudes from (*G*) (only for top two dilutions $10^{-5}$ and $10^{-4}$) scaled by their mean to show the spread of magnitudes. (*I*) Correlations between the activity of ORN somas (${\left\{x^{\left(t\right)}\right\}}_{\text{data}}$, *Lower Left* triangle) and of ORN axon activity in NNC-8 ($\left\{y^{\left(t\right)}\right\}$, *Upper Right* triangle). (*J*) Smoothed histogram of the channel correlation coefficients from (*J*), excluding the diagonal (based on n=210 values). In all models, at the axonal level, there are more correlation coefficients around zero and fewer at higher values. (*K*) Correlations between activity patterns (i.e., odor representations) in ORN somas (${\left\{x^{\left(t\right)}\right\}}_{\text{data}}$, *Lower Left* triangle) and in ORN axons for NNC-8 ($\left\{y^{\left(t\right)}\right\}$, *Upper Right* triangle). (*L*) Smoothed histogram of the activity pattern correlation coefficients from (*K*) (only for top two dilutions $10^{-5}$ and $10^{-4}$, n = 2,278). Similar effect as for channels in (*J*). The decorrelation in the LC is more effective than in the NNC. The decorrelation in NNC-conn is not as pronounced as for the other two models. ρ=2 in this figure. a.u.: arbitrary units, stands for appropriate unit of neural activity. See *SI Appendix*, Figs. S12–S16 for the alignment of PCA direction, the LC, the NNC, the NNC-conn, and ρ=10.

To address these shortcomings, we use a combination of data analysis and modeling and develop a holistic theoretical framework that links circuit structure, function, activity data, and learning. Our contribution is fivefold: 1) We find that the vectors of the number of synapses between ORNs and LNs reflect features of the independently acquired ORN activity pattern dataset (Figs. 2 and 3). 2) Building upon the normative similarity-matching framework (20, 21), we develop an optimization problem solvable by a biologically realistic circuit model with the same architecture as the ORN-LN circuit. 3) The model, driven by the ORN activity dataset, largely predicts the following observations in the structural dataset (Figs. 3 and 4): the ORNs → LN synaptic weights, the emergence of LN groups, and the relationship between feedforward ORN → LN and lateral LN–LN connections. 4) Using our model, we characterize the circuit computation (Figs. 5 and 6), and propose that LNs play a dual role in rendering the neural representation of odors in ORNs more efficient and extracting useful features that are transmitted downstream. 5) We show that the synaptic weights that enable this computation can, in principle, be learned in an unsupervised manner via Hebbian plasticity. Note that, given the connectome (4) originates from a 6-h-old first instar *Drosophila* larva, new synaptic contact formation can take longer than 6 h (11), and no study has yet demonstrated activity-dependent plasticity in the larval ORN-LN circuit, it is unknown whether the observed synaptic counts in this connectome could result from activity-dependent synaptic plasticity.

In this study, we further our understanding of LNs and their computations. We highlight the importance of minutely organized ORN–LN and LN–LN connection weights, which allow LNs to encode different significant features of input activity and dampen them in ORN axons. The transformation from the representation in ORN somas to that in ORN axons consists of a partial equalization of PCA variances, which enables a more efficient stimulus encoding (22). In fact, this results in a decorrelation and equalization of ORNs and odor representations, which correspond to two fundamental computations in the brain: partial ZCA (zero-phase) whitening (23, 24) and divisive normalization (25). In essence, we uncover an elegant neural circuit motif that can extract features and perform two critical computations. If endowed with Hebbian plasticity, the circuit can also adapt and perform its functions in different stimulus environments. Thus, we present a framework that allows us to quantitatively link synaptic weights in the structural data with the circuit’s function and with the circuit adaptation to input correlations, thus making a crucial step toward a more integrated understanding of neural circuits.

The results are organized as follows. First, we show that the connectome is adapted to ORN activity patterns. Second, we propose a normative approach leading to two circuit models: a linear circuit (LC) model, and a nonnegative circuit (NNC) model. Third, we show that the NNC reproduces key structural observations. Finally, we describe the computations performed by the LC and NNC in general and on the ORN activity dataset in particular.

## Results

### ORN-LN Circuit.

ORNs in the *Drosophila* larva carry odor information from the antennas to the antennal lobe, where they synapse onto LNs and PNs. There, olfactory information is reformatted and transferred through ORN axons and LNs to PNs. LNs, which synapse bidirectionally with ORN axons and PN dendrites, strongly contribute to the reformatting in ORNs and PNs through presynaptic and postsynaptic inhibition, as shown mainly in the adult fly (12, 13, 26–30). LNs project to several uni- and multiglomerular PNs, and PNs project to higher brain areas such as the mushroom body and the lateral horn (4).

We study the circuit and computation presynaptic to PNs, i.e., occurring from ORN somas to ORN axons and LNs. Specifically, we examine the subcircuit formed by all D=21 ORNs and those 4 LN types (on each side of the brain) that reciprocally connect with ORNs (4) (Fig. 1*A*, *SI Appendix*, Fig. S1). The 4 LN types include 3 Broad Trio (BT) neurons, 2 Broad Duet (BD) neurons, 1 Keystone (KS, bilateral connections) neuron, and 1 Picky 0 (P0) neuron (*SI Appendix*, Figs. S1 and S2*A*). This amounts to 8 ORNs–LN connections per side (3 BTs, 2 BDs, 2 KSs, and 1 P0s) and 16 on both sides. See *SI Appendix*, Tables S1 and S2 for a list of all acronyms and mathematical variables used in the paper.

We use the number of synaptic contacts in parallel between two neurons as a proxy for the synaptic weight (2, 7–9) (but see refs. 10 and 11). In the linear approximation, the change in the postsynaptic neuron activity due to a change in the presynaptic neuron activity is proportional to the synaptic weight connecting them.

We focus our analysis on the synaptic counts of the feedforward ORNs → LN connections. We call $w_{\text{LN}}$ the D=21 dimensional vector containing the synaptic counts of the connections from the 21 ORNs to one LN. Because all the entries of this synaptic count vector $w_{\text{LN}}$ share the same postsynaptic neuron, this vector is likely proportional to the corresponding synaptic weight vector. Conversely, the synaptic count vector from one LN to all 21 ORNs may not be proportional to the corresponding synaptic weight vector, because each connection affects a different postsynaptic ORN, which potentially has different electrical properties. This makes the entries of a feedback synaptic count vector not directly comparable. Yet, the feedforward and feedback synaptic count vectors are somewhat correlated (*SI Appendix*, Fig. S2).

While the study (4) divided LNs into the above types based on their neuronal lineage, morphology, and qualitative connectivity, we also find that these types are innervated differently by ORNs (Fig. 1*B*). Indeed, the average correlation of $w_{\text{LN}}$s within each LN type is higher than between LN types (Fig. 1*C*). Thus, for a part of our study (Figs. 2 and 3 *A* and *B*) we use the 4 average $w_{\text{LNtype}}=\frac{1}{n}\sum_{\text{LN}\in \text{LNtype}}w_{\text{LN}}$, where *n* is the number of connection vectors for that LN type.

### ORNs → LN Synaptic Count Vectors Are Adapted to Odor Representations in ORNs.

Several studies proposed that LNs could facilitate the decorrelation of the neural representation of odors (14, 15, 32–35). To perform such decorrelation, the circuit must be adapted to or “know about” the correlations in the activity patterns (36). We investigate whether this is the case in this olfactory circuit by testing whether the $w_{\text{LNtype}}$s contain signatures of ORN activity patterns.

An ensemble of ORN activity patterns ${\left\{x^{\left(t\right)}\right\}}_{\text{data}}$ (t=1,...,170) was obtained using Ca^2+^ fluorescence imaging of ORN somas in response to a set of 34 odorants at 5 dilutions (5) (Fig. 2*A* and *SI Appendix*). These odorants were chosen from the components of fruits and plant leaves from the larva’s natural environment to stimulate ORNs as broadly and evenly as possible, with many odorants activating just a single ORN at the lowest concentration (i.e., the highest dilution).

We examine the Pearson correlation coefficients between the activity patterns ${\left\{x^{\left(t\right)}\right\}}_{\text{data}}$ and the ORNs → LN${}_{\text{type}}$ synaptic count vectors $\left\{w_{\text{LNtype}}\right\}$ (Fig. 2 *C* and *D* for $w_{\text{BT}}$ and two odors; Fig. 2*B* for all four $w_{\text{LNtype}}$s and all activity patterns ${\left\{x^{\left(t\right)}\right\}}_{\text{data}}$). After controlling for multiple comparisons (31), we find that the $w_{\text{LNtype}}$s for the Broad Trio and Picky 0 maintain significant correlations (P<0.05) with a selection of ORN activity patterns, BT being highly correlated with the largest set of $x^{\left(t\right)}$s. This suggests that the synaptic count vectors of at least these two LN types are more adapted to these activity patterns than would be expected by chance (see *SI Appendix*, Fig. S4 and *SI Appendix* for additional evidence). This supports the hypothesis that the circuit is at least partially adapted to ORN activity patterns and that it could perform a computation like decorrelation of input stimuli.

Each $w_{\text{LNtype}}$ exhibits a different “connectivity tuning curve” shape (Fig. 2*G*), $w_{\text{BT}}$ being correlated with the largest set of $x^{\left(t\right)}$s, and $w_{\text{P0}}$ the most highly correlated to a few $x^{\left(t\right)}$s, and the $w_{\text{BD}}$ and $w_{\text{KS}}$ the most weakly correlated. Biologically, this could signify that the BT type is activated by the largest set of odors and P0 only by a few odors. One possibility is that a different set of odors activates each LN class.

### A Normative and Mechanistic Model of the ORN-LN Circuit.

We aim to understand the circuit’s computation and organization using a top-down, normative (also called principle-driven) approach, which involves formulating an optimization problem. Such an approach provides us with a theoretical understanding of the computation and organizational principles of the circuit. Although a bottom-up modeling approach requires unavailable physiological circuit parameters, we verified our predictions with a connectome-constrained model (Fig. 6).

Previous studies suggest that analogous circuits perform stimulus whitening or decorrelation (14, 15, 32–35), and our analysis above supports the possibility of such a computation. A class of optimization problems based on the similarity-matching principle and solvable by circuits similar to the ORN-LN one has been shown to be capable of implementing whitening, principal subspace extraction, and clustering (20, 21, 37). Note that the circuit’s synaptic weights are adapted (optimized) to the ensemble of inputs to perform such computation.

To understand the circuit, we first postulate an optimization problem (Eq. **4**) based on the similarity-matching principle and solvable by a circuit with the ORN-LN architecture (see *Methods* and *SI Appendix*). To match this architecture, similarity-matching takes place between ORN axon and LN activities, which seeks to maintain that distances (similarities) between neural representations at the level of ORN axons and LNs. Specifically, if the representations of two odors are similar (dissimilar) in ORN axons, their representations will also tend to be similar (dissimilar) in LNs. Second, we derive the circuit models (Eqs. **5**–**7**) that solve this optimization problem with the recorded ORN soma activity described above (5) as input. Third, we compare the synaptic weight organization of the circuit model with the connectome (4) (Figs. 2, 3, and 4) and find that the circuit model accounts for multiple experimental observations. We thus conclude that the similarity-matching principle and the optimization problem widely explain the biological circuit’s organization. Lastly, we describe in detail the computation performed by the circuit model (Figs. 5 and 6).

Mathematically, given a set of *T* activity patterns in *D* ORN somas as input, ${\left\{x^{\left(t\right)}\right\}}_{t=1...T}$, the optimization provides us as output the activity patterns in the *D* ORN axons ${\left\{y^{\left(t\right)}\right\}}_{t=1...T}$ and *K* LNs ${\left\{z^{\left(t\right)}\right\}}_{t=1...T}$. The circuit model performing the computation of the optimization has the following parameters: $W=\left[w_{1},...,w_{K}\right]:=E\left[y^{\left(t\right)}z^{\left(t\right)}{\;}^{\text{T}}\right]$ and $M={\left\{m_{i,j}\right\}}_{i,j=1...K}:=E\left[z^{\left(t\right)}z^{\left(t\right)}{\;}^{\text{T}}\right]$, which are proportional to the connection weights between ORNs and LNs, and between LNs, respectively. In addition to *K*, the number of LNs, the model contains only one effective parameter $\rho^{2}$, corresponding to the ratio between feedback inhibition and feedforward excitation strengths.

We consider two optimization problems leading to two circuit models, differing in their domain of optimization: 1) a linear circuit, LC-*K* with *K* LNs, Eq. **6**, with no constraint on the optimization domain; 2) a nonnegative circuit, NNC-*K*, Eq. **7**, with nonnegative constrains on ORN axon and LN activity ($y^{\left(t\right)}\geq 0,z^{\left(t\right)}\geq 0$). This constraint renders the NNC more biologically plausible than the LC, and the NNC indeed predicts the structural data better than the LC (below). However, only for the LC we can derive the analytical expressions for W, M, $\left\{y^{\left(t\right)}\right\}$, and $\left\{z^{\left(t\right)}\right\}$, whereas for the NNC we must rely on numerical simulations (*SI Appendix*). Because both models are closely related, we examine the analytical solution of the LC to quantitatively understand the relationship between input and output variables, describe the circuit’s function in a mathematically tractable manner, and substantiate the numerical results for the NNC.

Given an input $\left\{x^{\left(t\right)}\right\}$, the optimal synaptic weights can be found by solving the optimization problem offline (Eqs. **4** and **5**), or online with Hebbian plasticity (Eq. **8**). The latter implies that the circuit model’s synaptic weights can adapt to solve the optimization problem on any ORN activity patterns ensemble, in an unsupervised manner. This would correspond to activity-dependent synaptic plasticity in the biological circuit, which was, so far, only observed in the adult *Drosophila* (16–19). Given the specific wiring of some LNs such as Keystone and Picky 0 in the biological circuit (4), it is very likely that the synaptic weights of these (and potentially other) LNs are largely genetically predetermined and were set over evolutionary time scales (similar to an offline setting). It is unknown which mechanisms determine the synaptic weights in the biological circuit, and it is beyond the scope of this study to elucidate them.

Next, we characterize the computation performed by the LC and the NNC as well as the connectivity (in terms of W and M) that supports the computation. In short, in the LC, LNs extract and encode the top *K* PCA subspace of the input in ORN somas and the ORNs → LN synaptic weight vectors $\left\{w_{k}\right\}$ span that subspace. In the NNC, LNs encode soft cluster/feature memberships of the odor representations in ORN somas and $\left\{w_{k}\right\}$ are related to cluster locations. In both models, the ORN axons encode a partially whitened and normalized version of the ORN soma activity due to LN feedback inhibition.

### Predictions of the ORN–LN Connection Weight Vectors.

We start by analyzing our models’ predictions in terms of circuit connectivity. In the LC-*K*, the ${\left\{w_{k}\right\}}_{k=1...K}$ (proportional to the ORNs ↔ LN connection weight vectors) are linearly independent and span the same *K* dimensional subspace as the top *K* PCA directions ${\left\{u_{X,i}\right\}}_{i=1...K}$ of the uncentered input $\left\{x^{\left(t\right)}\right\}$ (*SI Appendix*):

[1] $$\begin{array}{c} w_{k}=\sum_{i=1}^{K}a_{k,i}u_{X,i}. \end{array}$$

This ensures that LNs extract the top *K* PCA subspace of the input (below). The ${\left\{a_{i,j}\right\}}_{i,j=1...K}$ are coefficients with a degree of freedom, arising from the nonuniqueness of the optimization solution. Thus, the $w_{k}$s do not necessarily correspond to specific PCA directions of the input and are not orthogonal. Because the model predictions rely on “uncentered PCA,” i.e., PCA without prior centering of the data, we use such PCA throughout the paper.

We probe this structural prediction by testing the alignment between the four ORNs →LN synaptic count vectors, $\left\{w_{\text{LNtype}}\right\}$ and the first 5 PCA directions of the ORN activity data, ${\left\{x^{\left(t\right)}\right\}}_{\text{data}}$ (Fig. 2*E*, *F*, and *H*). We find that only $w_{\text{BT}}$ is significantly correlated with the first PCA direction. Because this is uncentered PCA, this direction closely resembles the mean activity direction. We compare with the top 5 (instead of 4, as the number of $w_{\text{LNtype}}$s) PCA directions to account for the potential discrepancy between this ORN activity dataset and the true ORN activity.

Next, to test Eq. **1** directly, we examine the alignment of the subspaces spanned by the four $w_{\text{LNtype}}$s and the top five PCA directions of ${\left\{x^{\left(t\right)}\right\}}_{\text{data}}$ (*SI Appendix*, Fig. S7). While ≈1 more dimension is significantly aligned than is randomly expected, supporting the results of Fig. 2*H*, there is no complete alignment. In summary, although $w_{\text{BT}}$ aligns with the top PCA direction of ${\left\{x^{\left(t\right)}\right\}}_{\text{data}}$, and the connectivity and activity subspaces are more aligned than expected by chance, the LC does not account for the connectivity of most LN types.

Next, we study the ${\left\{w_{k}\right\}}_{k=1...4}$ predicted by the NNC-4 (K=4 as the number of LN types) optimized on ${\left\{x^{\left(t\right)}\right\}}_{\text{data}}$ (Fig. 2*A*), for 0.1≤ρ≤10. For ρ⪅3.1, three of the four $w_{k}$s align significantly with a $w_{\text{LNtype}}$ (BT, BD, and P0, Fig. 3 *A* and *B*). In a perfect fit between model and data, each $w_{\text{LNtype}}$ is aligned one $w_{k}$. $w_{\text{KS}}$ is not significantly correlated with any of the $w_{k}$s, but NNC-5 has one $w_{k}$ significantly aligned with $w_{\text{KS}}$ (*SI Appendix*, Fig. S6*H*). The significant alignment of $w_{4}$ with both $w_{\text{BT}}$ and $w_{\text{P0}}$ could arise due to partial correlation between $w_{\text{LNtype}}$s (Fig. 1*C*). Furthermore, we find a similarity between the model and the data in terms of alignment of the ORNs → LN connection weight vectors with the ORN activity vectors ${\left\{x^{\left(t\right)}\right\}}_{\text{data}}$ (*SI Appendix*, Fig. S8).

In summary, the ORN → LN connection weights predicted by the NNC model strongly resemble the synaptic counts in $\left\{w_{\text{LNtype}}\right\}$, but do not provide an exact one-to-one correspondence. This analysis confirms that all the $w_{\text{LNtype}}$s are adapted to ORN activity patterns. It also corroborates the hypothesis that the similarity-matching principle and the optimization problem have explanatory power for the organization of the biological circuit. Later we discuss the potential reasons for the nonexact alignment between the model and the data.

### Emergence of LN Groups in the NNC.

In the connectome, LNs are grouped by type and several $w_{\text{LN}}$s are similar (Figs. 1 *B* and *C* and 3*C*). Do LN groups naturally emerge in our models? In the LC, ${\left\{w_{k}\right\}}_{k=1...K}$ spans the top *K*-dimensional principal subspace of the input $\left\{x^{\left(t\right)}\right\}$, resulting in distinct $w_{k}$s and thus no LN group emerges.

In the NNC, however, we observe the formation of LN groups. For example, in NNC-8 (8 LNs as on each side of the larva) trained on ${\left\{x^{\left(t\right)}\right\}}_{\text{data}}$, several $w_{k}$s are similar, especially for smaller ρ (Fig. 3*D*). Given that the $w_{k}$s point toward the cluster locations in the ORN axon activity space, the grouping of $w_{k}$s is influenced by 1) ORN activity pattern statistics (closer clusters elicit more aligned $w_{k}$s), 2) the number of LNs (having more LNs than clusters lead to several similar $w_{k}$s), and 3) the value of ρ (higher ρ leads to more separated clusters in ORN axons and thus dissimilar $w_{k}$s) (*SI Appendix*, Figs. S9 and S10).

For the biological circuit, we lack exact measures of the factors (e.g., $\left\{x^{\left(t\right)}\right\}$ and ρ) that influence $\left\{w_{k}\right\}$ grouping. Nevertheless, we inquire whether NNC-8 can, in principle, generate a $w_{k}$ grouping similar to the biological circuit for different values of ρ. At ρ=0.35, the mean rectified correlation coefficient ${\bar{r}}_{+}$ ($r_{+}:=\max\left[0,r\right]$) between all $w_{k}$s of the NNC-8 matched that of the connectome (Fig. 3*E*). While this value of ρ, which corresponds to a relatively low feedback inhibition in the model, should not be interpreted as the “true” value in the actual biological circuit, it falls within the range found above (ρ⪅3.1).

In summary, within a reasonable parameter range, the NNC reproduces another property of the biological circuit: the emergence of LN groups.

### Relation between LN–LN and Feedforward ORNs → LN Connection Weights.

The ORN-LN circuit contains reciprocal inhibitory LN–LN connections (Fig. 4*A*) whose connectivity patterns and roles are not fully understood. In our models, these connections are symmetric: the synaptic weights ${\text{LN}}_{i}$→${\text{LN}}_{j}$ and ${\text{LN}}_{j}$→${\text{LN}}_{i}$ are equal. This is largely verified in the connectome, except for the P0, which inhibits the KSs, but is not strongly inhibited by them. Theoretical predictions of the LC-*K* model (with *K* LNs) state that the strength of LN–LN connections ($M={\left\{m_{\text{LNi, LNj}}\right\}}_{i,j=1...K}$) and ORN–LN connections ($W=[w_{1},...,w_{K}]$) are related (*SI Appendix*):

[2] $$\begin{array}{c} M^{2}=M^{⊤}M\propto W^{⊤}W\Leftrightarrow M\propto {\left(W^{⊤}W\right)}^{1/2}, \end{array}$$

where ⊤ is the matrix transpose. This relationship is exact for the LC and approximate for the NNC. The ith column of M, $m_{i}$, is the LNs → LN_i_ (and LN_i_→ LNs) synaptic weight vector. The ith column of W, $w_{i}$, is proportional to the ORNs → LN_i_ (and LN_i_→ ORNs) synaptic weight vector. From Eq. **2** follows that: 1) $\left|\right|w_{i}\left|\right|/\left|\right|m_{i}\left|\right|=\text{const}$, i.e., the ratio between the magnitude of the ORNs → LN and LNs → LN synaptic weight vectors is the same at each LN. The magnitude is a proxy for the total synaptic strength of a synaptic weight vector. 2) $∡\left(w_{i},w_{j}\right)=∡\left(m_{i},m_{j}\right)$, where ∡(a,b) is the angle between two vectors a and b. Thus 2 LNs with a similar (different) connectivity pattern with the ORNs have a similar (different) connectivity pattern with LNs.

We test whether Eq. **2** holds in the connectome (Fig. 4), and find a significant correlation (r=0.73, P=0.006) between the off-diagonal entries of matrices M and ${\left(W^{\text{T}}W\right)}^{1/2}$, suggesting a meticulous co-organization of the ORN–LN and LN–LN connections. We lack the values of the LN neural leaks, which correspond to the diagonal entries of M (Eqs. **6** and **7**).

In summary, the synaptic weight organization in the NNC model resembles that the connectome in several key ways: the synaptic counts $w_{\text{LNtype}}$, the emergence of LN groups, and the relationship between ORNs → LN and LN–LN. The LC model, on the other hand, fails at explaining several of these structural features.

### Circuit Model Computation and Coding Efficiency.

We next explore the computations of the LC and NNC. In both models, upon ORN soma activation, the computation is implemented dynamically through the ORN–LN loop and converges exponentially to a steady state (Eqs. **6** and **7**). Given inputs $\left\{x^{\left(t\right)}\right\}$, the circuit’s outputs are the converged representations in ORN axons, $\left\{y^{\left(t\right)}\right\}$, and LNs, $\left\{z^{\left(t\right)}\right\}$.

Efficient encoding of odor representations in ORN is crucial for downstream processing. Odor representations can be visualized as points in a neural space, where each axis is the activity of an ORN. We consider a circuit with just D=2 ORNs and K=2 LNs, and an artificial input dataset of two odors *A* and *B* (Fig. 5 *A* and *D*). Given $x^{A}$ and $x^{B}$ the representations of the two odors: the larger the angle $∡(x^{A},x^{B})$, the easier the two odors can be discriminated, and the more efficiently the space is utilized. We quantify the efficiency of the encoding by the coefficient of variation of the PCA variances, $\left\{\sigma_{i}^{2}\right\}$, of the representation: ${\text{CV}}_{\sigma }=\text{SD}[\left\{\sigma_{i}^{2}\right\}]/\text{mean}\left[\left\{\sigma_{i}^{2}\right\}\right]$. If all the variances are equal (${\text{CV}}_{\sigma }=0$), the representation is white, and the encoding space is efficiently used (38). A larger ${\text{CV}}_{\sigma }$ indicates a less optimal space utilization. We study the PCA variances and “whiteness” of uncentered data because we assume downstream neurons experience uncentered activity. We further describe the computation in terms of the modification of the stimulus representations.

### LC: Extraction of the Principal Subspace by LNs and Partial Equalization of PCA Variances in ORN Axons.

We first describe the computation in the LC. Given activity patterns $\left\{x^{\left(t\right)}\right\}$ in the *D* ORN somas, we call $\left\{u_{X,i}\right\}$ and $\left\{\sigma_{X,i}^{2}\right\}$ (i=1,...,D) the PCA directions and variances of the uncentered $\left\{x^{\left(t\right)}\right\}$ (Fig. 5*D*). The activity of the *K* LNs, $\left\{z^{\left(t\right)}\right\}$, encodes the top *K* PCA subspace of $\left\{x^{\left(t\right)}\right\}$, i.e., spanned by ${\left\{u_{X,i}\right\}}_{i\leq K}$ (Fig. 5*B*). How exactly LNs encode the subspace is a degree of freedom of the optimization, and thus the activity of individual LNs does not necessarily align with the PCA directions of the input. When K<D, LNs perform a dimensionality reduction of the ORN soma activity.

LNs inhibit ORN axons, altering their odor representation $\left\{y^{\left(t\right)}\right\}$ (Fig. 5*D*). However, the PCA directions $\left\{u_{Y,i}\right\}$ of ORN axon activity remain the same as in ORN somas, i.e., $\left\{u_{Y,i}\right\}=\left\{u_{X,i}\right\}$. Thus, this transformation from soma to axons only stretches and does not rotate the cloud of representations in the neural space. This absence of rotation (called “zero-phase”) makes the axonal and somatic activity maximally similar (23). This is advantageous for downstream processing because the evolving representation in ORN axons, computed dynamically via LN activation, is thus maximally close to the converged representation, allowing meaningful downstream processing before the complete representation convergence.

The PCA variances $\left\{\sigma_{Y,i}^{2}\right\}$ and $\left\{\sigma_{Z,i}^{2}\right\}$ of $\left\{y^{\left(t\right)}\right\}$ and $\left\{z^{\left(t\right)}\right\}$ are (Fig. 5 *D* and *F*):

$$\{\begin{array}{ll} \sigma_{Y,i}\left(1+\rho^{2}\sigma_{Y,i}^{2}\right)=\sigma_{X,i} & 1\leq i\leq K\;\;\;[3\text{a}]\\ \sigma_{Y,i}=\sigma_{X,i} & K+1\leq i\leq D\;[3\text{b}]\\ \sigma_{Z,i}=\rho \sigma_{Y,i} & 1\leq i\leq K.\;\;\;\;[3\text{c}] \end{array}$$

Hence, the variances of the last *D*–*K* PCA directions in ORN somas ($\left\{x^{\left(t\right)}\right\}$) remain unaltered in ORN axons ($\left\{y^{\left(t\right)}\right\}$). The variances of top *K* PCA directions in ORN somas are diminished according to Eq. 3a (Fig. 5 *G* and *H*): relatively large PCA variances in ORN somas ($\sigma_{X,i}^{2}\gg \rho^{2}$) are shrunken with a cubic root in ORN axons ($\sigma_{Y,i}\approx \sqrt[{3}]{\sigma_{X,i}/\rho^{2}}$), relatively small PCA variances ($\sigma_{X,i}^{2}\ll \rho^{2}$) remain virtually unchanged ($\sigma_{Y,i}\approx \sigma_{X,i}$). The PCA variances in LN activity ($\left\{z^{\left(t\right)}\right\}$) are proportional to those in ORN axon activity ($\left\{y^{\left(t\right)}\right\}$) (Eq. 3c). (Note the indices *i* of the PCA directions and variances in ORN axons have been set to match those in ORN somas, and do not follow the usual decreasing order).

This transformation generally results in a smaller coefficient of variation of PCA variances, ${\text{CV}}_{\sigma }$, in the output $\left\{y^{\left(t\right)}\right\}$ than in the input $\left\{x^{\left(t\right)}\right\}$ (*SI Appendix*, see below, Fig. 6*D*). The PCA variances are then less spread and the odor representations are encoded more efficiently. Because the PCA variances are partially equated and no rotation occurs, this transformation is a partial (Zero-phase) ZCA-whitening.

### NNC: Clustering by LNs and Partial Equalization of PCA Variances in ORN Axons.

We next explore the computation of the NNC, where LN ($\left\{z^{\left(t\right)}\right\}$) and ORN axon ($\left\{y^{\left(t\right)}\right\}$) activities are nonnegative. LNs implement symmetric nonnegative matrix factorization (SNMF) on ORN axon activity, which consists of clustering and feature discovery (*SI Appendix*) (37). SNMF is essentially “soft” *K*-means clustering, allowing inputs to belong to multiple clusters. Clustering satisfies the optimization’s objective of nonnegative LN activity and maximally conserved distances between stimulus representations in ORN axons and LNs. Thus LN activity, $\left\{z^{\left(t\right)}\right\}$, encodes the cluster membership of odor representations in ORN axons ($\left\{y^{\left(t\right)}\right\}$), and the ORN → LN synaptic weight vectors, $\left\{w_{k}\right\}$, point toward clusters (Fig. 5 *C* and *E*). Unlike the LC, there is no degree of freedom in LN activity.

The activity in ORN axons in NNC resembles that in LC, only without negative values, and the PCA variances are also similar (Figs. 5*D*–*F*).

### Circuit Model Computation on the ORN Activity Dataset.

Next, to better comprehend the potential computation of the ORN-LN circuit, we study the computation of the NNC on the dataset of odor representation in ORNs, ${\left\{x^{\left(t\right)}\right\}}_{\text{data}}$ (Fig. 2*A*). We also show the LC. We set the parameter that regulates the inhibition strength ρ=2 to clearly represent the effect of the odor representation transformation in ORNs. *K*, the number of LNs, is set to 1, 4 (as the number of LN types) or 8 (as the number of LNs on one side of the larva). We also examine the computation of a nonnegative circuit model (NNC-conn) with connectivity weights proportional to the synaptic counts of the connectome (*SI Appendix*). Because for NNC-conn multiple unknown model parameters need to be guessed, and this circuit might not be adapted to the specific statistics of ${\left\{x^{\left(t\right)}\right\}}_{\text{data}}$, its computation might not accurately reflect that of the true circuit, and the discrepancies with the normative models might be a consequence of this. Nevertheless, we find many similarities between NNC-conn and NNC-8, further supporting our predictions regarding circuit computation. Fig. 6 exhibits the main results, *SI Appendix*, Figs. S13, S14, and S15 display additional analysis of the LC, NNC, and NNC-conn, respectively.

As above, LNs in the LC encode the top *K*-dimensional PCA subspace of ORN soma activity (*SI Appendix*, Fig. S11*B*). LNs in the NNC softly cluster odors, as observed by their sparser activity and their correspondence with ORN activity patterns (Fig. 6*A*). LN activity in NNC-conn is also rather sparse.

In all models, ORN axon activity ($\left\{y^{\left(t\right)}\right\}$) is weaker than in somas (Fig. 6*B*). While it is also sparser and nonnegative in the NNC models, in the LC, it contains negative values, which may not be biologically plausible.

Next, we compare the PCA variances of the odor representations in ORN somas ($\left\{\sigma_{X,i}^{2}\right\}$) and axons ($\left\{\sigma_{Y,i}^{2}\right\}$) (Fig. 6*C*). In the NNC models, variances decrease for all PCA directions. In the LC, however, only the variances of the top *K* PCA directions decrease. This difference results from the nonnegativity constraint in the NNC models, which affects all stimulus directions. The spread of PCA variances $\left\{\sigma_{Y,i}^{2}\right\}$ decreases in all models (smaller ${\text{CV}}_{\sigma }$, Fig. 6*D*) indicating a whiter representation in the ORN axons. This effect is the weakest in the NNC-conn. Changing the number of LNs impacts the NNC less than the LC. In the LC, only the order of the PCA directions of $\left\{x^{\left(t\right)}\right\}$ and $\left\{y^{\left(t\right)}\right\}$ changes, because *K* of them are shrunken (*SI Appendix*, Fig. S12 *A* and *B*). For the NNC, the PCA directions are slightly altered, but their order mostly remains (*SI Appendix*, Fig. S12 *C* and *D*). In the NNC-conn, the PCA directions are modified more strongly (*SI Appendix*, Fig. S12*E*).

Considering the decreased spread of PCA variances, we inquire whether activity becomes more evenly distributed among ORNs, an important property of efficient coding. Both the LC and NNC decrease the (uncentered) activity variance of “high-variance ORNs” and leave “low-variance ORNs” virtually unaffected, reducing the CV of ORN variance (Fig. 6 *E* and *F*). The NNC-conn, however, exhibits an increase in CV due to several “high-variance ORNs” being not strongly dampened.

Subsequently, we investigate changes in the magnitude of ORN soma and axon activity patterns. The magnitude is the length of an activity pattern vector in the D=21 dimensional ORN space and is a proxy for the total activity of all ORNs in response to an odor. Similarly to ORN variances, the magnitude of large-magnitude patterns decreases, whereas small-magnitude patterns remain unchanged, decreasing the spread of pattern magnitudes (Fig. 6 *G* and *H*). These effects resemble a divisive normalization-type computation, also reported in *Drosophila* (13, 25).

In line with the less dispersed PCA variances in ORN axons, in all models ORNs and odor representations are more decorrelated in the axons than in the somas (Fig. 6*I*–*L*), consistent with partial whitening.

Additionally, we investigate the effect of adjusting the model parameter ρ, which regulates feedback inhibition strength. A higher ρ (ρ=10, *SI Appendix*, Fig. S16) leads to decreased activity in ORN axons and smaller PCA variances, reduced spread of PCA variances, channels and patterns norms, stronger decorrelation of ORNs and patterns. When inhibition is eliminated (ρ→0), the axonal and somatic ORN activity become identical. Although it is unknown if inhibition is modulated in the real circuit, altering this parameter allows us to understand this circuit’s potential.

In summary, NNC analysis predicts that the ORN-LN circuit clusters odors with LNs and performs partial ZCA-whitening and normalization of odor representations in ORN axons. This results in a more efficiently encoded output with more decorrelated and equalized ORNs and odor representations, ultimately enhancing odor discrimination downstream.

### Computation without LN–LN Connections.

Lastly, we investigate the role of LN–LN connections by considering two alternative circuit models. First, we consider an LC or NNC circuit adapted to an input ensemble (i.e., Fig. 6) and remove the LN–LN connections, which corresponds to setting the off-diagonal elements in M to 0 (*SI Appendix*, Fig. S17). This manipulation leads to less sparse LN activity in the NNC, altered PCA directions in the axonal activity relatively to the soma, increased inhibition, and more dissimilar odor representations in ORN axons compared to somas. Thus, in an already “adapted” circuit, LN–LN connections improve clustering in LNs for the NNC, regulate inhibition, and maintain similar representation in ORN axons and somas.

Second, we consider the slightly different optimization problem that leads to an ORN-LN circuit without LN–LN connections (*SI Appendix*) (39). In the linear case, the whitening is complete (i.e., the first K PCA variances that are larger than $1/\rho^{2}$ become equal) and the *K* LNs still encode the top *K* dimensional subspace of the input. However, with nonnegativity constraints on ORN axon and LN activity, all LNs display the same activity, lacking differentiation (*SI Appendix*, Fig. S18). Thus, in this case, LN–LN connections are imperative for clustering.

## Discussion

Combining the *Drosophila* larva olfactory circuit connectome, ORN activity data, and a normative model, we advance the understanding of sensory computation and adaptation, quantitatively link ORN activity statistics, functional data, and connectome, and make testable predictions. We reveal a canonical circuit model capable of autonomously adapting to different environments, while maintaining the critical computations of partial whitening, normalization, and feature extraction. Such a circuit architecture may arise in other brain areas and may be applicable in machine learning and signal processing. Using ORN activity patterns as input, our normative framework accounts for the biological circuit structural organization and identifies in the connectome signatures of circuit function and adaptation to ORN activity. Such an approach offers a general framework to understand circuit computation (40, 41) and could provide valuable insights into more neural circuits, whose structural and activity data become available (1, 2).

### Model and Biological Circuit: Similarities and Differences.

In this paper, we compare the structural predictions of our normative approach to the connectome. The NNC model, when adapted to the ORN activity dataset (5), accounts for key structural characteristics (Figs. 3 and 4), for example, the ORNs → LN connection weight vectors. We ask two questions: 1) Why does the strong resemblance between model and data arise, when the available odor dataset most probably imperfectly matches the true larva odor environment? 2) Why isn’t the resemblance even greater, and could the imperfect fit suggest that the model inadequately explains the biological circuit?

For 1), a possibility is that generic correlations between ORNs arise in large enough ORN activity datasets, causing robust features in the model connectivity. These correlations could result from the intrinsic chemical properties of ORN receptors. Odor statistics would also influence the connection weights, but to a lesser degree. Thus, a more naturalist activity dataset could further improve model predictions.

For 2), first, due to intrinsic noise and variability, no model could be 100% accurate in predicting connectivity. In fact, variability in synaptic count and innervation arises for *Drosophilas* raised in similar environments (27, 42), indicating potential “imprecision” of development and/or learning. We also observe variability in the left vs. right side connectivity (Fig. 1*B*). Second, incomplete ORN activity statistics may decrease prediction accuracy. Third, synaptic count might not exactly reflect synaptic strength (11). Finally, our model being a simplification of reality misses additional factors shaping circuit connectivity.

Our analysis indicates that the matches between model and data likely do not result from chance only, suggesting that the similarity-matching principle influences circuit organization. However, our unsupervised approach assumes that no odor is “special” for the animal, and thus LNs in the circuit model cluster odors solely based on their representations in the ORN activity space. This contrasts with the biological ORN-LN circuit, where LNs such as Keystone and Picky 0 have specific downstream connections likely related to survival needs and different hardwired animal behaviors (4, 43), requiring them to detect particular odors. Consequently, the connectivity of such LNs might contribute to the imperfect one-to-one correspondence between the model and the connectome (e.g., KS in NNC-4, Fig. 3*A*).

The circuit model can learn the optimal connection weights autonomously via Hebbian learning, offering the capacity to adapt to different environments. Studies in adult *Drosophila* reveal that glomeruli sizes (and thus ORN–LN or ORN–PN synaptic weights) or activity depend on the environment in which the *Drosophila* grew up (16–19). It is, however, unknown if activity-dependent plasticity also occurs in the larval ORN-LN circuit and whether the observed synaptic counts are a result of such plasticity. If present, it is unclear whether the short 6-h life of the larva from which the connectome was reconstructed allows substantial learning to occur and whether changes in synaptic weights would translate to different synaptic counts (11).

Resolving connectomes of larvae raised in different odor environments and at different times of their life, probing synaptic plasticity, and recording ORN responses to the full odor ensemble present in its environment would help clarify the influence of noise, plasticity, and genetics in circuit shaping.

### Roles of LNs.

LNs form a significant part of the neural populations in the brain, perform diverse computational functions, and exhibit extremely varied morphologies and excitabilities (27, 44). We propose a dual role for LNs in this olfactory circuit: altering the odor representation in ORNs and extracting ORN activity features, available for downstream use (4). In the olfactory system of *Drosophila* and zebrafish, LNs perform multiple computations, such as gain control, normalization of odor representations, and pattern and channel decorrelation (12–15, 32, 45), which is consistent with our results. Also, in *Drosophila* the LN population expands the temporal bandwidth of synaptic transmission and temporally tunes PN responses (28, 29, 46), which was not addressed here.

In topographically organized circuits, such as in the visual periphery or in the auditory cortex, distinct LN types uniformly tile the topographic space, and each LN type extracts a specific feature of the input, e.g., in the retina (47). In nontopographically organized networks, however, the organization and role of LNs remains a matter of research and controversy (27, 48). We study a subcircuit with four LN types, and most types contain several similarly connected LNs (Fig. 1). What is the function of multiple similar LNs in the ORN-LN circuit, as also observed in the NNC (Fig. 3*C*–*E*)? First, LNs might differentiate further as the larva grows. Second, several LNs might help expand the dynamic range of a single LN. What are the features extracted by LNs in the *Drosophila* larva? Our NNC model and the distinct connectivity patterns of LN types in the connectome (4), suggest that different LN types are activated in response to different sets of odors. The extracted features might relate to clusters in ORN activity and to prewired, animal-relevant odors. Since several ORNs → LN connection weight vectors $\left\{w_{k}\right\}$ in the NNC model resemble those in the biological circuit, the odor clusters identified by the model likely correspond to the set of odors that activate LNs in the biological circuit. The feedforward synaptic count vector from ORNs to the Broad Trio $w_{\text{BT}}$, which aligns with the first PCA direction of ORN activity and with an ORNs → LN connection weight vector $w_{k}$ in the NNC model (Figs. 2*H*, 3 *A* and *B*) could potentially encode the mean ORN activity and thus be related to the global odor concentration (26). Other LNs might encode features of odors, such as aromatic vs. long-chain alcohols (5), or specific information influencing larva behavior (4, 43), but more experiments are required to definitely resolve the features. While our conclusions differ from a study that found that LN activation is invariant to odor identity (48), that study imaged several LNs simultaneously and might thus have missed the selectivity of individual LNs.

The connectome reveals LN–LN connections, which we propose play a key role in clustering and shaping the odor representation, and are co-organized with the ORN–LN connections (Fig. 4). To the best of our knowledge, the role of LN–LN connections and their relationship to ORN–LN connections is relatively unexplored.

In summary, our study emphasizes the importance of the different ORN–LN and LN–LN connection strengths and argues that LNs are minutely selective and organized to extract features and render the representation of odors more efficient.

### Circuit Computation, Partial ZCA-Whitening, and Divisive Normalization.

We propose that the circuit’s effect on the neural representation of odors in ORNs corresponds to partial ZCA-whitening and divisive normalization (Figs. 5 and 6). Such computations, which reduce correlations originating from the sensory system and the environment, have appeared in efficient coding and redundancy reduction theories (22, 25, 36, 38, 49, 50). Partial whitening is in fact a solution to mutual information maximization in the presence of input noise (38). In this circuit too, complete whitening might also not be desirable due to potential noise amplification. Thus, keeping low-variance signal directions of the input unchanged and dampening larger ones is consistent with mutual information maximization. Our conclusions are in line with reports of pattern decorrelation and/or whitening in the olfactory system in zebrafish (14, 15, 32, 33) and mice (34, 35).

The computation in our model also resembles divisive normalization, an ubiquitous computation in the brain (25), proposed for the analogous circuit in the adult *Drosophila* (12, 13). In its simplest form, divisive normalization is defined as $Y_{j}=\alpha X_{j}^{n}/\left(\sigma^{n}+\sum_{k}X_{k}^{n}\right)$, where $Y_{j}$ is the response of neuron *j*, $X_{i}$ is the driving input of neuron *i*, α is the maximum response of the output neuron and σ and *n* determine the offset and slope of the neuronal sigmoidal response curve, respectively (25). Divisive normalization captures two effects of neuronal and circuit computation: 1) neural response saturation with increasing input up to a maximum spiking rate α, arising from the neuron’s biophysical properties; 2) dampening of the response of a given neuron when other neurons also receive input, often due to lateral inhibition (but see ref. 51). Aspect (1) is absent in our model but could be implemented with a saturating nonlinearity. Depending on the biological value of the maximum output, our model might not accurately capture responses for high-magnitude inputs. However, signatures of (2) are evident in the saturation of the activity pattern magnitudes in ORN axons for increasing ORN soma activity pattern magnitudes (Fig. 6*G*). Activity patterns of large magnitude correspond to activity at higher odor concentrations and with a high number of active ORNs. Because such input directions are more statistically significant in our dataset, these stimuli are more strongly dampened by LNs (which encode such directions) than those with few ORNs active. Thus, our model presents a possible linear implementation of a crucial aspect of divisive normalization, which in itself is a nonlinear operation.

Although the basic form of divisive normalization performs channel decorrelation, and not activity pattern decorrelation (13, 14, 32), our models perform both channel and pattern decorrelation. Nevertheless, a modified version of divisive normalization, which includes different coefficients for the driving inputs in the denominator (52), performs pattern decorrelation too, as our circuit model. The proposed neural implementations of divisive normalization usually require multiplication by the feedback (52, 53), which might not be as biologically realistic as our circuit implementation.

Several neural architectures similar to ours have been proposed to learn to decorrelate channels, perform normalization, or learn sparse representations in an unsupervised manner (21, 37, 52, 54–59). However, these studies either lack a normative/optimization approach or have a different circuit architecture or synaptic learning rules. Using a normative approach has the advantage of directly investigating the underlying principles of neural functioning and also potentially providing a mathematically tractable understanding of the circuit structure and function.

Our study complements machine learning approaches to understand neural circuit organization (60, 61). These approaches use supervised learning and backpropagation to train an artificial neural network to perform tasks such as odor or visual classification. In the olfactory system, circuit configurations arising from this optimization, which could mimic the evolutionary process, display many connectivity features found in biology (61). Unlike these approaches, we propose a general principle governing the transformation of neural representations, similarity-matching, and also a mechanism to learn autonomously during the animal’s lifetime.

## Materials and Methods

### Optimization Problems Describing the ORN-LN Circuit.

We use a normative approach to study the ORN-LN circuit. We formulate two optimization problems that can be solved by a circuit model with the ORN-LN architecture. Studying the circuit model computation is then equivalent to studying the solution of an optimization problem. We derive analytical expressions describing different aspects of the computation and the circuit synaptic organization (*SI Appendix*).

We define the following variables: an input matrix $X=[x^{\left(1\right)},...,x^{\left(T\right)}]$ of *T* samples, and outputs $Y=[y^{\left(1\right)},...,y^{\left(T\right)}]$, $Z=[z^{\left(1\right)},...,z^{\left(T\right)}]$. $x^{\left(t\right)}$ and $y^{\left(t\right)}$ are *D*-dimensional vectors, while $z^{\left(t\right)}$ are *K*-dimensional. $x^{\left(t\right)}$, $y^{\left(t\right)}$, and $z^{\left(t\right)}$ represent the activity patterns of *D* ORN somas (i.e., the inputs), *D* ORN axons and *K* LNs, respectively. We call $b^{*}$ an optimal value (solution) of a variable *b*. In the results section, we drop the ${}^{*}$. We postulate the following similarity-matching-inspired optimization problem (e.g., ref. 20), which seeks the optimal output activities $Y^{*}$ and $Z^{*}$ given an input X:

[4] $$\begin{array}{c} \min_{Y}\max_{Z}\;\frac{T}{2}\left||X-Y\right|{|}_{F}^{2}-\frac{\rho^{2}}{4}\left||Y^{\text{T}}Y-\frac{1}{\rho^{2}}Z^{\text{T}}Z\right|{{|}_{F}^{2}+\frac{\rho^{2}}{4}\left||Y^{\text{T}}Y\right||}_{F}^{2}, \end{array}$$

where $\left||\cdot \right|{|}_{F}^{2}$ is the square of the matrix Frobenius (Euclidean) norm. The term $\left||X-Y\right|{|}_{F}^{2}$ drives the activity of the ORN axons Y toward the activity of ORN somas X and ensures that $Y^{*}=X$ when there is no activity in the LNs. The terms $\left|\right|Y^{\text{T}}Y-1/\rho^{2}Z^{\text{T}}Z{\left|\right|}_{F}^{2}$ and $\left|\right|Y^{\text{T}}Y{\left|\right|}_{F}^{2}$ align the similarities between the activities of ORN axons and LNs and puts a 4th order penalty on the norm of Y; they correspond to the bidirectional all-to-all connectivity between ORN axons and LNs, as well as between LNs, but no direct connectivity between ORN axons; such similarity-matching terms permit a significant change of neural representation and a change of dimensionality, which takes place between ORN axons and LNs. ρ is a parameter related to the strength of the dampening in Y and affects both the optima $Y^{*}$ and $Z^{*}$.

We consider this optimization in two search domains for Y and Z. One without any constraints on Y and Z, representing the linear circuit (LC) model, and one with nonnegativity constraints (Y≥0, Z≥0), representing the nonnegative circuit (NNC) model. Nonnegativity constraints account for the fact that neural activity is usually nonnegative, or at least not symmetric in the negative and positive directions. The optimal $Y^{*}$ and $Z^{*}$ can be found analytically for the LC, and through numerical simulations for the NNC. Note that one cannot always guarantee converging to a global optimum for the NNC (62).

We prove that a neural circuit with ORN-LN architecture can solve this optimization problem (*SI Appendix*, Online algorithm). In brief, we introduce into the optimization problem two auxiliary matrices $W:=YZ^{\text{T}}/T$ and $M:=ZZ^{\text{T}}/T$, which naturally map onto ORNs–LNs and LNs–LNs synaptic weights, respectively. By construction, M is symmetric, i.e., M = $M^{\text{T}}$. The new objective function is then optimized over the variables $\left\{y^{\left(t\right)}\right\}$, $\left\{z^{\left(t\right)}\right\}$, W, and M. Writing the gradient descent/ascent over $y^{\left(t\right)}$ and $z^{\left(t\right)}$ provides the neural dynamics equations, with W and M related to the synaptic weights (Eqs. **6** and **7**). The optimal $W^{*}$ and $M^{*}$:

[5] $$\begin{array}{c} W^{*}=Y^{*}Z^{{*}^{\text{T}}}/T,\;M^{*}=Z^{*}Z^{{*}^{\text{T}}}/T, \end{array}$$

can be found “offline” by obtaining the optimal $Y^{*}$ and $Z^{*}$ in Eq. **4**, or in the “online setting,” through unsupervised, Hebbian learning, where W and M are updated after each stimulus presentation (Eq. **8**, see below).

### Circuit Neural Dynamics.

A solution to the optimization problem Eq. **4** without the nonnegativity constraints can be implemented by the following differential equations describing the LC, whose steady-state solutions correspond to the optima for $y^{\left(t\right)}$ and $z^{\left(t\right)}$ for given M and W (*SI Appendix*, Online algorithm). These equations naturally map onto the ORN-LN neural circuit dynamics (dropping the sample index (t) for simplicity of notation):

[6] $$\begin{array}{c} \left\{\;\;\begin{array}{c} \tau_{y}dy\left(\tau \right)/d\tau =-y\left(\tau \right)-Wz\left(\tau \right)+x\\ \tau_{z}dz\left(\tau \right)/d\tau =-Mz\left(\tau \right)+\rho^{2}W^{\text{T}}y\left(\tau \right), \end{array}\right. \end{array}$$

where x, y, and z are *D*, *D*, and *K*-dimensional vectors, and represent the activity (e.g., spiking rate) of the ORN somas, ORN axons, and LNs, respectively. $\tau_{y}$ and $\tau_{z}$ are neural time constants, τ is the local time evolution (not to be confused with the *(t)* sample index). The elements of the D×K matrices $\rho^{2}W$ and W contain the synaptic weights of the feedforward ORNs → LN and feedback LN → ORNs connections, respectively. Thus, the feedforward connection vectors are proportional to the feedback vectors, and the parameter ρ sets the ratio. The assumption of proportionality is reasonable considering the connectivity data (*SI Appendix*, Fig. S2 *A*, *B*, and *D*). Off-diagonal elements of the K×K matrix M contain the weights of the LN - LN inhibitory connections, whereas the diagonal entries encode the LN leaks. In the absence of LN activity and at steady state, the equations satisfy y=x, i.e., somatic and axonal activities of ORNs are identical. In the absence of input (x=0) both y and z decay exponentially to 0, because of the terms −y(τ) and $-M_{i,i}z_{i}\left(\tau \right)$, respectively. In summary, these equations effectively model the ORN-LN circuit dynamics by implementing that 1) the ORN axonal activity is driven by the input in ORN somas x and inhibited by the feedback from the LNs through the term −Wz(τ) and 2) LN activity is driven by the activity in ORN axonal terminals by $\rho^{2}W^{\text{T}}y\left(\tau \right)$ and inhibited by LNs through the term −Mz(τ). Note that changing ρ in the objective function leads to different optimal $W^{*}$ and $M^{*}$.

When optimized online, the optimization problem Eq. **4** with the nonnegativity constraints gives rise to the following equations describing the NNC:

[7] $$\begin{array}{c} \left\{\;\;\begin{array}{c} y\left(\tau +1\right)={\left[y\left(\tau \right)+\epsilon \left(\tau \right)\left(-y(\tau )-Wz(\tau )+x\right)\right]}_{+}\\ z\left(\tau +1\right)={\left[z\left(\tau \right)+\epsilon \left(\tau \right)\left(-Mz\left(\tau \right)+\rho^{2}/W^{\text{T}}y\left(\tau \right)\right)\right]}_{+}, \end{array}\right. \end{array}$$

where ϵ(τ) is the step size parameter and ${\left[x\right]}_{+}:=\max\left[0,x\right]$ is a component-wise rectification. Here, τ is a discrete-time variable. These equations are analog to Eq. **6**, but also satisfying constraints on the activity: $\;y_{i}\left(\tau \right)\;\geq \;0,z_{i}\left(\tau \right)\;\geq \;0,\forall \tau ,i$. Such constraints are implemented by formulating circuit dynamics in discrete time and using a projected gradient descent.

We call LC-*K* the linear circuit model implemented by Eq. **6** and NNC-*K* the nonnegative circuit model implemented by Eq. **7**, with *K* LNs.

Note that there is a manifold of implementations of the same computation by a circuit model. First, one can introduce a parameter γ (*SI Appendix*), that scales the feedforward and feedback connections as well as the magnitude of LN activity, in such a way that the ORN axon activity remains the same. Second, multiplying the whole equation in Eq. **6** or Eq. **7** would not alter the converged output, but would scale the circuit time constants and synaptic weights.

### Synaptic Plasticity.

The circuit model is capable of reaching the optimal synaptic weights $W^{*}$ and $M^{*}$, which solve the optimization problem Eq. **4**, in an unsupervised manner, with Hebbian plasticity. In practice, as the circuit receives a stimulus $x^{\left(t\right)}$ (ORN soma activation), it performs a computation that yields a steady state output activity in ORN axons $y^{\left(t\right)}$ and LNs $z^{\left(t\right)}$ (with Eq. **6** or Eq. **7**); the synaptic weights are then updated using Hebbian rules:

[8] $$\begin{array}{c} \begin{array}{cc} W^{\left(t+1\right)} & =W^{\left(t\right)}+\epsilon_{1}\left(t\right)\left(y^{\left(t\right)}z^{{\left(t\right)}^{\text{T}}}-W^{\left(t\right)}\right)\\ M^{\left(t+1\right)} & =M^{\left(t\right)}+\epsilon_{2}\left(t\right)\left(z^{\left(t\right)}z^{{\left(t\right)}^{\text{T}}}-M^{\left(t\right)}\right), \end{array} \end{array}$$

where $\epsilon_{i}\left(t\right)$ are learning rates. These equations arise when optimizing Eq. **4** online. We assume that the ORN soma activation $x^{\left(t\right)}$ is present long enough so that $y^{\left(t\right)}\left(\tau \right)$ and $z^{\left(t\right)}\left(\tau \right)$ reach steady state values. During this iterative process of synaptic updating, where the circuit model “learns”/“adapts” to the stimulus ensemble $\left\{x^{\left(t\right)}\right\}$, the synaptic weights converge toward “optimum” steady state Eq. **5** (which might require multiple learning epochs over the $\left\{x^{\left(t\right)}\right\}$). Note that the neural leaks of LNs (diagonal values of M) are set (Eq. **5**) and updated (Eq. **8**) similarly to the synaptic weights (W and off-diagonal of M).

## Supplementary Material

Appendix 01 (PDF)

## Data Availability

The connectome and activity datasets are available in refs. (4) and (5). Code for generating the analysis and all the figures is available in GitHub (https://github.com/chapochn/ORN-LN_circuit) (63).
