# Supplementary material for: Normative and mechanistic model of an adaptive circuit for efficient encoding and feature extraction
Source: Proc Natl Acad Sci U S A. 2023 Jul 10;120(29):e2117484120. doi: 10.1073/pnas.2117484120 (PMC10629579; doi:10.1073/pnas.2117484120)
Supplement: Supplementary file 1 — Appendix 01 (PDF) [file pnas.2117484120.sapp.pdf]

PNAS
www.pnas.org

Supplementary Information for

Normative and mechanistic model of an adaptive circuit for efficient encoding and feature extraction

Nikolai M. Chapochnikov, Cengiz Pehlevan, Dmitri B. Chklovskii

Nikolai M. Chapochnikov.
E-mail: nchapochnikov@gmail.com

This PDF file includes:

Supplementary text
Figs. S1 to S18
Tables S1 to S2
SI References

Contents

1 ORN-LN circuit connectome **4**

2 ORN activity data (Fig. 2A) **4**

3 Relationship between ORN activity patterns and ORNs $\rightarrow$ LN synaptic count vectors in the data (Fig. 2) **4**
- A Results . . . 4
- B Methods: RCF distribution of correlation coefficient and significance testing . . . 5

4 Number of aligned dimensions between the activity and connectivity subspaces (Fig. S7) **5**
- A Results . . . 5
- B Methods . . . 5

5 Hierarchical clustering for plotting Fig. 3D **6**

6 Optimization problem (Eq. (4) in the main text) **6**
- A Description . . . 6
- B Equivalence of scaling $\mathbf{X}$ and ρ . . . 6
- C Equivalence of scaling $\mathbf{Z}$ and γ . . . 7

7 Offline solution/computation of the optimization problem **7**
- A Solution for the LC (Eq. (3a), Eq. (3b), Eq. (3c) in the main text) . . . 7
- B Proof . . . 8
- C Computation in LNs and relationship between the NNC and SNMF . . . 11

8 Online algorithm and its implementation by a neural circuit with ORN-LN architecture **11**
- A Circuit equations for the LC (Eq. (6) in the main text) . . . 12
- B Circuit equations for the NNC (Eq. (7) in the main text) . . . 13
- C Circuit model with Hebbian synaptic update rules (Eq. (8) in the main text) . . . 13
- D Steady-state solution of the circuit dynamical equations for the LC and stability . . . 14

9 Effect of ρ and γ on the computation and the circuit **15**

10 Circuit dynamics equations contains two effective parameters (ρ and γ) **15**

11 Relationship between W and M (Eq. (2) in the main text) **16**
- A Consequence of the matrix relationship . . . 17

12 Relationship between ORN activity and ORN-LN connectivity (Eq. (1) in the main text) **17**

13 Decrease of the spread of PCA variances in ORN axons vs soma in the LC **17**

14 Numerical simulations of the LC and NNC **18**
- A Numerical simulation of the LC offline . . . 18
- B Numerical simulation of the NNC offline . . . 19
- C Numerical simulation of the circuits online . . . 20

15 Simulation of the circuit with synaptic weights from the connectome (Fig. S15) **21**

16 Optimization problem for circuit without LN-LN connections (Fig. S18) **21**
- A Online solution . . . 21
- B Circuit computation . . . 22
- C Numerical simulations . . . 22

Supplementary figures and tables 23
Fig. S1. Full ORN connectivity and circuit selection . . . 24
Fig. S2. ORN-LN connectivity, comparison feedforward with feedback . . . 25
Fig. S3. ORN soma activity from Si et al., 2019 . . . 26
Fig. S4. Alignment of activity patterns $\mathbf{x}^{(t)}$ in ORNs and ORNs → LN synaptic count vectors $\mathbf{w}_{\text{LNtype}}$ . . . 27
Fig. S5. Alignment of activity patterns $\mathbf{x}^{(t)}$ in ORNs and ORNs → LN synaptic count vectors $\mathbf{w}_{\text{LN}}$ . . . 28
Fig. S6. PCA of ORN activity and NNC connectivity vs data connectivity . . . 29
Fig. S7. Activity and connectivity subspace alignment . . . 30
Fig. S8. Alignment of activity patterns $\{\mathbf{x}^{(t)}\}_{\text{data}}$ in ORNs and connectivity weight vectors $\{\mathbf{w}_k\}$ from NNC-4 31
Fig. S9. Clustering by the NNC and correlation between the $\mathbf{w}_k$ for two separated clusters . . . 32
Fig. S10. Clustering by the NNC and correlation between the $\mathbf{w}_k$ for two nearby clusters . . . 34
Fig. S11. Activity of LNs $\{\mathbf{z}^{(t)}\}$ in the NNC and LC . . . 35
Fig. S12. PCA directions of odor representations at ORN somas vs ORN axons in LC and NNC . . . 36
Fig. S13. Input transformation by LC-1 and LC-8 with $\rho = 2$ . . . 37
Fig. S14. Input transformation by NNC-1 and NNC-8 with $\rho = 2$ . . . 38
Fig. S15. Input transformation by a nonnegative circuit with synaptic weight vectors from the connectome 39
Fig. S16. Input transformation by LC and NNC with $\rho = 10$ . . . 40
Fig. S17. Effect of removing LN-LN connections on the LC and NNC model . . . 42
Fig. S18. LNs in circuit models without LN-LN connections . . . 43
Table S1. Abbreviations . . . 44
Table S2. Mathematical symbols and variables . . . 45

References 46

1. ORN-LN circuit connectome

We use the synaptic counts from the EM reconstruction obtained by (1). We note here several details regarding our usage of the connectome data.

The indices of the Broad Trio (BT) and Broad Duet (BD) are arbitrary, and there is no correspondence between the indices on the left and right sides. Although BT 1 R is of the same type as other BT, its connection vector has a correlation of 0 with other BT in the connectome data.

There are 2 Keystones (KS) in the *Drosophila* larva. One has its soma positioned on the right of the larva, and the other one on the left. We call them KS L and KS R, respectively. Each KS establishes bilateral connections, i.e, it connects with neurons both on the left and right sides of the larva. Therefore, in terms of connectivity, there are effectively 4 KS connections with other neurons. For example there are 4 ORNs $\rightarrow$ KS synaptic count vectors. In the paper, when referring to a connectivity vector, call KS L R the connections of a Keystone with the soma positioned on the left, connecting with the neurons of the right.

The Picky 0 predominately receives synaptic input on the dendrite (relatively to its axon), we thus only consider the connections synapsing onto the dendrite.

2. ORN activity data (Fig. 2A)

We use the average maximal Ca^{2+} $\Delta F/F_0$ responses among trials for the activity data as in (2). For the ORN 85c in response to 2-heptanone, and for the ORN 22c in response to methyl salicylate, we only have responses to dilutions $\leq 10^{-7}$. Because the ORN responses are very similar for dilutions 10^{-7} and 10^{-8} and are already saturated (for this cell we have responses down to dilutions of 10^{-11}), we set the missing response for dilutions 10^{-6}, 10^{-5} and 10^{-4} as the response for 10^{-7}.

3. Relationship between ORN activity patterns and ORNs $\rightarrow$ LN synaptic count vectors in the data (Fig. 2)

A. Results. In this section, we provide further evidence that the ORNs $\rightarrow$ LN synaptic count vectors contain signatures of the ORN activity patterns. In the main text, we show that the ORNs $\rightarrow$ LN synaptic count vectors $\mathbf{w}_{\text{LNtype}}$ for the Broad Trio and the Picky 0 significantly correlate with a subset ORN activation patterns. Fig. S4A shows the distribution of p-values for the correlation between each of the ORNs $\rightarrow$ LN synaptic count vectors $\mathbf{w}_{\text{LNtype}}$ and all the ORN activity patterns $\{\mathbf{x}^{(t)}\}_{\text{data}}$. In the case when the null hypothesis is true (the activity patterns are not more correlated with the connectivity vector than expected by chance) the distribution of p-values is expected to be flat. Here, however, we observe that for the Broad Trio and for the Picky 0, the distribution of p-values is skewed towards small values, confirming a significant alignment of these connection vectors with this ensemble of ORN activity patterns

Our next approach to test whether the synaptic count vectors $\mathbf{w}_{\text{LNtype}}$ contains signatures of the ORN activity patterns is the following: we investigate how well the ensemble of activity patterns $\{\mathbf{x}^{(t)}\}_{\text{data}}$ reconstructs the connectivity vector $\mathbf{w}_{\text{LNtype}}$ in comparison to reconstructing randomly shuffled versions of $\mathbf{w}_{\text{LNtype}}$. As the number of activity patterns $\left\{\mathbf{x}^{(t)}\right\}$ (170) is larger than the dimension of the connectivity vector (21), we add an L1 regularization term on the coefficients of the reconstructions and consider the following lasso linear regression minimization:

$$\min_{\mathbf{v}} \left\| \hat{\mathbf{w}}_{\text{LNtype}} - \sum_{t=0}^{T} v_t \hat{\mathbf{x}}^{(t)} \right\|_2^2 + \lambda \left\| \mathbf{v} \right\|_1 \qquad \text{[S1]}$$

Where $\mathbf{v} = [v_0, ..., v_T]$ is a vector of coefficients, λ encodes the strength of the regularization, $\hat{\mathbf{a}} = \mathbf{a}/\left\|\mathbf{a}\right\|$, $\left\|\mathbf{a}\right\|_2$ is the L2 (Euclidean) norm, and $\left\|\mathbf{a}\right\|_1 = \sum_i \text{abs}(a_i)$ is the L1 norm. Note that we added the constant vector $\mathbf{x}^{(0)} = \mathbf{1}$ since the connectivity and activity vectors are not centered. We make the hypothesis that the connectivity vectors $\mathbf{w}_{\text{LNtype}}$ from the connectome are significantly more accurately reconstructed by the ORN activity vectors than a shuffled version of $\mathbf{w}_{\text{LNtype}}$. We probe the accuracy of the reconstruction by plotting the reconstruction error $\|\hat{\mathbf{w}}_{\text{LNtype}} - \sum_{t=0}^{T} v_t \hat{\mathbf{x}}^{(t)}\|_2$ as a function of the norm of the coefficient vector $\|\mathbf{v}\|_1$. To get different values in this relationship we optimize this objective function for different values of λ for the original $\mathbf{w}_{\text{LNtype}}$ and for the shuffled one. Figs. S4B to E shows that indeed, for the Broad Trio and for the Picky 0 the reconstruction is significantly better than expected by random.

Finally, to test if the $\mathbf{w}_{\text{LNtype}}$ are significantly aligned with the ensemble $\{\mathbf{x}^{(t)}\}_{\text{data}}$, we compare the relative cumulative frequency (RCF) of the correlation coefficients r between each $\mathbf{w}_{\text{LNtype}}$ and all the $\{\mathbf{x}^{(t)}\}_{\text{data}}$ with the RCFs of r obtained after randomly shuffling the entries of each $\mathbf{w}_{\text{LNtype}}$ (Figs. S4F to I). We use the maximum

deviation from the mean RCF from the shuffled connection vector to measure significance (Figs. S4J to M) and find that $\mathbf{w}_{\mathrm{BT}}$ is significantly aligned to $\{\mathbf{x}^{(t)}\}_{\mathrm{data}}$, and that $\mathbf{w}_{\mathrm{P0}}$ is at the edge of the 0.05 significance level (Fig. S4N).

All the above evidence corroborates the hypothesis that the ORNs $\to$ LN synaptic count vectors are adapted to ORN activity patterns.

B. Methods: RCF distribution of correlation coefficient and significance testing. Given a vector $\mathbf{a} \in \mathbb{R}^D$, we define the mean $\bar{a} := \frac{1}{D}\sum_{i=1}^{D} a_i$, the centered vector $\mathbf{a}_c := \mathbf{a} - \bar{a}$, and the centered normalized vector $\widehat{\mathbf{a}} := \mathbf{a}_c/||\mathbf{a}_c||$: We call $\widehat{\mathbf{w}} \in \mathbb{R}^D$ the centered and normalized ORNs $\to$ LN synaptic count vector $\mathbf{w}$. Similarly, we define $\widehat{\mathbf{X}} \in \mathbb{R}^{D\times T}$ the centered and normalized ORN activity $\mathbf{X}_{\mathrm{data}} = [\mathbf{x}^{(1)}, ..., \mathbf{x}^{(T)}]$, where each column vector is centered and normalized.

Each row of the matrix of correlation coefficients depicted in Fig. 2B is given by $\mathbf{c} := \widehat{\mathbf{w}}_{\mathrm{LNtype}}^{\top}\widehat{\mathbf{X}}$. $\mathbf{c}$ is used to calculate the true relative cumulative frequency (RCF) of correlation coefficients in Figs. S4F to I: $RCF_c(x) := \frac{1}{T}\sum_{i=1}^{T} \mathbf{1}_{[-1,x]}(c_i)$, where $\mathbf{1}_A(y)$ is the indicator function of a given set A: $\mathbf{1}_A(y) = 1$ if $y \in A$, and $\mathbf{1}_A(y) = 0$ otherwise.

We define the random variables $\mathbf{w}'$, $\mathbf{c}'$ and RCF'_c. $\mathbf{w}'$ is generated by shuffling the entries of a connectivity vector $\widehat{\mathbf{w}}$:

$$w'_i := w_{\sigma(i)} \quad \text{[S2]}$$

$$\mathbf{c}' := \widehat{\mathbf{w}}'^{\top}\widehat{\mathbf{X}} \quad \text{[S3]}$$

$$RCF'_c(x) := \frac{1}{T}\sum_{i=1}^{T} \mathbf{1}_{[-1,x]}(c'_i) \quad \text{[S4]}$$

Where $\sigma(i)$ is a random permutation operator. We define $\overline{RCF}'_c(x)$ (Figs. S4F to I, black line) as the mean $RCF'_c(x)$ arising from all RCFs that come from shuffled $\widehat{\mathbf{w}}$. Next, we define, the maximum negative deviation δ' (Figs. S4J to M) random variable as:

$$\delta' := \max_x \left[\overline{RCF}'_c(x) - RCF'_c(x)\right] \quad \text{[S5]}$$

Finally, we define p-value $= \Pr(\delta' \geq \delta_{true})$. The p-value is thus the proportion of RCFs generated with the random shuffling of entries of $\widehat{\mathbf{w}}$ that deviate from the mean RCF more than the true RCF.

Numerically, these calculations were done by binning the RCF function into 0.02 bins and generating 10000 instances of shuffled $\widehat{\mathbf{w}}$.

4. Number of aligned dimensions between the activity and connectivity subspaces (Fig. S7)

A. Results. To examine the alignment of the subspace spanned by the four $\mathbf{w}_{\mathrm{LNtype}}$'s and the one spanned by the top five PCA directions of $\{\mathbf{x}^{(t)}\}_{\mathrm{data}}$, we define a measure $0 \leq \Gamma \leq 4$, which approximately represents the number of aligned dimensions between these 2 subspaces and find $\Gamma \approx 2$. This value significantly deviates from the expected Γ from subspaces generated by 4 and 5 Gaussian random normal vectors in 21 dimensions ($p < 10^{-4}$) and subspaces generated from the 4 connectivity vectors with shuffled entries and the top 5 PCA directions ($p < 0.01$) (Fig. S7). Approximately 1 more dimension is significantly aligned between the 2 subspaces than expected by random, supporting the results of Fig. 2H, but there is no complete alignment between the connectivity $\{\mathbf{w}_{\mathrm{LNtype}}\}$ and the ORN activity principal subspace. Below we describe the rationale behind the measure Γ.

B. Methods. Given a Hilbert space of dimension D, we define Ω - a measure of dissimilarity between 2 subspaces $\mathbf{S}_A$ and $\mathbf{S}_B$ generated by the matrices of linearly independent K_A and K_B column vectors: $\mathbf{A} \in \mathbb{R}^{D\times K_A}$ and $\mathbf{B} \in \mathbb{R}^{D\times K_B}$:

$$\begin{aligned}
\Omega :=& \|\mathbf{P}_A - \mathbf{P}_B\|_F^2 && \text{[S6]}\\
=& \operatorname{Tr}\left[\mathbf{P}_A^2\right] + \operatorname{Tr}\left[\mathbf{P}_B^2\right] - 2\operatorname{Tr}\left[\mathbf{P}_A\mathbf{P}_B\right] && \text{[S7]}\\
=& \operatorname{Tr}\left[\mathbf{P}_A\right] + \operatorname{Tr}\left[\mathbf{P}_B\right] - 2\operatorname{Tr}\left[\mathbf{P}_A\mathbf{P}_B\right] && \text{[S8]}\\
=& \dim\left[\mathbf{S}_A\right] + \dim\left[\mathbf{S}_B\right] - 2\operatorname{Tr}\left[\mathbf{P}_A\mathbf{P}_B\right] && \text{[S9]}\\
=& K_A + K_B - 2\operatorname{Tr}\left[\mathbf{P}_A\mathbf{P}_B\right] && \text{[S10]}
\end{aligned}$$

Where $\mathbf{P}_A, \mathbf{P}_B \in \mathbb{R}^{D\times D}$ are the orthogonal projectors onto the subspaces S_A and S_B, respectively, F stands for the Frobenius norm, Tr is the matrix trace, and $K_X = \dim(\mathbf{S}_X)$ is the dimensionality of a subspace S_X. In the above equalities, we use the following properties of orthogonal projectors: $\mathbf{P}_A^2 = \mathbf{P}_A$, meaning that they are idempotent.

Any idempotent matrix has only eigenvalues 1 and 0 and has as many eigenvalues of value 1 as the number of dimensions it projects on. Thus the trace of a projector is its rank, i.e., the dimensionality of the space it projects on. We assume $K_A + K_B \leq D$. We have that $|K_A - K_B| \leq \Omega \leq K_A + K_B$. The projection matrix can be obtained thus $\mathbf{P}_A = \mathbf{A}(\mathbf{A}^\top\mathbf{A})^{-1}\mathbf{A}^\top$, or via QR factorization: $\mathbf{QR} = \mathbf{A}$, $\mathbf{P}_A = \mathbf{QQ}^\top$.

Intuitively, for two very similar subspaces, the projection $\mathbf{P}_A v$ of an arbitrary vector v onto S_A will be very similar to the projection $\mathbf{P}_B v$ vector v onto S_B, thus $\mathbf{P}_A v \approx \mathbf{P}_B v$ and Ω will be small. Conversely, if the subspaces are very different, the projections $\mathbf{P}_A v$ and $\mathbf{P}_B v$ will also be different and Ω will be large.

We now define the more intuitive measure Γ:

$$\Gamma := (K_A + K_B - \Omega)/2 \tag{S11}$$

which is a proxy of the number of aligned dimensions in the two subspaces. Indeed $0 \leq \Gamma \leq \min(K_A, K_B)$. For 2 perpendicular subspaces, $\Gamma = 0$ and for 2 fully aligned subspaces $\Gamma = \min(K_A, K_B)$.

In the main text, we refer to the subspaces spanned by the following matrices: $\mathbf{A} = [\mathbf{w}_{\mathrm{BT}}, \mathbf{w}_{\mathrm{BD}}, \mathbf{w}_{\mathrm{KS}}, \mathbf{w}_{\mathrm{P0}}]$ and $\mathbf{B}$ is the matrix with the top 5 PCA loading vectors of $\{\mathbf{x}^{(t)}\}$ as columns, $K_A = \dim[\mathbf{S}_A] = 4$, $K_B = \dim[\mathbf{S}_B] = 5$ and $D = 21$.

5. Hierarchical clustering for plotting Fig. 3D

To plot Fig. 3D, we ordered the correlation matrix using hierarchical clustering. For that we used the Python function scipy.cluster.hierarchy.linkage with the options method='average', optimal_ordering=True (3).

6. Optimization problem (Eq. (4) in the main text)

A. Description. We postulate the following minimax optimization problem:

$$\min_{\mathbf{Y}} \max_{\mathbf{Z}} \frac{1}{T^2}\left(\frac{T}{2}\|\mathbf{X}-\mathbf{Y}\|_F^2 - \frac{\rho^2}{4u^2}\left\|\mathbf{Y}^\top\mathbf{Y} - \frac{\gamma^2}{\rho^2}\mathbf{Z}^\top\mathbf{Z}\right\|_F^2 + \frac{\rho^2}{4u^2}\left\|\mathbf{Y}^\top\mathbf{Y}\right\|_F^2\right) \tag{S12}$$

Where $\|\cdot\|_F^2$ is the square of the matrix Frobenius (Euclidean) norm, $\mathbf{X}, \mathbf{Y} \in \mathbb{R}^{D\times T}$, $\mathbf{Z} \in \mathbb{R}^{K\times T}$ with D the number of ORNs (21 for this olfactory circuit), K the number of LNs, T the number of data (sample) points, ρ and γ positive unitless parameters, u a unit with the physical dimension as $\mathbf{X}$, $\mathbf{Y}$, and $\mathbf{Z}$ (e.g., spikes $\cdot\, s^{-1}$) (dropped for simplicity in the main text). $\mathbf{X}$, $\mathbf{Y}$, and $\mathbf{Z}$ represent the activity of ORN somas, ORN axons, and LNs, respectively. We can interpret $\mathbf{X}$ as all the discretized activity of ORNs up to a certain point in their lifetime. We set $\gamma = 1$ in the main text, as this parameter does not alter the computation, and only linearly scales synaptic weights and $\mathbf{Z}$. We have kept it in all derivations here in the supplement.

The optimization problem Eq. (S12) leads to the Linear Circuit (LC) model. Adding the nonnegativity constraints on $\mathbf{Y}$ and $\mathbf{Z}$ ($\mathbf{Y} \geq 0$ and $\mathbf{Z} \geq 0$) leads to the NonNegative Circuit (NNC) model.

We expand the optimization function in Eq. (S12). Using the property that $\|\mathbf{X}\|_F^2 = \mathrm{Tr}[\mathbf{X}^\top\mathbf{X}]$ and $\mathrm{Tr}[\mathbf{A}+\mathbf{B}] = \mathrm{Tr}[\mathbf{A}] + \mathrm{Tr}[\mathbf{B}]$, where $\mathrm{Tr}[\cdot]$ is the matrix trace (sum of the diagonal elements of the matrix) we get:

$$\min_{\mathbf{Y}} \max_{\mathbf{Z}} \frac{1}{T^2}\mathrm{Tr}\left[\frac{T}{2}\mathbf{X}^\top\mathbf{X} - T\mathbf{X}^\top\mathbf{Y} + \frac{T}{2}\mathbf{Y}^\top\mathbf{Y} - \frac{\rho^2}{4u^2}\mathbf{Y}^\top\mathbf{Y}\mathbf{Y}^\top\mathbf{Y} + \frac{\gamma^2}{2u^2}\mathbf{Y}^\top\mathbf{Y}\mathbf{Z}^\top\mathbf{Z} - \frac{\gamma^4}{4u^2\rho^2}\mathbf{Z}^\top\mathbf{Z}\mathbf{Z}^\top\mathbf{Z} + \frac{\rho^2}{4u^2}\mathbf{Y}^\top\mathbf{Y}\mathbf{Y}^\top\mathbf{Y}\right] \tag{S13}$$

$$\iff \min_{\mathbf{Y}} \max_{\mathbf{Z}} \frac{1}{T^2}\mathrm{Tr}\left[-T\mathbf{X}^\top\mathbf{Y} + \frac{T}{2}\mathbf{Y}^\top\mathbf{Y} + \frac{\gamma^2}{2u^2}\mathbf{Y}^\top\mathbf{Y}\mathbf{Z}^\top\mathbf{Z} - \frac{\gamma^4}{4u^2\rho^2}\mathbf{Z}^\top\mathbf{Z}\mathbf{Z}^\top\mathbf{Z}\right] \tag{S14}$$

Where we dropped the $\mathbf{X}^\top\mathbf{X}$ term because it does not influence the solution of the optimization problem.

B. Equivalence of scaling $\mathbf{X}$ and ρ. Here, we show that scaling $\mathbf{X}$ is equivalent to scaling ρ in the optimization. It is easy to see that the transformation $\mathbf{X} \to a\mathbf{X}$, $\mathbf{Y} \to a\mathbf{Y}$ and $\rho \to \rho/a$ (for $a \neq 0$) only scales the objective function, which does not affect the optima of the optimization, i.e., this transformation is a symmetry of the optimization. Indeed:

$$\min_{\mathbf{Y}} \max_{\mathbf{Z}} \frac{1}{T^2}\mathrm{Tr}\left[-T\mathbf{X}^\top\mathbf{Y} + \frac{T}{2}\mathbf{Y}^\top\mathbf{Y} + \frac{\gamma^2}{2u^2}\mathbf{Y}^\top\mathbf{Y}\mathbf{Z}^\top\mathbf{Z} - \frac{\gamma^4}{4u^2\rho^2}\mathbf{Z}^\top\mathbf{Z}\mathbf{Z}^\top\mathbf{Z}\right] \tag{S15}$$

$$\iff \min_{\mathbf{Y}} \max_{\mathbf{Z}} \frac{1}{T^2}\mathrm{Tr}\left[-Ta^2\mathbf{X}^\top\mathbf{Y} + \frac{T}{2}a^2\mathbf{Y}^\top\mathbf{Y} + \frac{a^2\gamma^2}{2u^2}\mathbf{Y}^\top\mathbf{Y}\mathbf{Z}^\top\mathbf{Z} - \frac{a^2\gamma^4}{4u^2\rho^2}\mathbf{Z}^\top\mathbf{Z}\mathbf{Z}^\top\mathbf{Z}\right] \tag{S16}$$

Let us explore the consequence of this symmetry. The solution $\mathbf{Y}^*$ of the optimization is a function of $\mathbf{X}$ and ρ, thus we can define a function f such that: $\mathbf{Y}^* = f(\mathbf{X}, \rho)$:

$$\mathbf{Y}^* = f(\mathbf{X}, \rho) := \arg\min_{\mathbf{Y}} \max_{\mathbf{Z}} \frac{1}{T^2} \operatorname{Tr}\left[-T\mathbf{X}^\top\mathbf{Y} + \frac{T}{2}\mathbf{Y}^\top\mathbf{Y} + \frac{\gamma^2}{2u^2}\mathbf{Y}^\top\mathbf{Y}\mathbf{Z}^\top\mathbf{Z} - \frac{\gamma^4}{4u^2\rho^2}\mathbf{Z}^\top\mathbf{Z}\mathbf{Z}^\top\mathbf{Z}\right] \tag{S17}$$

The symmetry implies:

$$\mathbf{Y}^* = f(\mathbf{X}, \rho) \Longleftrightarrow a\mathbf{Y}^* = f(a\mathbf{X}, \rho/a) \tag{S18}$$

As a consequence, first, we have that:

$$\mathbf{Y}^* = f(\mathbf{X}, \rho) = \frac{1}{a} f(a\mathbf{X}, \rho/a) \tag{S19}$$

Second, let's define $b = a^{-1}$ and $\mathbf{X}' = a\mathbf{X} = \mathbf{X}/b$. Then, by substituting the variables we get:

$$f(\mathbf{X}, \rho) = \frac{1}{a} f(a\mathbf{X}, \rho/a) \tag{S20}$$

$$\Longleftrightarrow f(b\mathbf{X}', \rho) = b f(\mathbf{X}', b\rho) \tag{S21}$$

$$\tag{S22}$$

In summary, we have the following properties:

$$f(\mathbf{X}, \rho) = \frac{1}{a} f(a\mathbf{X}, \rho/a) \quad \text{and} \quad f(a\mathbf{X}, \rho) = a f(\mathbf{X}, a\rho) \tag{S23}$$

This means performing an optimization with an input $a\mathbf{X}$, is equivalent to doing the optimization with input $\mathbf{X}$ and parameter $a\rho$, and finally multiplying the obtained $\mathbf{Y}^*$ by a.

It is worth noting though, that for a circuit with fixed $\mathbf{W}$ and $\mathbf{M}$, scaling an input $\mathbf{x}$ by a factor a, simply scales the output $\mathbf{y}$ by the same factor a, since it is a linear transformation, at least for the circuit without the nonnegative constraints.

C. Equivalence of scaling $\mathbf{Z}$ and γ. We can see that the transformation $\mathbf{Z} \to a\mathbf{Z}$ and $\gamma \to \gamma/a$ does not change the objective function, i.e., this transformation is a symmetry of the optimization. Indeed:

$$\min_{\mathbf{Y}} \max_{\mathbf{Z}} \frac{1}{T^2} \left(\frac{T}{2} \|\mathbf{X} - \mathbf{Y}\|_F^2 - \frac{\rho^2}{4u^2} \left\| \mathbf{Y}^\top\mathbf{Y} - \frac{\gamma^2}{\rho^2}\mathbf{Z}^\top\mathbf{Z} \right\|_F^2 + \frac{\rho^2}{4u^2} \left\| \mathbf{Y}^\top\mathbf{Y} \right\|_F^2 \right) \tag{S24}$$

$$\Longleftrightarrow \min_{\mathbf{Y}} \max_{\mathbf{Z}} \frac{1}{T^2} \left(\frac{T}{2} \|\mathbf{X} - \mathbf{Y}\|_F^2 - \frac{\rho^2}{4u^2} \left\| \mathbf{Y}^\top\mathbf{Y} - \frac{\gamma^2}{a^2\rho^2}(a\mathbf{Z}^\top)(a\mathbf{Z}) \right\|_F^2 + \frac{\rho^2}{4u^2} \left\| \mathbf{Y}^\top\mathbf{Y} \right\|_F^2 \right) \tag{S25}$$

7. Offline solution/computation of the optimization problem

A. Solution for the LC (Eq. (3a), Eq. (3b), Eq. (3c) in the main text). Here we describe the solution of the optimization problem Eq. (S14), without any constraints on $\mathbf{Y}$ and $\mathbf{Z}$. As we show below, this solution can also be found by a circuit model, with the same architecture as the olfactory circuit under study. The situation without constraints on $\mathbf{Y}$ and $\mathbf{Z}$ corresponds to the Linear Circuit (LC) model. Understanding the solution of the optimization problem allows us to understand the computation performed by such a circuit model.

We use the singular value decomposition (SVD) for $\mathbf{X}$, $\mathbf{Y}$, and $\mathbf{Z}$: $\mathbf{X} = \mathbf{U}_X\tilde{\mathbf{S}}_X\mathbf{V}_X^\top$, $\mathbf{Y} = \mathbf{U}_Y\tilde{\mathbf{S}}_Y\mathbf{V}_Y^\top$, $\mathbf{Z} = \mathbf{U}_Z\tilde{\mathbf{S}}_Z\mathbf{V}_Z^\top$, with the following convention: $\mathbf{U}_X, \mathbf{U}_Y \in \mathbb{R}^{D\times D}$, $\mathbf{U}_Z \in \mathbb{R}^{K\times K}$, $\mathbf{V}_X, \mathbf{V}_Y, \mathbf{V}_Z \in \mathbb{R}^{T\times T}$, $\tilde{\mathbf{S}}_X, \tilde{\mathbf{S}}_Y \in \mathbb{R}^{D\times T}$, $\tilde{\mathbf{S}}_Z \in \mathbb{R}^{K\times T}$, $\tilde{\mathbf{S}}_X, \tilde{\mathbf{S}}_Y, \tilde{\mathbf{S}}_Z$ only have values on the diagonal. We call $\mathbf{S} \in \mathbb{R}^{T\times T}$ the diagonal square matrix corresponding to the rectangular matrix $\tilde{\mathbf{S}}$, with padded zeros. Only the first D columns in $\mathbf{V}_X$ and $\mathbf{V}_Y$ and the first K in $\mathbf{V}_Z$ contain relevant information about $\mathbf{X}$, $\mathbf{Y}$, and $\mathbf{Z}$, respectively. The left singular vectors $\mathbf{U}_X$, $\mathbf{U}_Y$, and $\mathbf{U}_Z$ are also the principal directions of the uncentered PCA of $\mathbf{X}$, $\mathbf{Y}$, and $\mathbf{Z}$, respectively. Whereas the values on the diagonal of $\tilde{\mathbf{S}}_X$, $\tilde{\mathbf{S}}_Y$, and $\tilde{\mathbf{S}}_Z$ are the square root of the variances of the corresponding uncentered PCA directions. For b - a variable of the optimization problem, we call b^* an optimal value (solution). In the results section of the main text, we dropped the star symbol *.

In the following we prove that $\mathbf{Y}^*$ and $\mathbf{Z}^*$, the optima in $\mathbf{Y}$ and $\mathbf{Z}$ in the optimization problem Eq. (S14), are given by:

$$\mathbf{Y}^* = \mathbf{U}_X \tilde{\mathbf{S}}_Y^* \mathbf{V}_X^\top = \mathbf{U}_X \tilde{\mathbf{S}}_Y^* \tilde{\mathbf{S}}_X^+ \mathbf{U}_X^\top \mathbf{X} \tag{S26}$$

$$\mathbf{Z}^* = \rho/\gamma \mathbf{U}_Z^* \tilde{\mathbf{S}}_{Y|K}^* \mathbf{V}_X^\top = \rho/\gamma \mathbf{U}_Z^* \tilde{\mathbf{S}}_{Y|K}^* \tilde{\mathbf{S}}_X^+ \mathbf{U}_X^\top \mathbf{X} \tag{S27}$$

$$\text{with} \quad \begin{cases} s_{Y,i}^* \left(1 + \dfrac{\rho^2}{u^2 T} s_{Y,i}^{*2}\right) = s_{X,i} & 1 \le i \le K \quad \text{[S28a]} \\ s_{Y,i}^* = s_{X,i} & K+1 \le i \le D \quad \text{[S28b]} \\ \mathbf{U}_Z^*\text{: a degree of freedom} & \quad \text{[S28c]} \end{cases}$$

where $\mathbf{A}^+$ the Moore-Penrose pseudo-inverse of $\mathbf{A}$ and $\tilde{\mathbf{S}}_{Y|K}^* \in \mathbb{R}^{K\times T}$ is the matrix with the first K row of $\tilde{\mathbf{S}}_Y^*$. These equations lead to relations Eq. (3a), Eq. (3b), Eq. (3c) in the main text, where the relations are written in terms of PCA variances rather than singular values. The relationship between a singular value s and the corresponding PCA variance σ^2 is $s^2/T = \sigma^2$.

In other words, writing $\mathbf{Y}^* = \mathbf{F}\mathbf{X}$, we have that $\mathbf{F} = \mathbf{F}^\top = \mathbf{U}_X \tilde{\mathbf{S}}_Y^* \tilde{\mathbf{S}}_X^+ \mathbf{U}_X^\top$, $\mathbf{S}_Y^* \mathbf{S}_X^+$ being a diagonal matrix. This signifies that the linear transformation $\mathbf{F}$ does not perform any rotation of the input.

In particular, we find that $\mathbf{U}_Y^* = \mathbf{U}_X$, meaning that the ORN soma input $\mathbf{X}$ and the ORN axon output $\mathbf{Y}^*$ have the same left singular vectors (although the order can be different). The left singular vectors correspond to the directions of uncentered PCA. Also, we find that $\mathbf{V}_Y^* = \mathbf{V}_Z^* = \mathbf{V}_X$, meaning that the right singular vectors of $\mathbf{X}$, $\mathbf{Y}^*$ and $\mathbf{Z}^*$ are the same (although, again, their order can be different). The ith right singular vector corresponds to the neural activity in the ith singular (or uncentered PCA) direction. The equality between the right singular directions in $\mathbf{X}$ and $\mathbf{Y}^*$ means that the neural activity in a singular direction $\mathbf{u}_{X,i}$ at the level of the ORN somas and at the level of the ORN axons is the same up to a multiplication factor. Similarly, the neural activity in the direction $\mathbf{u}_{Z,i}$ at the level of LNs is proportional to the activity in the direction $\mathbf{u}_{X,i}$ at the level of ORN somas or axons. Thus, when looking at the neural activity in the directions of a left singular vector i (PCA direction i) at the level of ORN soma, axons, and LNs, the activity is the same up to a multiplication factor. The multiplication factor is set by the ratios between the corresponding singular values (or PCA variances).

This explicit expressions for s_Y^* and s_Z^* are:

$$s_Y^* = \frac{1}{\rho}\left(\frac{\sqrt{12T^3u^6 + 81T^2u^4\rho^2 s_X^2} + 9Tu^2\rho s_X}{18}\right)^{\frac{1}{3}} - \frac{1}{\rho}\left(\frac{\frac{2}{3}T^3u^6}{\sqrt{12T^3u^6 + 81T^2u^4\rho^2 s_X^2} + 9Tu^2\rho s_X}\right)^{\frac{1}{3}} \tag{S29}$$
$$s_Z^* = \frac{\rho}{\gamma} s_Y$$

The behavior of s_Y^* is such:

$$s_Y^* \approx \begin{cases} s_X & s_X \ll \frac{\sqrt{T}u}{\rho} \quad \text{[S30a]} \\ \sqrt[3]{\dfrac{Tu^2}{\rho^2} s_X} & s_X \gg \frac{\sqrt{T}u}{\rho} \quad \text{[S30b]} \end{cases}$$

Note that because $\mathbf{Z}$ only appears as $\mathbf{Z}^\top\mathbf{Z}$ in the optimization problem Eq. (S14), $\mathbf{U}_Z^*$ is a degree of freedom of the optimization. Thus, for $\{\mathbf{Y}^*, \mathbf{Z}^*, \mathbf{W}^*, \mathbf{M}^*\}$ a solution of the optimization, $\{\mathbf{Y}^*, \mathbf{Q}\mathbf{Z}^*, \mathbf{W}^*\mathbf{Q}^\top, \mathbf{Q}\mathbf{M}^*\mathbf{Q}^\top\}$ is a solution as well, where $\mathbf{Q} \in \mathbb{R}^{K\times K}$ is an orthogonal matrix. Consequently, there is a manifold of $\mathbf{W}^*$, $\mathbf{M}^*$, and $\mathbf{Z}^*$ that satisfies the optimization for the LC.

B. Proof. For convenience, we copy here the optimization problem Eq. (S14):

$$\min_{\mathbf{Y}} \max_{\mathbf{Z}} \mathrm{OF}(\mathbf{Y}, \mathbf{Z}) \tag{S31}$$

Where the objective function $\mathrm{OF}(\mathbf{Y}, \mathbf{Z})$ is:

$$\mathrm{OF}(\mathbf{Y}, \mathbf{Z}) := \frac{1}{T^2} \mathrm{Tr}\left[-T\mathbf{X}^\top\mathbf{Y} + \frac{T}{2}\mathbf{Y}^\top\mathbf{Y} + \frac{\gamma^2}{2u^2}\mathbf{Y}^\top\mathbf{Y}\mathbf{Z}^\top\mathbf{Z} - \frac{\gamma^4}{4u^2\rho^2}\mathbf{Z}^\top\mathbf{Z}\mathbf{Z}^\top\mathbf{Z}\right] \tag{S32}$$

We first find the optimum in $\mathbf{Y}$ of this objective function by taking the partial derivative of $\mathrm{OF}(\mathbf{Y}, \mathbf{Z})$ with respect to $\mathbf{Y}$ and equating it to $\mathbf{0}$. We thus obtain the expression for $\mathbf{Y}$:

$$\mathbf{Y} = \mathbf{X}\left(\mathbf{I}_T + \frac{\gamma^2}{Tu^2}\mathbf{Z}^\top\mathbf{Z}\right)^{-1} \tag{S33}$$

where $\mathbf{I}_T$ is the identity matrix of dimension T. What is the meaning of this equality? Since this equality is obtained by finding the optima in $\mathbf{Y}$, this equation gives the expression for the axonal output $\mathbf{Y}$ for an arbitrary input $\mathbf{X}$ and LN activity $\mathbf{Z}$. To intuitively understand this expression, we imagine that $\mathbf{Z}$ is of dimension $1 \times T$, which corresponds to just 1 LN. We use the SVD expansion of $\mathbf{Z}$:

$$\begin{aligned}\mathbf{Z} &= [z^{(1)}, ..., z^{(T)}] && \text{[S34]}\\ &= \mathbf{U}_Z\tilde{\mathbf{S}}_Z\mathbf{V}_Z^\top && \text{[S35]}\\ &= 1 \times [s_{Z,1}, 0, ..., 0] \times \mathbf{V}_Z^\top && \text{[S36]}\end{aligned}$$

Where $\mathbf{U}_Z = 1$ because it is a orthogonal matrix of dimension 1, $\tilde{\mathbf{S}}_Z$ is of dimension $1 \times T$, $\mathbf{V}_Z$ is of dimension $T \times T$ and we have that the first column of $\mathbf{V}_Z$ is $[z^{(1)}, ..., z^{(T)}]^\top / s_{Z,1}$, and $s_{Z,1}$ is the norm of $\mathbf{Z}$, i.e., $s_{Z,1} = (\sum_{t=1}^T (z^{(t)})^2)^{1/2}$. We now put this expression of $\mathbf{Z}$ into Eq. (S33):

$$\begin{aligned}\mathbf{Y} &= \mathbf{X}\left(\mathbf{I}_T + \frac{\gamma^2}{Tu^2}\mathbf{Z}^\top\mathbf{Z}\right)^{-1} && \text{[S37]}\\ &= \mathbf{X}\left(\mathbf{I}_T + \frac{\gamma^2}{Tu^2}\mathbf{V}_Z\mathbf{S}_Z^2\mathbf{V}_Z^\top\right)^{-1} && \text{[S38]}\\ &= \mathbf{X}\mathbf{V}_Z\left(\mathbf{I}_T + \frac{\gamma^2}{Tu^2}\mathbf{S}_Z^2\right)^{-1}\mathbf{V}_Z^\top && \text{[S39]}\end{aligned}$$

where $(\mathbf{I}_T + \gamma^2/(Tu^2)\mathbf{S}_Z^2)^{-1}$ is a $T \times T$ diagonal matrix, where all the diagonal elements are 1, apart from the first one which is: $(1 + \gamma^2/(Tu^2)s_{Z,1}^2)^{-1}$. This implies that the activity in $\mathbf{Y}$ is the same as the activity in $\mathbf{X}$, apart from the directions of the K first right singular vectors of $\mathbf{Z}$. In those directions it is diminished by the factors $(1 + \gamma^2/(Tu^2)s_{Z,k}^2)^{-1}$. In other words, the directions of activity (in terms of right singular vectors) that are the most dampened in $\mathbf{Y}$ in comparison to $\mathbf{X}$, are those which are the most aligned/correlated with the activity in $\mathbf{Z}$.

Next, we replace the solution Eq. (S33) for $\mathbf{Y}$ into the original optimization problem Eq. (S14), obtaining the equivalent optimization problem:

$$\min_{\mathbf{Z}} \frac{1}{T^2}\,\mathrm{Tr}\left[\frac{T}{2}\mathbf{X}^\top\mathbf{X}\left(\mathbf{I}_T + \frac{\gamma^2}{Tu^2}\mathbf{Z}^\top\mathbf{Z}\right)^{-1} + \frac{\gamma^4}{4u^2\rho^2}\mathbf{Z}^\top\mathbf{Z}\mathbf{Z}^\top\mathbf{Z}\right] \tag{S40}$$

Next we replace $\mathbf{X}$ and $\mathbf{Z}$ by their SVD, use the property of the trace $\mathrm{Tr}(\mathbf{AB}) = \mathrm{Tr}(\mathbf{BA})$ and the property of orthogonal matrices $\mathbf{U}\mathbf{U}^\top = \mathbf{U}^\top\mathbf{U} = \mathbf{I}$:

$$\begin{aligned}&\min_{\mathbf{Z}} \frac{1}{T^2}\,\mathrm{Tr}\left[\frac{T}{2}\mathbf{V}_X\mathbf{S}_X^2\mathbf{V}_X^\top\left(\mathbf{I}_T + \frac{\gamma^2}{Tu^2}\mathbf{V}_Z\mathbf{S}_Z^2\mathbf{V}_Z^\top\right)^{-1} + \frac{\gamma^4}{4u^2\rho^2}\mathbf{S}_Z^4\right] && \text{[S41]}\\ \Longleftrightarrow &\min_{\mathbf{Z}} \mathrm{Tr}\left[\frac{1}{2T}\mathbf{V}_X\mathbf{S}_X^2\mathbf{V}_X^\top\left(\frac{\mathbf{V}_Z(Tu^2\mathbf{I}_T + \gamma^2\mathbf{S}_Z^2)\mathbf{V}_Z^\top}{Tu^2}\right)^{-1} + \frac{\gamma^4}{4T^2u^2\rho^2}\mathbf{S}_Z^4\right] && \text{[S42]}\\ \Longleftrightarrow &\min_{\mathbf{Z}} \mathrm{Tr}\left[\frac{u^2}{2}\mathbf{V}_X\mathbf{S}_X^2\mathbf{V}_X^\top\mathbf{V}_Z(Tu^2\mathbf{I}_T + \gamma^2\mathbf{S}_Z^2)^{-1}\mathbf{V}_Z^\top + \frac{\gamma^4}{4T^2u^2\rho^2}\mathbf{S}_Z^4\right] && \text{[S43]}\\ \Longleftrightarrow &\min_{\mathbf{Z}} \mathrm{Tr}\left[\frac{1}{2}\mathbf{V}_X\mathbf{S}_X^2\mathbf{V}_X^\top\mathbf{V}_Z(Tu^2\mathbf{I}_T + \gamma^2\mathbf{S}_Z^2)^{-1}\mathbf{V}_Z^\top + \frac{\gamma^4}{4T^2u^4\rho^2}\mathbf{S}_Z^4\right] && \text{[S44]}\end{aligned}$$

Where, for the last equivalence we used the fact that multiplying the objective function by a constant (here u^{-2}) does not alter the optimization problem. Since $\mathbf{U}_Z$ does not appear in the minimization, it is a free parameter, i.e., it can be any orthogonal matrix. For fixed $\mathbf{S}_Z$, only the first term in the trace needs to be minimized. One can show that the optimal $\mathbf{V}_Z^*$ is $\mathbf{V}_Z^* = \mathbf{V}_X$: based on von Neumann trace inequality, we know that $\mathrm{Tr}[\mathbf{AB}] \geq \sum_i^N a_i b_{N-i+1}$ where

a_i and b_i are the ordered singular values of $\mathbf{A}$ and $\mathbf{B}$, respectively. Thus, choosing $\mathbf{V}_Z = \mathbf{V}_X$ will give us the lower bound of that inequality. Indeed:

$$\begin{aligned} &\operatorname{Tr}\left[\mathbf{V}_X\mathbf{S}_X^2\mathbf{V}_X^\top\mathbf{V}_Z(Tu^2\mathbf{I}_T+\gamma^2\mathbf{S}_Z^2)^{-1}\mathbf{V}_Z^\top\right] \\ &=\operatorname{Tr}\left[\mathbf{S}_X^2(Tu^2\mathbf{I}_T+\gamma^2\mathbf{S}_Z^2)^{-1}\right] \\ &=\sum_i^T s_{X,i}^2\frac{1}{Tu^2+\gamma^2 s_{Z,i}^2} \end{aligned} \tag{S45}$$

Where $s_{X,i}$ and $s_{Z,i}$ are the values on the diagonal of $\mathbf{S}_X$ and $\mathbf{S}_Z$, respectively. Thus, the highest singular values of $\mathbf{V}_X\mathbf{S}_X^2\mathbf{V}_X^\top$ match the lowest singular values of $\mathbf{V}_Z\left(Tu^2\mathbf{I}_T+\gamma^2\mathbf{S}_Z^2\right)^{-1}\mathbf{V}_Z^\top$, giving us the lower bound of the von Neumann inequality. The optimization problem Eq. (S40) can now be simplified to:

$$\min_{\{s_{Z,i}\}}\operatorname{OF}(\{s_{Z,i}\}) = \min_{\{s_{Z,i}\}}\sum_i^T\left(\frac{1}{2}s_{X,i}^2\frac{1}{Tu^2+\gamma^2 s_{Z,i}^2}+\frac{\gamma^4}{4T^2u^4\rho^2}s_{Z,i}^4\right) \tag{S46}$$

Each $s_{Z,i}$ can be minimized independently. By construction of SVD, we already have that $s_{Z,i}=0$ for $i>K$. We thus consider $1\le i\le K$. To simplify notation, we drop the index i. We take the derivative of $\operatorname{OF}(\{s_{Z,i}\})$ in Eq. (S46) with respect to $s_{Z,i}$ and equate it to 0 (we drop the index i for convenience of notation):

$$\frac{\partial\,\operatorname{OF}}{\partial s_Z}=0 \tag{S47}$$

$$-\frac{\gamma^2}{(Tu^2+\gamma^2 s_Z^2)^2}s_X^2 s_Z+\frac{\gamma^4}{T^2u^4\rho^2}s_Z^3=0 \tag{S48}$$

$$s_X^2=\frac{\gamma^2}{\rho^2}\frac{(Tu^2+\gamma^2 s_Z^2)^2}{T^2u^4}s_Z^2 \tag{S49}$$

This leads to, considering that singular values are positive:

$$s_X=\frac{\gamma}{\rho}s_Z^*\left(1+\frac{\gamma^2}{Tu^2}s_Z^{*2}\right) \tag{S50}$$

We can now use the obtained solution for $\mathbf{Z}$ to find the solution for $\mathbf{Y}$. We replace $\mathbf{X}$ and $\mathbf{Z}$ by their SVD in relation Eq. (S33) and use that $\mathbf{V}_Z^*=\mathbf{V}_X$:

$$\mathbf{Y}^*=\mathbf{U}_Y^*\tilde{\mathbf{S}}_Y^*\mathbf{V}_Y^{*\top}=\mathbf{X}\left(\mathbf{I}_T+\frac{\gamma^2}{Tu^2}\mathbf{Z}^{*\top}\mathbf{Z}^*\right)^{-1} \tag{S51}$$

$$=\mathbf{U}_X\tilde{\mathbf{S}}_X\mathbf{V}_X^\top\left(\mathbf{I}_T+\frac{\gamma^2}{Tu^2}\mathbf{V}_X\mathbf{S}_Z^{*2}\mathbf{V}_X^\top\right)^{-1} \tag{S52}$$

$$=\mathbf{U}_X\tilde{\mathbf{S}}_X\mathbf{V}_X^\top\mathbf{V}_X\left(\mathbf{I}_T+\frac{\gamma^2}{Tu^2}\mathbf{S}_Z^{*2}\right)^{-1}\mathbf{V}_X^\top \tag{S53}$$

$$\mathbf{U}_Y^*\tilde{\mathbf{S}}_Y^*\mathbf{V}_Y^{*\top}=\mathbf{U}_X\tilde{\mathbf{S}}_X\left(\mathbf{I}_T+\frac{\gamma^2}{Tu^2}\mathbf{S}_Z^{*2}\right)^{-1}\mathbf{V}_X^\top \tag{S54}$$

Although the right-hand side of Eq. (S54) has the form of [orthogonal matrix] × [diagonal matrix] × [orthogonal matrix], it is not strictly the normal SVD expression, because the values on the diagonal of $\tilde{\mathbf{S}}_X(\mathbf{I}_T+\gamma^2/(Tu^2)\mathbf{S}_Z^{*2})^{-1}$ are not necessarily in decreasing order. Equating the terms on the left and right sides we obtain $\mathbf{U}_Y^*=\mathbf{U}_X$, $\mathbf{V}_Y^*=\mathbf{V}_X$ and $\tilde{\mathbf{S}}_Y^*=\tilde{\mathbf{S}}_X(\mathbf{I}_T+\gamma^2/(Tu^2)\mathbf{S}_Z^{*2})^{-1}$. The last equality gives:

$$s_{Y,i}^*=s_{X,i}\left(1+\frac{\gamma^2}{Tu^2}s_{Z,i}^{*2}\right)^{-1} \tag{S55}$$

Thus, for $i>K$, we have $s_{Y,i}^*=s_{X,i}$ (since $s_{Z,i}=0$), whereas for $i\le K$: $s_{Y,i}^*=\frac{\gamma}{\rho}s_{Z,i}^*$ (using relation Eq. (S50) to replace s_X). The relation analogous to Eq. (S50) is:

$$s_X=s_Y^*\left(1+\frac{\rho^2}{Tu^2}s_Y^{*2}\right) \tag{S56}$$

Note that the resulting decomposition of $\mathbf{Y}$ with $\mathbf{Y} = \mathbf{U}_X \tilde{\mathbf{S}}_Y \mathbf{V}_X$ is equal to the usual SVD decomposition of $\mathbf{Y}$, up to the order of the singular values and singular directions. In summary, for $i \leq K$:

$$s_{X,i} = \frac{\gamma}{\rho} s^*_{Z,i} \left(1 + \frac{\gamma^2}{Tu^2} s^{*2}_{Z,i}\right) \quad \text{[S57]}$$

$$s_{X,i} = s^*_{Y,i} \left(1 + \frac{\rho^2}{Tu^2} s^{*2}_{Y,i}\right) \quad \text{[S58]}$$

$$s^*_{Y,i} = \frac{\gamma}{\rho} s^*_{Z,i} \quad \text{[S59]}$$

and for $i > K$:

$$s^*_{Y,i} = s_{X,i} \quad \text{[S60]}$$

$$s^*_{Z,i} = 0 \quad \text{[S61]}$$

This ends the derivation.

C. Computation in LNs and relationship between the NNC and SNMF. To understand the computation at the level of LNs, we consider the optimization problem from the aspect of $\mathbf{Z}$, which represents LN activity. We copy here the original optimization problem Eq. (S12), while dropping the $1/T^2$ factor in front:

$$\min_{\mathbf{Y}} \max_{\mathbf{Z}} \left(\frac{T}{2} \|\mathbf{X} - \mathbf{Y}\|_F^2 - \frac{\rho^2}{4u^2} \left\|\mathbf{Y}^\top \mathbf{Y} - \frac{\gamma^2}{\rho^2} \mathbf{Z}^\top \mathbf{Z}\right\|_F^2 + \frac{\rho^2}{4u^2} \left\|\mathbf{Y}^\top \mathbf{Y}\right\|_F^2 \right) \quad \text{[S62]}$$

We can isolate the maximization over $\mathbf{Z}$:

$$\min_{\mathbf{Y}} \left(\frac{T}{2} \|\mathbf{X} - \mathbf{Y}\|_F^2 + \frac{\rho^2}{4u^2} \left\|\mathbf{Y}^\top \mathbf{Y}\right\|_F^2 + \max_{\mathbf{Z}} \left(-\frac{\rho^2}{4u^2} \left\|\mathbf{Y}^\top \mathbf{Y} - \frac{\gamma^2}{\rho^2} \mathbf{Z}^\top \mathbf{Z}\right\|_F^2 \right) \right) \quad \text{[S63]}$$

This means, that for a given $\mathbf{Y}$, the optimal $\mathbf{Z}$ can be found with the optimization problem:

$$\min_{\mathbf{Z}} \left\|\mathbf{Y}^\top \mathbf{Y} - \frac{\gamma^2}{\rho^2} \mathbf{Z}^\top \mathbf{Z}\right\|_F^2 \quad \text{[S64]}$$

Where we dropped the factor $\rho^2/(4u^2)$, which does influence the optimization and also changes the maximization to a minimization by changing the sign. This corresponds to the original, most simple similarity-matching optimization problem, that has been extensively studied (4, 5).

If we now add the nonnegativity constraint on $\mathbf{Z}$, the LN activity, one gets:

$$\min_{\mathbf{Z} \geq 0} \left\|\mathbf{Y}^\top \mathbf{Y} - \frac{\gamma^2}{\rho^2} \mathbf{Z}^\top \mathbf{Z}\right\|_F^2 \quad \text{[S65]}$$

Which is the Symmetric Nonnegative Matrix Factorization (SNMF) optimization problem (6). It has been shown that in this situation the activity in $\mathbf{Z}^*$ corresponds to the soft clustering memberships of clusters found in $\mathbf{Y}$, as seen in Figs. 5A, C, E, and 6A. SNMF corresponds to soft K-means clustering (6).

8. Online algorithm and its implementation by a neural circuit with ORN-LN architecture

Here we show that a neuron circuit model with the ORN-LN architecture (Fig. 1A) can solve the optimization problem Eq. (S14). We convey two messages. First, given an input $\mathbf{X}$, specific synaptic weights will allow the circuit to output the optimal $\mathbf{Y}^*$ and $\mathbf{Z}^*$. Second, the circuit is capable of finding on its own (i.e., in an unsupervised manner) the optimal synaptic weights to perform the computation of the optimization problem. For that, it is sufficient that synapses follow Hebbian synaptic plasticity rules. We derive Eq. (5), Eq. (6), Eq. (7), and Eq. (8) from the main text.

A. Circuit equations for the LC (Eq. (6) in the main text). We first derive the circuit equations for the LC (Eq. (6) in the main text). For convenience, we copy the optimization problem Eq. (S14) here:

$$\min_{\mathbf{Y}} \max_{\mathbf{Z}} \frac{1}{T^2} \operatorname{Tr}\left[-T\mathbf{X}^\top\mathbf{Y} + \frac{T}{2}\mathbf{Y}^\top\mathbf{Y} + \frac{\gamma^2}{2u^2}\mathbf{Y}^\top\mathbf{Y}\mathbf{Z}^\top\mathbf{Z} - \frac{\gamma^4}{4u^2\rho^2}\mathbf{Z}^\top\mathbf{Z}\mathbf{Z}^\top\mathbf{Z}\right] \tag{S66}$$

We then first introduce the unitless variables $\mathbf{W} \in \mathbb{R}^{D\times K}$ and $\mathbf{M} \in \mathbb{R}^{K\times K}$:

$$\mathbf{W} := \frac{1}{Tu^2}\mathbf{Y}\mathbf{Z}^\top, \quad \mathbf{M} := \frac{1}{Tu^2}\mathbf{Z}\mathbf{Z}^\top \tag{S67}$$

and perform the Hubbard-Stratonovich transform on the optimization problem Eq. (S14) (5):

$$\min_{\mathbf{Y}} \max_{\mathbf{Z}} \max_{\mathbf{W}} \min_{\mathbf{M}} \operatorname{OF}(\mathbf{Y}, \mathbf{Z}, \mathbf{W}, \mathbf{M}) \tag{S68}$$

where the objective function is now:

$$\operatorname{OF}(\mathbf{Y}, \mathbf{Z}, \mathbf{W}, \mathbf{M}) := \frac{1}{T}\operatorname{Tr}\left[-\mathbf{X}^\top\mathbf{Y} + \frac{1}{2}\mathbf{Y}^\top\mathbf{Y} + \gamma^2\mathbf{Y}^\top\mathbf{W}\mathbf{Z} - \frac{\gamma^4}{2\rho^2}\mathbf{Z}^\top\mathbf{M}\mathbf{Z}\right] - \frac{u^2\gamma^2}{2}\operatorname{Tr}\left[\mathbf{W}^\top\mathbf{W}\right] + \frac{u^2\gamma^4}{4\rho^2}\operatorname{Tr}\left[\mathbf{M}^\top\mathbf{M}\right] \tag{S69}$$

It can indeed be verified that the solution of the optimization in $\mathbf{W}$ of Eq. (S68) is $\mathbf{W} = \mathbf{Y}\mathbf{Z}^\top/(Tu^2)$ (by solving $\partial \operatorname{OF}(\mathbf{Y}, \mathbf{Z}, \mathbf{W}, \mathbf{M})/\partial\mathbf{W} = 0$) and that the solution of the optimization in $\mathbf{M}$ of Eq. (S68) is $\mathbf{M} = \mathbf{Z}\mathbf{Z}^\top/(Tu^2)$ (by solving $\partial \operatorname{OF}(\mathbf{Y}, \mathbf{Z}, \mathbf{W}, \mathbf{M})/\partial\mathbf{M} = 0$). Then, putting $\mathbf{W} = \mathbf{Y}\mathbf{Z}^\top/(Tu^2)$ and $\mathbf{M} = \mathbf{Z}\mathbf{Z}^\top/(Tu^2)$ into the optimization problem in Eq. (S68)-Eq. (S69), we get the original optimization problem Eq. (S14).

We then rewrite the objective function Eq. (S69) in vector notation, with each sample point written out separately:

$$\begin{aligned}\operatorname{OF}(\{\mathbf{y}^{(t)}\}, \{\mathbf{z}^{(t)}\}, \mathbf{W}, \mathbf{M}) := \frac{1}{T}\sum_{t=1}^{T}\left(-\mathbf{x}^{(t)\top}\mathbf{y}^{(t)} + \frac{1}{2}\mathbf{y}^{(t)\top}\mathbf{y}^{(t)} + \gamma^2\mathbf{y}^{(t)\top}\mathbf{W}\mathbf{z}^{(t)} - \frac{\gamma^4}{2\rho^2}\mathbf{z}^{(t)\top}\mathbf{M}\mathbf{z}^{(t)}\right)\\ - \frac{u^2\gamma^2}{2}\operatorname{Tr}\left[\mathbf{W}^\top\mathbf{W}\right] + \frac{u^2\gamma^4}{4\rho^2}\operatorname{Tr}\left[\mathbf{M}^\top\mathbf{M}\right]\end{aligned} \tag{S70}$$

Giving us the optimization problem:

$$\min_{\{\mathbf{y}^{(t)}\}} \max_{\{\mathbf{z}^{(t)}\}} \max_{\mathbf{W}} \min_{\mathbf{M}} \operatorname{OF}(\{\mathbf{y}^{(t)}\}, \{\mathbf{z}^{(t)}\}, \mathbf{W}, \mathbf{M}) \tag{S71}$$

Given the solution $\mathbf{Y}^*$ and $\mathbf{Z}^*$ to the optimization problem Eq. (S14), solutions for $\mathbf{W}$ and $\mathbf{M}$ are $\mathbf{W}^* = \mathbf{Y}^*\mathbf{Z}^{*\top}/(Tu^2)$ and $\mathbf{M}^* = \mathbf{Z}^*\mathbf{Z}^{*\top}/(Tu^2)$, which can be put in the optimization problem Eq. (S71), giving us the following new optimization problem:

$$\min_{\{\mathbf{y}^{(t)}\}} \max_{\{\mathbf{z}^{(t)}\}} \operatorname{OF}(\{\mathbf{y}^{(t)}\}, \{\mathbf{z}^{(t)}\}) \tag{S72}$$

where:

$$\begin{aligned}\operatorname{OF}(\{\mathbf{y}^{(t)}\}, \{\mathbf{z}^{(t)}\}) := \frac{1}{T}\sum_{t=1}^{T}\left(-\mathbf{x}^{(t)\top}\mathbf{y}^{(t)} + \frac{1}{2}\mathbf{y}^{(t)\top}\mathbf{y}^{(t)} + \gamma^2\mathbf{y}^{(t)\top}\mathbf{W}^*\mathbf{z}^{(t)} - \frac{\gamma^4}{2\rho^2}\mathbf{z}^{(t)\top}\mathbf{M}^*\mathbf{z}^{(t)}\right)\\ - \frac{u^2\gamma^2}{2}\operatorname{Tr}\left[\mathbf{W}^{*\top}\mathbf{W}^*\right] + \frac{u^2\gamma^4}{4\rho^2}\operatorname{Tr}\left[\mathbf{M}^{*\top}\mathbf{M}^*\right]\end{aligned} \tag{S73}$$

We can then perform the optimization of each $\mathbf{y}^{(t)}$, $\mathbf{z}^{(t)}$. At a given sample index t, the minimum in $\mathbf{y}^{(t)}$ and the maximum in $\mathbf{z}^{(t)}$ can be found by taking a derivative of the objective function Eq. (S73) with respect to $\mathbf{y}^{(t)}$ and $\mathbf{z}^{(t)}$, respectively:

$$\begin{aligned}\frac{\partial \operatorname{OF}}{\partial \mathbf{y}^{(t)}} &= \frac{1}{T}\left(-\mathbf{x}^{(t)} + \mathbf{y}^{(t)} + \gamma^2\mathbf{W}^*\mathbf{z}^{(t)}\right)\\ \frac{\partial \operatorname{OF}}{\partial \mathbf{z}^{(t)}} &= \frac{1}{T}\left(\gamma^2\mathbf{W}^{*\top}\mathbf{y}^{(t)} - \frac{\gamma^4}{\rho^2}\mathbf{M}^{(t)}\mathbf{z}^{(t)}\right)\end{aligned} \tag{S74}$$

The minimum in $\mathbf{y}^{(t)}$ and the maximum in $\mathbf{z}^{(t)}$ can be reached by gradient descent and ascent, respectively. We can thus write a system of differential equations whose steady-state correspond to the optima in $\mathbf{y}^{(t)}$ and $\mathbf{z}^{(t)}$:

$$\begin{cases} \tau_y \dfrac{d\mathbf{y}^{(t)}(\tau)}{d\tau} &= -\mathbf{y}^{(t)}(\tau) - \gamma^2 \mathbf{W}^* \mathbf{z}^{(t)}(\tau) + \mathbf{x}^{(t)} \\ \tau_z \dfrac{d\mathbf{z}^{(t)}(\tau)}{d\tau} &= -\mathbf{M}^* \mathbf{z}^{(t)}(\tau) + \rho^2/\gamma^2 \mathbf{W}^{*\top} \mathbf{y}^{(t)}(\tau) \end{cases} \tag{S75}$$

Where τ is the local time evolution variable. We rearranged the parameters so that the equation form is the same as in Eq. (6) in the main text, which does not change the final steady-state of the equations. Thus, we obtained equations to find the optima $\bar{\mathbf{y}}^{(t)}$ and $\bar{\mathbf{z}}^{(t)}$ of the objective function. As explained in the main text, these equations can directly be mapped onto the dynamics of the ORN-LN neural circuit.

Note that for a given input $\mathbf{X}$ there are infinitely many solutions for $\mathbf{Z}$ (see Eq. (S26), Eq. (S28c)), i.e., for any solution $\mathbf{Z}^*$, $\mathbf{QZ}^*$ is also a solution, where $\mathbf{Q}$ is an orthogonal matrix. Therefore changing $\mathbf{W}^*$ to $\mathbf{W}^*\mathbf{Q}^\top$ and $\mathbf{M}^*$ to $\mathbf{QM}^*\mathbf{Q}^\top$ still gives a circuit that solves the original optimization problem. It is possible to construct more circuits that implement the same computations, however, that would require having feedforward ORNs $\rightarrow$ LN connectivity $\mathbf{W}$ not proportional to the feedback LN $\rightarrow$ ORNs, or LN-LN connections (i.e., $\mathbf{M}$) not being symmetric. Here, we focus our analysis on circuits with ORNs $\rightarrow$ LN connectivity proportional to LN $\rightarrow$ ORNs and to $\mathbf{M}$ symmetric. This is reasonable given the data in the connectome (Fig. 4A, Fig. S2A).

B. Circuit equations for the NNC (Eq. (7) in the main text). Here we derive the circuit equations for the NNC (Eq. (7) in the main text). In the case of the NNC, we start with the same optimization problem Eq. (S14), but adding the nonnegative constraints on $\mathbf{Y}$ and $\mathbf{Z}$:

$$\min_{\mathbf{Y}\geq 0} \max_{\mathbf{Z}\geq 0} \frac{1}{T^2} \operatorname{Tr}\left[-T\mathbf{X}^\top\mathbf{Y} + \frac{T}{2}\mathbf{Y}^\top\mathbf{Y} + \frac{\gamma^2}{2u^2}\mathbf{Y}^\top\mathbf{Y}\mathbf{Z}^\top\mathbf{Z} - \frac{\gamma^4}{4u^2\rho^2}\mathbf{Z}^\top\mathbf{Z}\mathbf{Z}^\top\mathbf{Z} \right] \tag{S76}$$

Following the same steps as above we arrive at the optimization problem similar to Eq. (S72) but with nonnegative constraints:

$$\min_{\{\mathbf{y}^{(t)}\geq 0\}} \max_{\{\mathbf{z}^{(t)}\geq 0\}} \mathrm{OF}(\left\{\mathbf{y}^{(t)}\right\}, \left\{\mathbf{z}^{(t)}\right\}) \tag{S77}$$

with the objective function OF as in Eq. (S73).

Here too, we perform the optimization for each $\mathbf{y}^{(t)}$, $\mathbf{z}^{(t)}$. However, because of the nonnegativity constraints, the optima for $\mathbf{y}^{(t)}$ and $\mathbf{z}^{(t)}$ are not to be found where the derivatives Eq. (S74) are zeros. We can, however, reach the optima by a projected gradient descent:

$$\begin{cases} \mathbf{y}^{(t)}(\tau+1) = \max\left[\mathbf{0},\ \mathbf{y}^{(t)}(\tau) + \epsilon(\tau)\big(-\mathbf{y}^{(t)}(\tau) - \gamma^2\mathbf{W}^*\mathbf{z}^{(t)}(\tau) + \mathbf{x}^{(t)}\big)\right] \\ \mathbf{z}^{(t)}(\tau+1) = \max\left[\mathbf{0},\ \mathbf{z}^{(t)}(\tau) + \epsilon(\tau)\big(-\mathbf{M}^*\mathbf{z}^{(t)}(\tau) + \rho^2/\gamma^2\mathbf{W}^{*\top}\mathbf{y}^{(t)}(\tau)\big)\right] \end{cases} \tag{S78}$$

where the max is performed component-wise. Here too, $\mathbf{W}^*$ and $\mathbf{M}^*$ are found by finding $\mathbf{Y}^*$ and $\mathbf{Z}^*$ in the optimization problem Eq. (S76), and setting $\mathbf{W}^* = \mathbf{Y}^*\mathbf{Z}^{*\top}/(Tu^2)$ and $\mathbf{M}^* = \mathbf{Z}^*\mathbf{Z}^{*\top}/(Tu^2)$.

Because of the nonnegativity constraint on $\mathbf{Y}$ and $\mathbf{Z}$ in the NNC, there is no more degree of freedom in $\mathbf{Z}$ as in the LC.

C. Circuit model with Hebbian synaptic update rules (Eq. (8) in the main text). We now show that the circuit can also reach the optimal synaptic weights ($\mathbf{W}^*$ and $\mathbf{M}^*$) via Hebbian plasticity. We derive the Eq. (8) in the main text. The equations are the same for the LC and NNC, therefore we just show the LC here. We start the derivation from Eq. (S71) and Eq. (S70). Next, we exchange the order of the $\min_\mathbf{Y} \max_\mathbf{Z}$ with $\max_\mathbf{W} \min_\mathbf{M}$ (5), giving us the optimization problem:

$$\max_{\mathbf{W}} \min_{\mathbf{M}} \min_{\{\mathbf{y}^{(t)}\}} \max_{\{\mathbf{z}^{(t)}\}} \mathrm{OF}(\mathbf{W}, \mathbf{M}, \{\mathbf{y}^{(t)}\}, \{\mathbf{z}^{(t)}\}) \tag{S79}$$

We now perform the optimization of the 4 variables separately: $\mathbf{y}^{(t)}$, $\mathbf{z}^{(t)}$, $\mathbf{W}$, and $\mathbf{M}$. We alternate the optimization in $\{\mathbf{y}^{(t)}, \mathbf{z}^{(t)}\}$ and in $\{\mathbf{W}, \mathbf{M}\}$, which corresponds to the "online setting" for this optimization problem: as a new sample (i.e., stimulus, input) $\mathbf{x}^{(t)}$ arrives, we find the steady-state values of $\mathbf{z}^{(t)}$ and $\mathbf{y}^{(t)}$ with the current values $\mathbf{W}^{(t)}$ and $\mathbf{M}^{(t)}$ and update $\mathbf{W}^{(t)}$ and $\mathbf{M}^{(t)}$ to $\mathbf{W}^{(t+1)}$ and $\mathbf{M}^{(t+1)}$ before the arrival of the next input sample $\mathbf{x}^{(t+1)}$. Biologically, this can be seen as first a convergence of neural spiking rates or neural electrical potential encoded

through the variables $\mathbf{y}^{(t)}$ and $\mathbf{z}^{(t)}$, and second a synaptic weight update based on those steady-state activity values. The steady-state $\mathbf{y}^{(t)}$ and $\mathbf{z}^{(t)}$ are found in the same way as above, and give us the same equations as Eq. (S75) for the LC and Eq. (S78) for the NNC:

$$\begin{cases} \tau_y \dfrac{d\mathbf{y}^{(t)}(\tau)}{d\tau} = -\mathbf{y}^{(t)}(\tau) - \gamma^2 \mathbf{W}^{(t)}\mathbf{z}^{(t)}(\tau) + \mathbf{x}^{(t)} \\ \tau_z \dfrac{d\mathbf{z}^{(t)}(\tau)}{d\tau} = -\mathbf{M}^{(t)}\mathbf{z}^{(t)}(\tau) + \rho^2/\gamma^2 \mathbf{W}^{(t)\top}\mathbf{y}^{(t)}(\tau) \end{cases} \tag{S80}$$

and

$$\begin{cases} \mathbf{y}^{(t)}(\tau+1) = \max\left[\mathbf{0},\ \mathbf{y}^{(t)}(\tau) + \epsilon(\tau)\big(-\mathbf{y}^{(t)}(\tau) - \gamma^2\mathbf{W}^{(t)}\mathbf{z}^{(t)}(\tau) + \mathbf{x}^{(t)}\big)\right] \\ \mathbf{z}^{(t)}(\tau+1) = \max\left[\mathbf{0},\ \mathbf{z}^{(t)}(\tau) + \epsilon(\tau)\big(-\mathbf{M}^{(t)}\mathbf{z}^{(t)}(\tau) + \rho^2/\gamma^2\mathbf{W}^{(t)\top}\mathbf{y}^{(t)}(\tau)\big)\right] \end{cases} \tag{S81}$$

We only then need to derive the updates for the variables $\mathbf{W}$ and $\mathbf{M}$. By construction, the offline solution for $\mathbf{W}$ and $\mathbf{M}$ is given by Eq. (S67). Online - we compute a new $\mathbf{W}^{(t)}$ and $\mathbf{M}^{(t)}$ after each sample $\mathbf{x}^{(t)}$ is presented and the steady-state solutions of Eq. (S80) or Eq. (S81) $\bar{\mathbf{y}}^{(t)}$ and $\bar{\mathbf{z}}^{(t)}$ are found. The gradient descent (respectively ascent) steps on these variables give the following updates (e.g., (5)):

$$\begin{aligned} \mathbf{W}^{(t+1)} &= \mathbf{W}^{(t)} + \eta^{(t)}\left(\frac{\bar{\mathbf{z}}^{(t)}\bar{\mathbf{y}}^{(t)\top}}{u^2} - \mathbf{W}^{(t)}\right) \\ \mathbf{M}^{(t+1)} &= \mathbf{M}^{(t)} + \frac{\eta^{(t)}}{2\rho^2\nu}\left(\frac{\bar{\mathbf{z}}^{(t)}\bar{\mathbf{z}}^{(t)\top}}{u^2} - \mathbf{M}^{(t)}\right) \end{aligned} \tag{S82}$$

where $\eta^{(t)}$ and ν are parameters of the gradient descent/ascent, and where $\bar{\mathbf{y}}^{(t)}$ and $\bar{\mathbf{z}}^{(t)}$ are the steady-state solutions of Eq. (S80) (or Eq. (S81)) for given $\mathbf{W}^{(t)}$ and $\mathbf{M}^{(t)}$. This indeed corresponds to local Hebbian synaptic update rules. Choosing $\eta^{(t)}$ and ν appropriately will lead to Eq. (8) from the main text.

These synaptic update equations are the same for the LC and the NNC.

D. Steady-state solution of the circuit dynamical equations for the LC and stability. We can directly find the steady-state solution of the circuit dynamics equations Eq. (S75) of the LC by setting the derivatives to 0. For $\mathbf{M}$ invertible, the steady-state is (after dropping the index (t) and the * for simplicity of notation):

$$\begin{cases} \bar{\mathbf{y}} = (\mathbf{I}_D + \rho^2\mathbf{W}\mathbf{M}^{-1}\mathbf{W}^\top)^{-1}\mathbf{x} \\ \bar{\mathbf{z}} = \rho^2/\gamma^2\mathbf{M}^{-1}\mathbf{W}^\top\bar{\mathbf{y}} \end{cases} \tag{S83}$$

As mentioned above, the steady-state for $\mathbf{y}$ does not depend on γ, whereas $\mathbf{z}$ does depend on γ. Note that the transformation from $\mathbf{x}$ to $\bar{\mathbf{y}}$ is symmetric: indeed, writing $\bar{\mathbf{y}} = \mathbf{F}\mathbf{x}$, we have that $\mathbf{F} = \mathbf{F}^\top$. This means that the transformation is diagonalizable. We indeed showed in section 7 above that the basis in which the transformation is the uncentered PCA basis of $\mathbf{X}$.

Here we show that the fixed point of Eq. (S75) is stable if $\mathbf{W}$ is maximum rank and $\mathbf{M}$ positive definite. We first rewrite the dynamical system:

$$\begin{bmatrix} \tau_y d\mathbf{y}(\tau)/d\tau \\ \tau_y d\mathbf{z}(\tau)/d\tau \end{bmatrix} = \begin{bmatrix} \mathbf{x} \\ \mathbf{0} \end{bmatrix} - \begin{bmatrix} \mathbf{I}_D & \gamma^2\mathbf{W} \\ -\rho^2/\gamma^2\mathbf{W}^\top & \mathbf{M} \end{bmatrix}\begin{bmatrix} \mathbf{y}(\tau) \\ \mathbf{z}(\tau) \end{bmatrix} = \begin{bmatrix} \mathbf{x} \\ \mathbf{0} \end{bmatrix} - \mathbf{A}\begin{bmatrix} \mathbf{y}(\tau) \\ \mathbf{z}(\tau) \end{bmatrix} \tag{S84}$$

This system has a unique stable fixed point if and only if $\mathbf{A}$ has only positive eigenvalues. To investigate under which conditions this is the case, we write the eigenvalue equations for $\mathbf{A}$:

$$\begin{bmatrix} \mathbf{I}_D & \gamma^2\mathbf{W} \\ -\rho^2/\gamma^2\mathbf{W}^\top & \mathbf{M} \end{bmatrix}\begin{bmatrix} \mathbf{y} \\ \mathbf{z} \end{bmatrix} = \lambda\begin{bmatrix} \mathbf{y} \\ \mathbf{z} \end{bmatrix} \tag{S85}$$

$$\begin{cases} \mathbf{y} + \gamma^2\mathbf{W}\mathbf{z} = \lambda\mathbf{y} \\ -\rho^2/\gamma^2\mathbf{W}^\top\mathbf{y} + \mathbf{M}\mathbf{z} = \lambda\mathbf{z} \end{cases} \tag{S86}$$

$$\begin{cases} \gamma^2\mathbf{W}\mathbf{z} = (\lambda-1)\mathbf{y} \\ \rho^2/\gamma^2\mathbf{W}^\top\mathbf{y} = (\mathbf{M}-\lambda)\mathbf{z} \end{cases} \tag{S87}$$

We consider the case when $\lambda \neq 1$, as we are interested to see if λ could potentially be negative.

$$\mathbf{y} = (\lambda - 1)^{-1}\gamma^2 \mathbf{W}\mathbf{z} \quad [S88]$$

$$\implies \rho^2 \mathbf{W}^\top \mathbf{W}\mathbf{z} = (\lambda - 1)(\mathbf{M} - \lambda)\mathbf{z} \quad [S89]$$

$\mathbf{W}^\top\mathbf{W} \in \mathbb{R}^{K\times K}$ is a positive semi-definite matrix, it is positive definite if $\mathbf{W}$ is maximum rank (i.e., rank K). Assuming that $\mathbf{W}$ is full rank, the matrix $\mathbf{W}^\top\mathbf{W}$ on the left-hand side of the equation has only positive eigenvalues. The above equation does not have any solution $\mathbf{z} \neq \mathbf{0}$ for $\lambda < 0$ if $\mathbf{M}$ is positive definite (which is true when constructed as the autocorrelator of $\mathbf{z}$). Thus, $\mathbf{W}$ full rank and $\mathbf{M}$ positive definite are sufficient conditions for the dynamical system to always converge to a stable fixed point.

9. Effect of ρ and γ on the computation and the circuit

Having the expression for the optimal outputs $\mathbf{Y}^*$ and $\mathbf{Z}^*$ (section 7), we can describe the effect of ρ and γ on the computation.

For $\rho \to 0$, based on Eq. (S57) we get that $s_{Z,i} \to 0$ and thus $\mathbf{Z}^* \to 0$, leading to $\mathbf{Y}^* = \mathbf{X}$, which means that the output is equal to the input and no inhibition is taking place.

Conversely, for $\rho \to \infty$, according to Eq. (S58) the lowest $D - K$ singular values of $\mathbf{Y}^*$ remain the same, whereas top K drop to 0, i.e., the top K singular values are totally suppressed.

According to Eq. (S58), changing γ has no effect on the output $\mathbf{Y}^*$. This is because, as shown above in section 6C, scaling γ only scales $\mathbf{Z}^*$, but does not alter the optimization. There is a drastic difference between setting $\gamma = 0$ and taking the limit $\gamma \to 0$. In the case of the limit of γ towards 0, it will increase the elements of $\mathbf{Z}^*$ towards infinity, but will not change the value of $\mathbf{Y}^*$. On the other hand, setting γ to 0 in the original optimization problem Eq. (S14) removes all the terms in $\mathbf{Z}$ and we get $\mathbf{Y}^* = \mathbf{X}$, because there is no inhibition.

Next, we inspect the scenario where $\gamma \to 0$ and $\rho \to 0$ such that $\gamma/\rho = C$ where C is a constant. To understand this scenario we make the substitution $\gamma = \rho C$ in Eq. (S57)-Eq. (S61). For $i \leq K$:

$$s_{X,i} = C s^*_{Z,i}\left(1 + \frac{C^2\rho^2}{Tu^2} s^{*2}_{Z,i}\right) \quad [S90]$$

$$s_{X,i} = s^*_{Y,i}\left(1 + \frac{\rho^2}{Tu^2} s^{*2}_{Y,i}\right) \quad [S91]$$

$$s^*_{Y,i} = C s^*_{Z,i} \quad [S92]$$

and for $i > K$ nothing changes. Now taking the limit $\rho \to 0$ (which automatically takes the limit $\gamma \to 0$ since they are related in the constant C), we get:

$$s_{X,i} = s^*_{Y,i} = C s^*_{Z,i} \quad [S93]$$

This means that $\mathbf{Y}^* = \mathbf{X}$, i.e., there is no inhibition, but there is still activity in the LNs ($\mathbf{Z}$). Physiologically this corresponds to the scenario where the is no feedback connections from LNs to ORNs, only feedforward connection from ORNs to LNs - this is then a pure feedforward circuit. What is the consequence in terms of synaptic weights matrices $\mathbf{W}^*$ and $\mathbf{M}^*$ for this situation? By definition, these matrices are given by relations Eq. (S67), repeated here:

$$\mathbf{W}^* = \frac{1}{Tu^2}\mathbf{Y}^*\mathbf{Z}^{*\top}, \quad \mathbf{M}^* = \frac{1}{Tu^2}\mathbf{Z}^*\mathbf{Z}^{*\top} \quad [S94]$$

For the LC, we show below that synaptic weight vectors of $\mathbf{W}^*$ span the same subspace as the first K singular vectors (uncentered PCA directions) as $\mathbf{X}$. This is still the case here. Similarly, there is no difference in terms of LN-LN connection weights $\mathbf{M}^*$ in this particular scenario in comparison to the general one. Similarly, for the NNC case, there is no difference from the general case.

10. Circuit dynamics equations contains two effective parameters (ρ and γ)

Here we show that, in its general form, the system of differential equations describing the olfactory circuit has just two effective parameters and can be reduced to Eq. (6) (or Eq. (7)) from the main text. Without a lack of generality the system of differential equations yields:

$$\begin{cases} \tau_1 \dfrac{d\mathbf{y}(\tau)}{d\tau} = -a\mathbf{y}(\tau) - b\mathbf{W}_1\mathbf{z}(\tau) + a\mathbf{x} \\ \tau_2 \dfrac{d\mathbf{z}(\tau)}{d\tau} = -c\mathbf{M}\mathbf{z}(\tau) + d\mathbf{W}_2^\top\mathbf{y}(\tau) \end{cases} \quad [S95]$$

Where we imposed that $\mathbf{x} = \mathbf{y}$ in the case of no LN activity (i.e., $\mathbf{z} = \mathbf{0}$), that $a > 0$, $b > 0$, $c > 0$, $d > 0$, and that all ORNs have similar response properties (i.e., the same coefficient in front of each x_i and y_i). To extract the effective parameters, we compute the steady-state solution of Eq. (S95) by setting the derivatives to zero. We find the following steady-states for $\mathbf{y}$ and $\mathbf{z}$, for invertible $\mathbf{M}$:

$$\begin{cases} \bar{\mathbf{y}} = \left(\mathbf{I}_D + \frac{bd}{ac}\mathbf{W}_1\mathbf{M}^{-1}\mathbf{W}_2^\top\right)^{-1}\mathbf{x} \\ \bar{\mathbf{z}} = \frac{d}{c}\mathbf{M}^{-1}\mathbf{W}_2^\top\bar{\mathbf{y}} \end{cases} \tag{S96}$$

This shows that we only have two degrees of freedom: $\frac{bd}{ac}$ and $\frac{d}{c}$. We define $\rho^2 := \frac{bd}{ac}$ and $\gamma^2 := \frac{c}{d}\rho^2 = \frac{b}{a}$. This gives us:

$$\begin{cases} \bar{\mathbf{y}} = \left(\mathbf{I}_D + \rho^2\mathbf{W}_1\mathbf{M}^{-1}\mathbf{W}_2^\top\right)^{-1}\mathbf{x} \\ \bar{\mathbf{z}} = \rho^2/\gamma^2\mathbf{M}^{-1}\mathbf{W}_2^\top\bar{\mathbf{y}} \end{cases} \tag{S97}$$

Now replacing these definitions into the original Eq. (S95) we get:

$$\begin{cases} \tau_1/a\frac{d\mathbf{y}(\tau)}{d\tau} = -\mathbf{y}(\tau) - \gamma^2\mathbf{W}_1\mathbf{z}(\tau) + \mathbf{x} \\ \tau_2/c\frac{d\mathbf{z}(\tau)}{d\tau} = -\mathbf{M}\mathbf{z}(\tau) + \rho^2/\gamma^2\mathbf{W}_2^\top\mathbf{y}(\tau) \end{cases} \tag{S98}$$

By setting $\tau_y := \tau_1/a$, $\tau_z := \tau_2/c$ we obtain Eq. (6) from the main text (when $\mathbf{W}_1 = \mathbf{W}_2$):

$$\begin{cases} \tau_y\frac{d\mathbf{y}(\tau)}{d\tau} = -\mathbf{y}(\tau) - \gamma^2\mathbf{W}_1\mathbf{z}(\tau) + \mathbf{x} \\ \tau_z\frac{d\mathbf{z}(\tau)}{d\tau} = -\mathbf{M}\mathbf{z}(\tau) + \rho^2/\gamma^2\mathbf{W}_2^\top\mathbf{y}(\tau) \end{cases} \tag{S99}$$

Thus, scaling $\mathbf{x}$, $\mathbf{W}_1$, $\mathbf{W}_2$ and $\mathbf{M}$ is equivalent to controlling just two effective parameter γ and ρ. Scaling τ_y and τ_z does not influence the steady-state solutions.

Increasing ρ increases the weight of feedforward connections, making the LN activity and the feedback inhibition stronger. Increasing γ simultaneously increases the feedback connection strength and decreases the feedforward connection strength. Changing γ influences the steady-state solution $\bar{\mathbf{z}}$ but not $\bar{\mathbf{y}}$. Thus, a manifold of circuits leads to the same steady-state output $\bar{\mathbf{y}}$. In addition, the same differential equations can be implemented by different circuits. For example, multiplying a differential equation by a parameter does not alter the final steady-state, but gives yet another implementation to the circuit as a scaling of the synaptic weights and of the time constant.

11. Relationship between $\mathbf{W}$ and $\mathbf{M}$ (Eq. (2) in the main text)

Here we prove the relationship $\rho^2/\gamma^2\mathbf{W}^\top\mathbf{W} = \mathbf{M}^2 = \mathbf{M}^\top\mathbf{M}$ for the LC. In this section, for simplicity we dropped the * from $\mathbf{M}^*$, $\mathbf{W}^*$, $\mathbf{Y}^*$, $\mathbf{Z}^*$, and the related variables.

One way to obtain this relationship is to start from the circuit dynamics (Eq. (S75)). The steady-state for $\bar{\mathbf{z}}^{(t)}$ is:

$$\rho^2/\gamma^2\mathbf{W}^\top\bar{\mathbf{y}}^{(t)} = \mathbf{M}\bar{\mathbf{z}}^{(t)} \tag{S100}$$

Multiplying by $\bar{\mathbf{z}}^{(t)\top}$ on both sides, taking the average over all samples t, and using the definition of $\mathbf{W}$ and $\mathbf{M}$ (Eq. (S67)):

$$\rho^2/\gamma^2\mathbf{W}^\top\mathbf{E}\left[\bar{\mathbf{y}}^{(t)}\bar{\mathbf{z}}^{(t)\top}\right]/u^2 = \mathbf{M}\mathbf{E}\left[\bar{\mathbf{z}}^{(t)}\bar{\mathbf{z}}^{(t)\top}\right]/u^2 \tag{S101}$$

$$\rho^2/\gamma^2\mathbf{W}^\top\mathbf{W} = \mathbf{M}^2 \tag{S102}$$

An alternative approach to derive the above relationship is to use the definition of $\mathbf{W}$ and $\mathbf{M}$ (Eq. (S67)) and the SVD decomposition of $\mathbf{X}$, $\mathbf{Y}$, and $\mathbf{Z}$. We write out $\mathbf{W}$ and $\mathbf{M}$:

$$\mathbf{W} = \frac{1}{Tu^2}\mathbf{Y}\mathbf{Z}^\top = \frac{1}{Tu^2}\mathbf{U}_Y\tilde{\mathbf{S}}_Y\mathbf{V}_Y^\top\mathbf{V}_Z\tilde{\mathbf{S}}_Z^\top\mathbf{U}_Z^\top = \frac{1}{Tu^2}\mathbf{U}_X\tilde{\mathbf{S}}_Y\tilde{\mathbf{S}}_Z^\top\mathbf{U}_Z^\top = \frac{\gamma}{Tu^2\rho}\mathbf{U}_{X|K}\hat{\mathbf{S}}_Z^2\mathbf{U}_Z^\top \tag{S103}$$

$$\mathbf{M} = \frac{1}{Tu^2}\mathbf{Z}\mathbf{Z}^\top = \frac{1}{Tu^2}\mathbf{U}_Z\tilde{\mathbf{S}}_Z\mathbf{V}_Z^\top\mathbf{V}_Z\tilde{\mathbf{S}}_Z^\top\mathbf{U}_Z^\top = \frac{1}{Tu^2}\mathbf{U}_Z\hat{\mathbf{S}}_Z^2\mathbf{U}_Z^\top \tag{S104}$$

Where we used that $\mathbf{V}_X = \mathbf{V}_Y = \mathbf{V}_Z$ and $\mathbf{U}_X = \mathbf{U}_Y$ are orthogonal matrices and that $s_{Y,i} = \frac{\gamma}{\rho} s_{Z,i}$ for $i \leq K$ and $s_{Z,i} = 0$ for $i > K$. We call $\hat{\mathbf{S}}_Z \in \mathbb{R}^{K\times K}$ the square submatrix of the rectangular matrix $\mathbf{S}_Z \in \mathbb{R}^{K\times N}$. $\mathbf{U}_{X|K} \in \mathbb{R}^{D\times K}$ is the submatrix with the first K columns of $\mathbf{U}_X$. Thus:

$$\mathbf{W}^\top \mathbf{W} = \frac{\gamma^2}{T^2 u^4 \rho^2} \mathbf{U}_Z \hat{\mathbf{S}}_Z^2 \mathbf{U}_{X|K}^\top \mathbf{U}_{X|K} \hat{\mathbf{S}}_Z^2 \mathbf{U}_Z^\top \tag{S105}$$

$$= \frac{\gamma^2}{T^2 u^4 \rho^2} \mathbf{U}_Z \hat{\mathbf{S}}_Z^4 \mathbf{U}_Z^\top = \frac{\gamma^2}{\rho^2} \mathbf{M}^2 \tag{S106}$$

Since $\mathbf{M}$ is a symmetric matrix, i.e., $\mathbf{M} = \mathbf{M}^\top$, this relationship can also be written as:

$$\mathbf{W}^\top \mathbf{W} = \frac{\gamma^2}{\rho^2} \mathbf{M}^\top \mathbf{M} \tag{S107}$$

This ends the derivation.

Taking the unique square root on both sides gives the relationship Eq. (2) in the results section of the main text.

A. Consequence of the matrix relationship. We can inspect the consequence of this relation on an element-per-element basis. We call $\mathbf{m}_i$ the ith column of $\mathbf{M}$, which corresponds to the vector of synaptic weight from LN_i onto all the other LNs. We get that:

$$\mathbf{w}_i^\top \mathbf{w}_j = \gamma^2/\rho^2 \mathbf{m}_i^\top \mathbf{m}_j \tag{S108}$$

$$\|\mathbf{w}_i\| \|\mathbf{w}_j\| \cos(\theta_{ij}^w) = \gamma^2/\rho^2 \|\mathbf{m}_i\| \|\mathbf{m}_j\| \cos(\theta_{ij}^m) \tag{S109}$$

Where θ_{ij}^w is the angle between the vectors $\mathbf{w}_i$ and $\mathbf{w}_j$, θ_{ij}^m is the angle between $\mathbf{m}_i$ and $\mathbf{m}_j$; and where we used the scalar product property.

For the elements on the diagonal ($i = j$), we get: $\|\mathbf{w}_i\| = \gamma/\rho \|\mathbf{m}_i\|$. This implies that $\|\mathbf{w}_i\|/\|\mathbf{m}_i\| = \text{const}$, meaning that the ratio between the magnitude of the ORNs → LN and LNs → LN synaptic weight vectors is the same at each LN. We call magnitude the square root of the sum of the squared connection weights, corresponding to the length of the synaptic weight vector and a proxy for the total synaptic strength of a synaptic weight vector.

Feeding $\|\mathbf{w}_i\| = \gamma/\rho \|\mathbf{m}_i\|$ into Eq. (S109), we get that $\theta_{ij}^w = \theta_{ij}^m$, meaning that the angle between $\mathbf{w}_i$ and $\mathbf{w}_j$ is the same as the angle between $\mathbf{m}_i$ and $\mathbf{m}_j$. In other words $\measuredangle(\mathbf{w}_i, \mathbf{w}_j) = \measuredangle(\mathbf{m}_i, \mathbf{m}_j)$, where $\measuredangle(\mathbf{a}, \mathbf{b})$ is the angle between two vectors $\mathbf{a}$ and $\mathbf{b}$. Thus 2 LNs with a similar (different) connectivity pattern with the ORNs have a similar (different) connectivity pattern with LNs.

12. Relationship between ORN activity and ORN-LN connectivity (Eq. (1) in the main text)

In this section, for simplicity we dropped the * from $\mathbf{M}^*$, $\mathbf{W}^*$, $\mathbf{Y}^*$, $\mathbf{Z}^*$, and the related variables. Based on the expressions for $\mathbf{W}$ and $\mathbf{M}$ (Eq. (S103) and Eq. (S104)) we can write $\mathbf{W}$ as:

$$\mathbf{W} = \frac{\gamma}{Tu^2\rho} \mathbf{U}_{X|K} \hat{\mathbf{S}}_Z^2 \mathbf{U}_Z^\top = \frac{\gamma}{Tu^2\rho} \mathbf{U}_{X|K} \mathbf{U}_Z^\top \mathbf{U}_Z \hat{\mathbf{S}}_Z^2 \mathbf{U}_Z^\top = \frac{\gamma}{\rho} \mathbf{U}_{X|K} \mathbf{U}_Z^\top \mathbf{M} \tag{S110}$$

Where we used that $\mathbf{U}_Z^\top \mathbf{U}_Z = \mathbf{I}_K$. Where $\mathbf{U}_{X|K} \in \mathbb{R}^{D\times K}$ is the submatrix with the first K columns of $\mathbf{U}_X$. As stated above, $\mathbf{U}_Z$ is a free parameter and could be any orthogonal matrix.

In the case of a single LN, $\mathbf{W}$ is a column vector and corresponds to the first left eigenvector of $\mathbf{X}$. For multiple LNs, the column vectors of $\mathbf{W}$ span the same subspace as the top K loading vectors of $\mathbf{X}$, $\mathbf{U}_{X|K}$. However, because of the multiplication on the right by $\mathbf{U}_Z^\top \mathbf{M}$, the connections vectors do not necessarily correspond to specific PCA directions and are not orthogonal, but only span the top K-dimensional PCA subspace. Thus, this relation above gives us the relationship between the left eigenvectors of $\mathbf{X}$, $\mathbf{W}$, and $\mathbf{M}$.

13. Decrease of the spread of PCA variances in ORN axons vs soma in the LC

Here we show that the coefficient of variation (CV_σ, i.e., the spread) of PCA variances ($\{\sigma_i^2\}$) is smaller at the ORN output (axons) than at the input (somas) in the LC model when the number of ORNs (D) equal to the number of LN (K), i.e., $D = K$. In that case, we have $\sigma_X = \sigma_Y(1 + \rho^2 \sigma_Y^2)$. As we have shown, for small σ_X, we have $\sigma_Y \approx \sigma_X$

and for large σ_X, we have $\sigma_Y \approx \sqrt[3]{\sigma_X/\rho^2}$. We call X a positive random variable, representing the variances. We will show that for a $0 < \alpha < 1$, $\mathrm{CV}(X) \geq \mathrm{CV}(X^\alpha)$, which mimics the case we have.

$$\mathrm{CV}(X) \geq \mathrm{CV}(X^\alpha) \tag{S111}$$

$$\iff \frac{\sigma_X}{\mathbf{E}[X]} \geq \frac{\sigma_{X^\alpha}}{\mathbf{E}[X^\alpha]} \tag{S112}$$

$$\iff \frac{\sigma_X^2}{\mathbf{E}[X]^2} \geq \frac{\sigma_{X^\alpha}^2}{\mathbf{E}[X^\alpha]^2} \tag{S113}$$

$$\iff \frac{\mathbf{E}[X^2] - \mathbf{E}[X]^2}{\mathbf{E}[X]^2} \geq \frac{\mathbf{E}[X^{2\alpha}] - \mathbf{E}[X^\alpha]^2}{\mathbf{E}[X^\alpha]^2} \tag{S114}$$

$$\iff \frac{\mathbf{E}[X^2]}{\mathbf{E}[X]^2} \geq \frac{\mathbf{E}[X^{2\alpha}]}{\mathbf{E}[X^\alpha]^2} \tag{S115}$$

The last inequality can be proven by using Hölder's inequality twice. First:

$$\left(\mathbf{E}[X^2]\right)^{\frac{1-\alpha}{2-\alpha}} \left(\mathbf{E}[X^\alpha]\right)^{\frac{1}{2-\alpha}} \geq \mathbf{E}[X] \tag{S116}$$

which leads to:

$$\frac{\mathbf{E}[X^2]}{\mathbf{E}[X]^2} \geq \frac{\left(\mathbf{E}[X^2]\right)^{\frac{\alpha}{2-\alpha}}}{\left(\mathbf{E}[X^\alpha]\right)^{\frac{2}{2-\alpha}}} \tag{S117}$$

and second:

$$\left(\mathbf{E}[X^2]\right)^{\frac{\alpha}{2-\alpha}} \left(\mathbf{E}[X^\alpha]\right)^{\frac{2-2\alpha}{2-\alpha}} \geq \mathbf{E}[X^{2\alpha}] \tag{S118}$$

which leads to:

$$\frac{\left(\mathbf{E}[X^2]\right)^{\frac{\alpha}{2-\alpha}}}{\left(\mathbf{E}[X^\alpha]\right)^{\frac{2}{2-\alpha}}} \geq \frac{\mathbf{E}[X^{2\alpha}]}{\mathbf{E}[X^\alpha]^2} \tag{S119}$$

Combining inequalities Eq. (S117) and Eq. (S119) proves inequality Eq. (S115) and ends the proof.

Thus, for an LC with the same number of LNs as ORNs (i.e., $K = D$), the computation in the LC decreases the spread of $\{\sigma_{Y,i}^2\}$ relatively to the spread of $\{\sigma_{X,i}^2\}$. Although for $K < D$, the variance of only the top K PCA direction is decreased, in most cases the computation in the LC also leads to a decrease of CV_σ (Fig. 6D).

14. Numerical simulations of the LC and NNC

A. Numerical simulation of the LC offline. For the LC, we have the theoretical solution, so numerical simulations are not necessary to obtain the optima $\mathbf{Y}^*$ and $\mathbf{Z}^*$. Also, there is a manifold of solutions of $\mathbf{Z}^*$, $\mathbf{W}^*$, and $\mathbf{M}^*$. However, to confirm the theoretical results, we simulate the LC too. For that, we use the optimization problem that depends on $\mathbf{Z}$ only (Eq. (S40), with $\gamma = 1$ and u dropped):

$$\min_{\mathbf{Z}} \frac{1}{T^2} \mathrm{Tr}\left[\frac{T}{2}\mathbf{X}^\top\mathbf{X}\left(\mathbf{I}_T + \frac{1}{T}\mathbf{Z}^\top\mathbf{Z}\right)^{-1} + \frac{1}{4\rho^2}\mathbf{Z}^\top\mathbf{Z}\mathbf{Z}^\top\mathbf{Z}\right] \tag{S120}$$

We use an algorithm similar to (7).

Algorithm 1 Finding the minimum of
$f(\mathbf{Z}) := \mathrm{Tr}\left[\frac{T}{2}\mathbf{X}^\top\mathbf{X}\left(\frac{\mathbf{Z}^\top\mathbf{Z}}{T}+\mathbf{I}_T\right)^{-1}+\frac{1}{4\rho^2}\mathbf{Z}^\top\mathbf{Z}\mathbf{Z}^\top\mathbf{Z}\right]$

Objective: find $\mathbf{Z}^* \in \mathbb{R}^{K\times T}$ that minimizes $f(\mathbf{Z})$.
Inputs:
$\mathbf{X} \in \mathbb{R}^{D\times T}$
$K > 0$: the number of dimensions of $\mathbf{Z}$
$\rho > 0$: a constant encoding the strength of the inhibition by the LNs
$0 < \sigma < 1$: acceptance parameter (usually 0.1)
$\alpha_0 > 0$: initial gradient step coefficient (usually 1 or 10)
$0 < \beta < 1$: reduction factor (usually 0.1 or 0.5)
$0 < \mu \ll 1$: tolerance parameter (usually $\approx 10^{-6}$)
$n_{cycle} \approx 500$: number of steps after which one decreases the value of α_0
Initialize:
$\mathbf{Z}_{new} \in \mathbb{R}^{K\times N} \sim \mathcal{N}(0, \mathrm{SD}(\mathbf{X})/100)$
$i \leftarrow 1$
Iterate:
repeat
  $\mathbf{Z} \leftarrow \mathbf{Z}_{new}$
  $\alpha = \alpha_0$
  repeat
    $\mathbf{Z}_{new} = \mathbf{Z} - \alpha\nabla f(\mathbf{Z})$ ▷ Find a potential new $\mathbf{Z}$ through a gradient descent step
    $\widehat{\Delta f} = \sigma \cdot \mathrm{sum}[\nabla f(\mathbf{Z}) \odot (\mathbf{Z}_{new} - \mathbf{Z})]$ ▷ Acceptable decrease in f (negative number)
    $\Delta f = f(\mathbf{Z}_{new}) - f(\mathbf{Z})$ ▷ True decrease in f (negative number)
    $\alpha \leftarrow \beta\alpha$ ▷ Decrease the gradient descent step size for the next iteration, if it occurs
  until $\Delta f < \widehat{\Delta f}$ ▷ Exit loop if the true decrease in f is larger than the acceptable one
  if $i \mod n_{cycle} = 0$ **then** ▷ Every n_{cycle}, decrease the initial step size α_0 by β
    $\alpha_0 \leftarrow \beta\alpha_0$
  $i \leftarrow i + 1$
until $|f(\mathbf{Z}) - f(\mathbf{Z}_{new})|/|f(\mathbf{Z})| < \mu$
Output: $\mathbf{Z}_{new}$

Where $\odot$ is an element-wise multiplication and the "sum" adds all the elements of the matrix. In the inner repeat loop of the algorithm, it can happen that because of limited numerical precision, no α is small enough to make a decrease in f (i.e., satisfy the condition $\Delta f < \widehat{\Delta f}$), in that case, the inner and outer repeat loops stop and the current $\mathbf{Z}$ (not $\mathbf{Z}_{new}$) is outputted.

$\nabla f(\mathbf{Z})$ is given by:

$$\mathbf{B} := \left(\mathbf{Z}^\top\mathbf{Z}/T + \mathbf{I}\right)^{-1} \tag{S121}$$

$$\nabla f(\mathbf{Z}) = -\mathbf{Z}\mathbf{B}\mathbf{X}\mathbf{X}^\top\mathbf{B} + \mathbf{Z}\mathbf{Z}^\top\mathbf{Z}/\rho^2 \tag{S122}$$

Finally, the expression for $\mathbf{Y}$ is (Eq. (S33)):

$$\mathbf{Y} = \mathbf{X}\left(\mathbf{I}_T + \frac{1}{T}\mathbf{Z}^\top\mathbf{Z}\right)^{-1} \tag{S123}$$

B. Numerical simulation of the NNC offline. For the NNC, we do not have the analytical expressions of $\mathbf{Y}$ and $\mathbf{Z}$. To optimize the objective function, we perform alternating gradient descent/ascent steps on $\mathbf{Y}$ and $\mathbf{Z}$, respectively. We start from the expanded expression of the optimization problem Eq. (S14) with nonnegativity constraints (with $\gamma = 1$ and u dropped):

$$\min_{\mathbf{Y}\geq 0}\max_{\mathbf{Z}\geq 0} \frac{1}{T^2}\mathrm{Tr}\left[-T\mathbf{X}^\top\mathbf{Y} + \frac{T}{2}\mathbf{Y}^\top\mathbf{Y} + \frac{1}{2}\mathbf{Y}^\top\mathbf{Y}\mathbf{Z}^\top\mathbf{Z} - \frac{1}{4\rho^2}\mathbf{Z}^\top\mathbf{Z}\mathbf{Z}^\top\mathbf{Z}\right] \tag{S124}$$

Algorithm 2 Finding the minimum in $\mathbf{Y}$ and maximum in $\mathbf{Z}$ of $f(\mathbf{Y},\mathbf{Z}) := \mathrm{Tr}\left[-T\mathbf{X}^\top\mathbf{Y} + \frac{T}{2}\mathbf{Y}^\top\mathbf{Y} + \frac{1}{2}\mathbf{Y}^\top\mathbf{Y}\mathbf{Z}^\top\mathbf{Z} - \frac{1}{4\rho^2}\mathbf{Z}^\top\mathbf{Z}\mathbf{Z}^\top\mathbf{Z}\right]$

Objective: find $\mathbf{Y}^* \in \mathbb{R}_+^{D\times T}$ and $\mathbf{Z}^* \in \mathbb{R}_+^{K\times T}$ that optimize $\min_{\mathbf{Y}} \max_{\mathbf{Z}} f(\mathbf{Y},\mathbf{Z})$.
Inputs:
$\mathbf{X} \in \mathbb{R}^{D\times T}$
$K > 0$: the number of dimensions of $\mathbf{Z}$
$\rho > 0$: a constant encoding the strength of the inhibition by the LNs
$0 < \sigma < 1$: acceptance parameter (usually 0.1)
$\alpha_0 > 0$: initial gradient step coefficient (usually 1 or 10)
$0 < \beta < 1$: reduction factor (usually 0.1 or 0.5)
$0 < \mu \ll 1$: tolerance parameter (usually $\approx 10^{-6}$)
$n_{cycle} \approx 500$: number of steps after which one decreases the value of α_0
Initialize:
$\mathbf{Y}_{new} \in \mathbb{R}_+^{D\times N} \sim \mathrm{abs}[\mathcal{N}(0, \mathrm{SD}(\mathbf{X})/100)]$
$\mathbf{Z}_{new} \in \mathbb{R}_+^{K\times N} \sim \mathrm{abs}[\mathcal{N}(0, \mathrm{SD}(\mathbf{X})/100)]$
$i \leftarrow 1$
Iterate:
repeat
    $(\mathbf{Y},\mathbf{Z}) \leftarrow (\mathbf{Y}_{new},\mathbf{Z}_{new})$
    $\alpha = \alpha_0$
    repeat
        $\mathbf{Y}_{new} = [\mathbf{Y} - \alpha\nabla_{\mathbf{Y}} f(\mathbf{Y},\mathbf{Z})]_+$ ▷ Find a potential new $\mathbf{Y}$ through a gradient descent step
        $\widehat{\Delta f} = \sigma \cdot \mathrm{sum}[\nabla_{\mathbf{Y}} f(\mathbf{Y},\mathbf{Z}) \odot (\mathbf{Y}_{new} - \mathbf{Y})]$ ▷ Acceptable decrease in f (negative number)
        $\Delta f = f(\mathbf{Y}_{new},\mathbf{Z}) - f(\mathbf{Y},\mathbf{Z})$ ▷ True decrease in f (negative number)
        $\alpha \leftarrow \beta\alpha$ ▷ Decrease the gradient descent step size for the next iteration, if it occurs
    until $\Delta f < \widehat{\Delta f}$ ▷ Exit loop if the true decrease in f is larger than the acceptable one
    $\alpha = \alpha_0$
    repeat
        $\mathbf{Z}_{new} = [\mathbf{Z} + \alpha\nabla_{\mathbf{Z}} f(\mathbf{Y}_{new},\mathbf{Z})]_+$ ▷ find a potential new $\mathbf{Z}$ through a gradient ascend step
        $\widehat{\Delta f} = \sigma \cdot \mathrm{sum}[\nabla_{\mathbf{Z}} f(\mathbf{Y}_{new},\mathbf{Z}) \odot (\mathbf{Z}_{new} - \mathbf{Z})]$ ▷ Acceptable increase in f (positive number)
        $\Delta f = f(\mathbf{Y}_{new},\mathbf{Z}_{new}) - f(\mathbf{Y}_{new},\mathbf{Z})$ ▷ True increase in f (positive number)
        $\alpha \leftarrow \beta\alpha$ ▷ Decrease the ascent descent step size for the next iteration, if it occurs
    until $\Delta f > \widehat{\Delta f}$ ▷ Exit loop if the true increase in f is larger than the acceptable one
    if $i \mod n_{cycle} = 0$ **then** ▷ Every n_{cycle}, decrease the initial step size α_0 by β
        $\alpha_0 \leftarrow \beta\alpha_0$
    $i \leftarrow i + 1$
until $|f(\mathbf{Y},\mathbf{Z}) - f(\mathbf{Y}_{new},\mathbf{Z})|/|f(\mathbf{Y},\mathbf{Z})| < \mu$ and $|f(\mathbf{Y}_{new},\mathbf{Z}) - f(\mathbf{Y}_{new},\mathbf{Z}_{new})|/|f(\mathbf{Y}_{new},\mathbf{Z})| < \mu$
Output: $\mathbf{Y}_{new}, \mathbf{Z}_{new}$

Where $[\mathbf{a}]_+ = \max[\mathbf{0}, \mathbf{A}]$, is an element-wise rectification. In the case of the LC, this algorithm holds as well, with all the rectifications $[.]_+$ removed and the "abs" removed from the initiation. If in either of the inner repeat loops, no α is small enough to make a decrease/increase in f (i.e., satisfy the condition $\Delta f < \widehat{\Delta f}$ or $\Delta f > \widehat{\Delta f}$), the iterations stop and the current $\mathbf{Y}$ and $\mathbf{Z}$ are the output of the algorithm.

The gradients of $f(\mathbf{Y},\mathbf{Z})$ are:

$$\nabla_{\mathbf{Y}} f(\mathbf{Y},\mathbf{Z}) = -T(\mathbf{X} - \mathbf{Y}) + \mathbf{Y}\mathbf{Z}^\top\mathbf{Z} \quad \text{[S125]}$$

$$\nabla_{\mathbf{Z}} f(\mathbf{Y},\mathbf{Z}) = \mathbf{Z}\mathbf{Y}^\top\mathbf{Y} - \mathbf{Z}\mathbf{Z}^\top\mathbf{Z}/\rho^2 \quad \text{[S126]}$$

C. Numerical simulation of the circuits online. For Fig. S17, we simulated the circuit dynamics for a given $\mathbf{W}$, $\mathbf{M}$, and $\mathbf{X}$. For that purpose, to find $\mathbf{y}^*$ and $\mathbf{z}^*$, we performed gradient descent steps based on the discretized Eq. (S75) for the LC or Eq. (S78) for the NNC (correspondingly Eq. (6) and Eq. (7) in the main text).

15. Simulation of the circuit with synaptic weights from the connectome (Fig. S15)

We investigate the computation performed by a nonnegative ORN-LN circuit where the synaptic weights are set proportionally to the synaptic counts from the connectome (1) (Section 15). Given that we have a connectome for the left and right sides of the larva, there are two such circuit models. We call this model NNC-conn. It has 8 LNs.

Several aspects are worth mentioning regarding this model. Two main reasons might make the results of these simulations not entirely trustworthy and the computation performed by this circuit might not necessarily represent the true computation in the real biological circuit. First, because several physiological parameters are not available and are guessed: neuronal leaks, the ratios of the synaptic strengths of ORNs → LNs vs LNs → ORNs vs LNs → LNs. Second, the observed computation of a circuit strongly depends on the input it receives. Since we do not know the true input statistics to which this circuit model is adapted to, the observed computation might be misleading. This simulation is rather a control of whether the predictions of the NNC model are somewhat compatible with the potential computation done using the synaptic counts.

To simulate this circuit, we thus first need to choose a scaling for the synaptic counts found in the connectome, in order to convert them to synaptic weights (note that the circuit contains both excitatory and inhibitory synapses, and their relative strength is unknown). To perform that transformation, we divide the ORN → LN counts by 80, divide the LN → ORN counts by 30, and divide the LN →LN counts by 60. These numbers are roughly the average of the norms of the columns of the matrices $\mathbf{W}^{\text{ff}}$, $\mathbf{W}^{\text{fb}}$, and $\mathbf{M}$, respectively. We choose these scaling factors to ensure that the synaptic strengths are somewhat comparable between different directions of activity flow (i.e., ORNs to LN, LNs to ORN, LNs to LN). Next, we need to choose values for the diagonal of $\mathbf{M}$, which correspond to the neural leaks of LNs and which are not known. We set those values to the maximum of each column of $\mathbf{M}$, which makes the neural leak (i.e., self-inhibition) comparable to the inhibition coming from other LNs. We then simulate this circuit for the left and right sides of the larva. In Fig. S15, we show the average between the left and right side for ORN activity, and we show the LN activity separately for the left and right. We use the same equations as for the NNC to simulate the circuit (Eq. (S78), Eq. (7) in the main text), having adapted the formulas to incorporate different feedforward and feedback connectivity.

Finally, given the multidimensional space of unknown parameters, different modes of computation could arise in different regions of the parameter space. These modes of computation might not correspond to the true computation of the actual biological circuit. To be more accurate, this bottom-up approach would require an in-depth investigation of a large parameter space to see what different modes of operation this circuit could have and then evaluate their plausibility. More physiological recordings of this circuit would allow making such bottom-up models more reliable.

16. Optimization problem for circuit without LN-LN connections (Fig. S18)

The following optimization problem provides a circuit without LN-LN connections (8):

$$\min_{\mathbf{Y}} \max_{\mathbf{Z}} \frac{1}{T^2} \operatorname{Tr}\left[-T\mathbf{X}^\top\mathbf{Y} + \frac{T}{2}\mathbf{Y}^\top\mathbf{Y} + \frac{\gamma^2}{2u^2}\mathbf{Y}^\top\mathbf{Y}\mathbf{Z}^\top\mathbf{Z} - \frac{T\gamma^2}{2\rho^2}\mathbf{Z}^\top\mathbf{Z}\right] \tag{S127}$$

Where the variables and parameters are the same as for the optimization problem Eq. (S14). $\mathbf{Z}^\top\mathbf{Z}\mathbf{Z}^\top\mathbf{Z}$ has been replaced with $\mathbf{Z}^\top\mathbf{Z}$ and parameters arranged accordingly. It can help to see that this objective function implements whitening by rewriting it as follow:

$$\min_{\mathbf{Y}} \max_{\mathbf{Z}} \frac{1}{2T}\|\mathbf{X}-\mathbf{Y}\|_F^2 + \frac{1}{T^2}\operatorname{Tr}\left[\frac{\gamma^2}{2}\mathbf{Z}^\top\mathbf{Z}\left(\frac{1}{u^2}\mathbf{Y}^\top\mathbf{Y} - \frac{T}{\rho^2}\mathbf{I_T}\right)\right] \tag{S128}$$

Where $\mathbf{Z}^\top\mathbf{Z}$ acts like a Lagrange multiplier.

A. Online solution. Following a similar approach as with the optimization problem Eq. (S14), we find, analogously to Eq. (S80) that the online algorithm that can be implemented by a circuit model is:

$$\begin{cases} \tau_y \dfrac{d\mathbf{y}^{(t)}(\tau)}{d\tau} = -\mathbf{y}^{(t)}(\tau) - \gamma^2\mathbf{W}^{(t)}\mathbf{z}^{(t)}(\tau) + \mathbf{x}^{(t)} \\ \tau_z \dfrac{d\mathbf{z}^{(t)}(\tau)}{d\tau} = -\mathbf{z}^{(t)}(\tau) + \rho^2\mathbf{W}^{(t)\top}\mathbf{y}^{(t)}(\tau) \end{cases} \tag{S129}$$

As one can see, there is no interactions between LNs. The synaptic updates are (see Eq. (S82)):

$$\mathbf{W}^{(t+1)} = \mathbf{W}^{(t)} + \eta^{(t)}\left(\frac{\bar{\mathbf{z}}^{(t)}\bar{\mathbf{y}}^{(t)\top}}{u^2} - \mathbf{W}^{(t)}\right) \tag{S130}$$

Similar to Eq. (S81), in the nonnegative version of the optimization problem Eq. (S127), the circuit equations become

$$\begin{cases} \mathbf{y}^{(t)}(\tau+1) = \max\left[\mathbf{0},\ \mathbf{y}^{(t)}(\tau) + \epsilon(\tau)\big(-\mathbf{y}^{(t)}(\tau) - \gamma^2\mathbf{W}^{(t)}\mathbf{z}^{(t)}(\tau) + \mathbf{x}^{(t)}\big)\right] \\ \mathbf{z}^{(t)}(\tau+1) = \max\left[\mathbf{0},\ \mathbf{z}^{(t)}(\tau) + \epsilon(\tau)\big(-\mathbf{z}^{(t)}(\tau) + \rho^2\mathbf{W}^{(t)\top}\mathbf{y}^{(t)}(\tau)\big)\right] \end{cases} \quad \text{[S131]}$$

B. Circuit computation. Using similar methods as above, we find that the solution of the optimization problem Eq. (S127) is:

$$\mathbf{Y}^* = \mathbf{U}_X\tilde{\mathbf{S}}_Y^*\mathbf{V}_X^\top \quad \text{[S132]}$$

$$\mathbf{Z}^* = \rho/\gamma\mathbf{U}_Z^*\tilde{\mathbf{S}}_Z^*\mathbf{V}_X^\top \quad \text{[S133]}$$

$$\text{with} \begin{cases} s_{Y,i}^* = \min\left(s_{X,i}, \dfrac{u\sqrt{T}}{\rho}\right) & 1 \le i \le K \quad \text{[S134a]} \\ s_{Y,i}^* = s_{X,i} & K+1 \le i \le D \quad \text{[S134b]} \\ s_{Z,i}^* = \sqrt{\dfrac{Tu^2}{\gamma^2}\left(\dfrac{\rho s_{X,i}}{u\sqrt{T}} - 1\right)} & s_{X,i} \ge u\sqrt{T}/\rho \quad \text{[S134c]} \\ s_{Z,i}^* = 0 & s_{X,i} < u\sqrt{T}/\rho \quad \text{[S134d]} \\ \mathbf{U}_Z^*\text{: a degree of freedom} & \quad \text{[S134e]} \end{cases}$$

This means that in the output $\mathbf{Y}^*$, all the top K (as the number of LNs) PCA variances become equal to $\frac{u^2}{\rho^2}$ or stay the same as in the input $\mathbf{X}$ if the original variance is smaller than $\frac{u^2}{\rho^2}$. If $K \ge D$ (i.e., the number of LNs is equal or more than the number of input neurons) and all original variances are larger than $\frac{u^2}{\rho^2}$, then the output $\mathbf{Y}^*$ will be white: all variance will be $\frac{u^2}{\rho^2}$. We have used the relationship between the PCA variance σ^2 and the singular value s: $s^2/T = \sigma^2$.

C. Numerical simulations. Numerical simulations for these objective functions are performed using the same methodology as for the original optimization problem.

Supplementary figures and tables

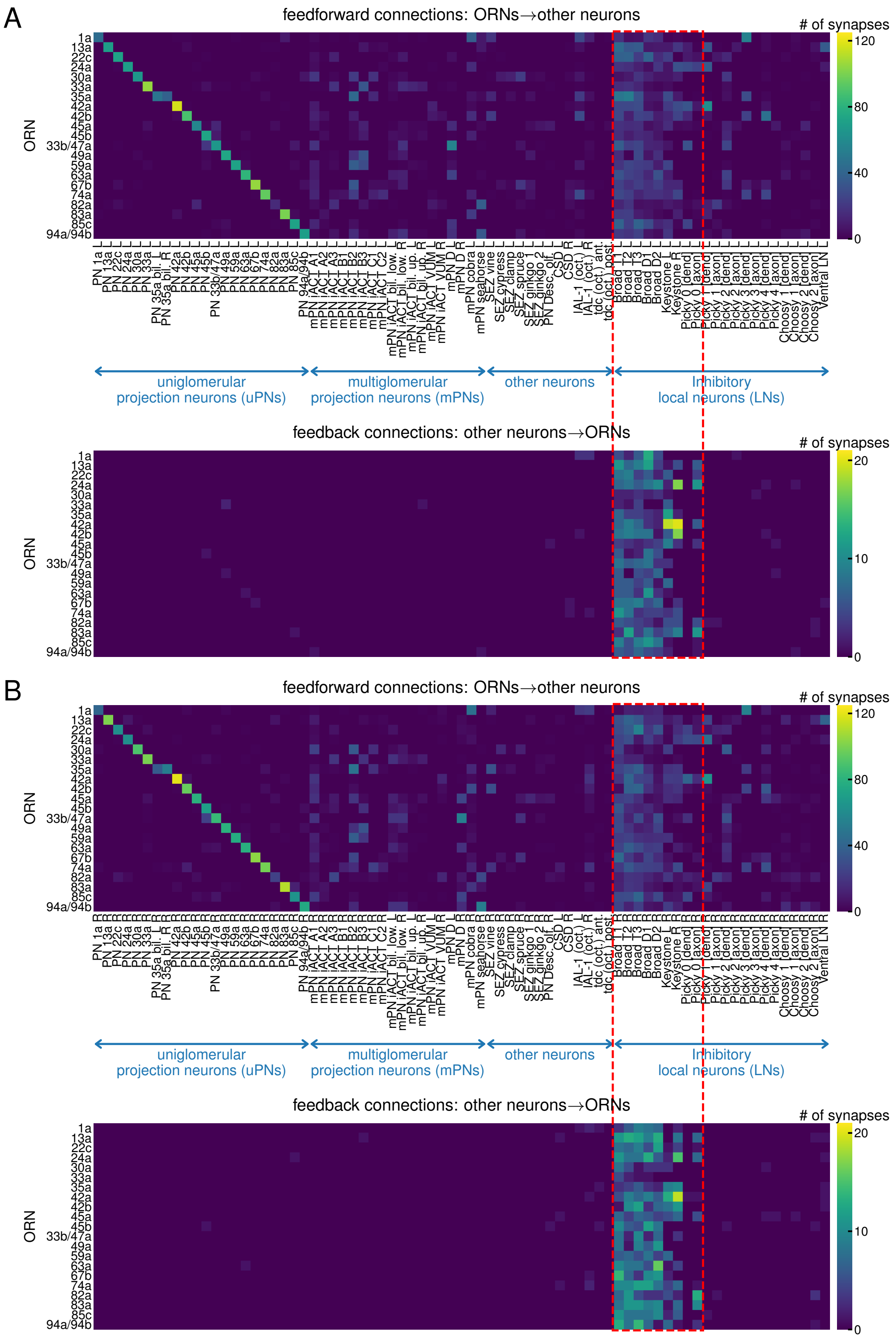

Fig. S1. Full ORN connectivity and circuit selection.
(**A**) Heat map of the ORNs feedforward and feedback connections on the left side of the *Drosophila* larva. We focus on the neurons, that synapse bidirectionally with ORNs (inside the red dashed rectangle): Broad Trios, Broad Duets, Keystones, and Picky 0. These neurons are all LNs.
(**B**) Same as (**A**) for the right side.

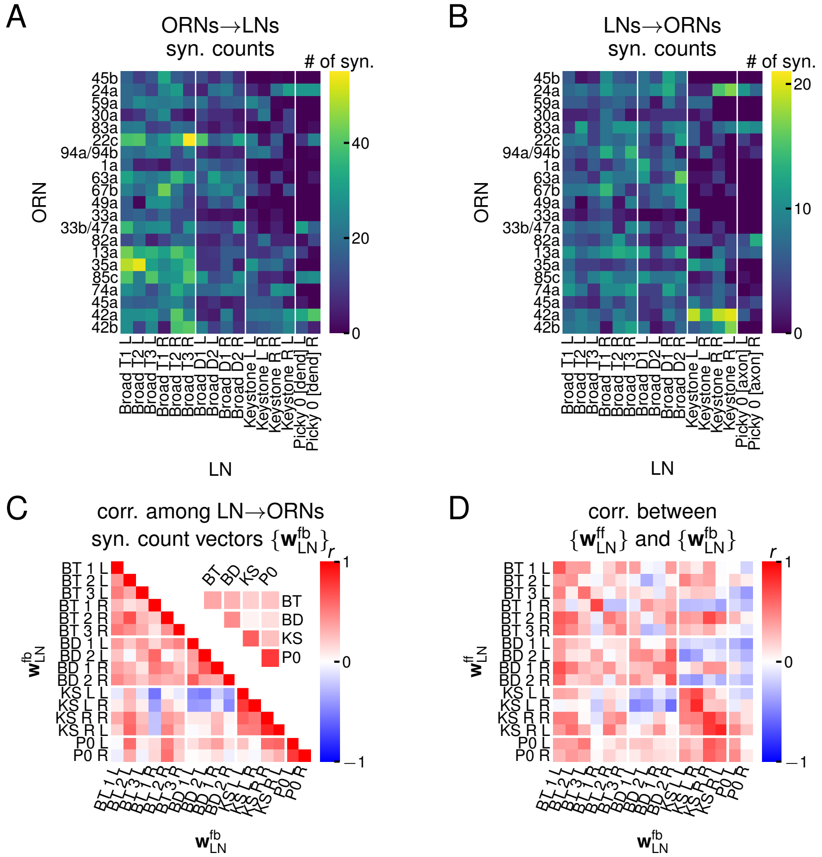

Fig. S2. ORN-LN connectivity, comparison feedforward with feedback.

(**A**) ORNs $\to$ LNs feedforward synaptic counts on both left and right sides of the antennal lobe with the chosen LNs, ordered by LN class. The synaptic count vectors $\mathbf{w}_{\mathrm{LN}}^{\mathrm{ff}}$ correspond to the columns of the depicted matrix.

(**B**) LN $\to$ ORNs feedback synaptic counts $\mathbf{w}_{\mathrm{LN}}^{\mathrm{fb}}$ on both left and right sides of the antennal lobe with the chosen LNs, ordered by LN class. The synaptic count vectors $\mathbf{w}_{\mathrm{LN}}^{\mathrm{fb}}$ correspond to the columns of the depicted matrix.

(**C**) Correlation coefficients between feedback LN $\to$ ORNs synaptic count vectors $\mathbf{w}_{\mathrm{LN}}^{\mathrm{fb}}$. Inset: Average rectified correlation coefficient $\langle r_+ \rangle$ ($r_+ := \max[0, r]$) between LN types calculated by averaging the rectified values from the full matrix in each region with a white border, excluding the diagonal entries of the full matrix. The average correlation coefficient within a class is larger than the correlation coefficient across classes.

(**D**) Correlation coefficients between feedforward ORNs $\to$ LN $\mathbf{w}_{\mathrm{LN}}^{\mathrm{ff}}$ and feedback LN $\to$ ORNs $\mathbf{w}_{\mathrm{LN}}^{\mathrm{fb}}$ synaptic count vectors. The Picky 0 LN is the only LN that has a separation between axonal and dendritic terminals. For the feedforward ORNs $\to$ LN connections, we only include in the synaptic count vector the synapses onto the Picky 0 dendrite, and for the LN $\to$ ORNs connection, we only count the synapses from the Picky 0 axon. Because all the components of the synaptic count vector ORNs $\to$ LN share the same post-synaptic neuron, their effect on the post-synaptic activity is directly comparable and thus the ORNs $\to$ LN synaptic count vector is expected to be largely proportional to the ORNs $\to$ LN synaptic weight vector. However, the synaptic counts from one LN onto all 21 ORNs are not directly comparable to each other, because each connection affects a different postsynaptic ORN, which potentially has different electrical properties. Yet, we see here that the feedforward and feedback connection vectors are somewhat correlated. This justifies the model's property that excitatory feedforward and inhibitory feedback connection weights differ only by a proportionality constant related to ρ^2.

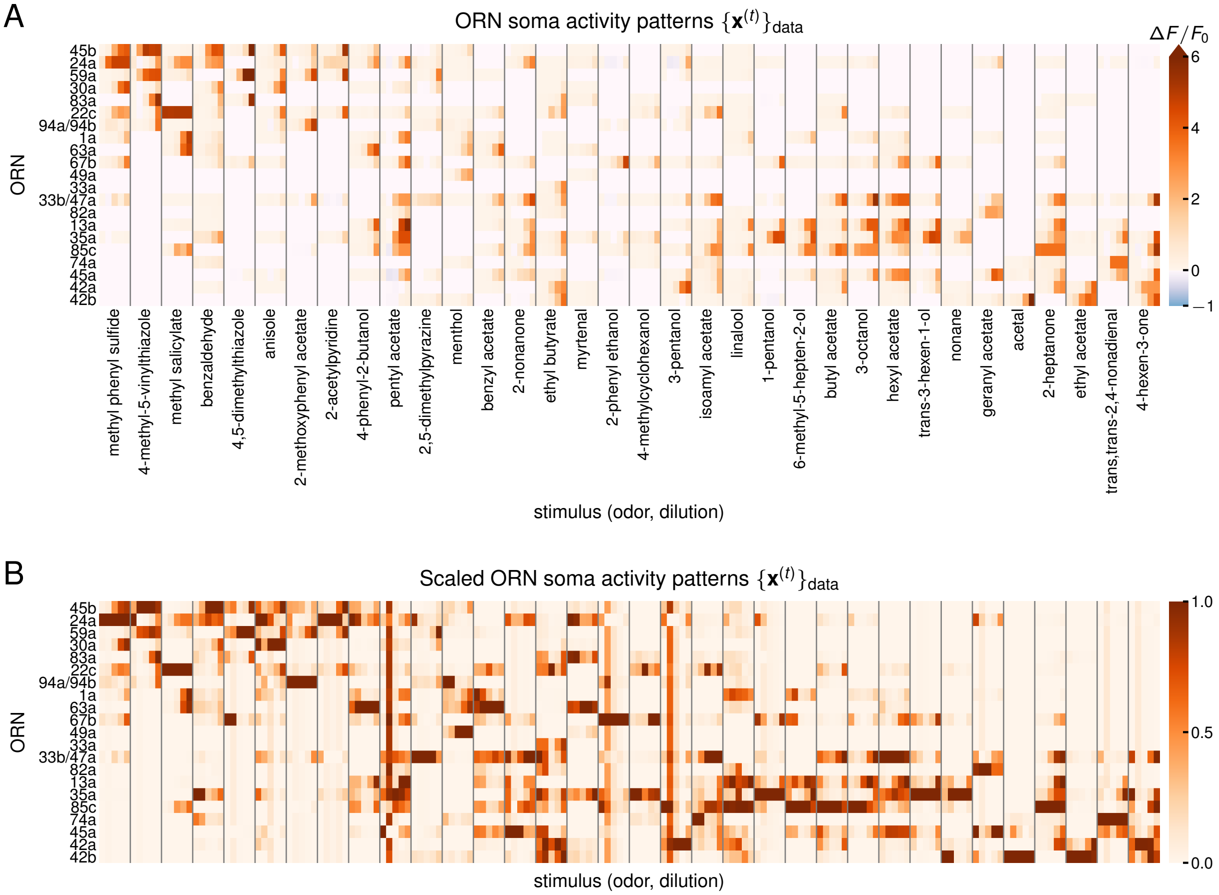

Fig. S3. ORN soma activity from Si et al., 2019(2).

(**A**) ORN soma activity patterns $\{\mathbf{x}^{(t)}\}_{\text{data}}$ in response to 34 odors at 5 dilutions acquired through Ca^{2+} imaging. Different odors are separated by vertical gray lines. For each odor, there are 5 columns corresponding to 5 dilutions: $10^{-8}, \ldots, 10^{-4}$. The odors and ORNs are ordered by the value of the second singular vectors of the left and right SVD matrices of this activity data, after centering and normalizing. This data is obtained by averaging the maximum responses of several trials to the same odor and dilution (as in Si et al., 2019(2)).

(**B**) Same as (**A**), with each $\mathbf{x}^{(t)}$ scaled between 0 and 1 to better portray the patterns.

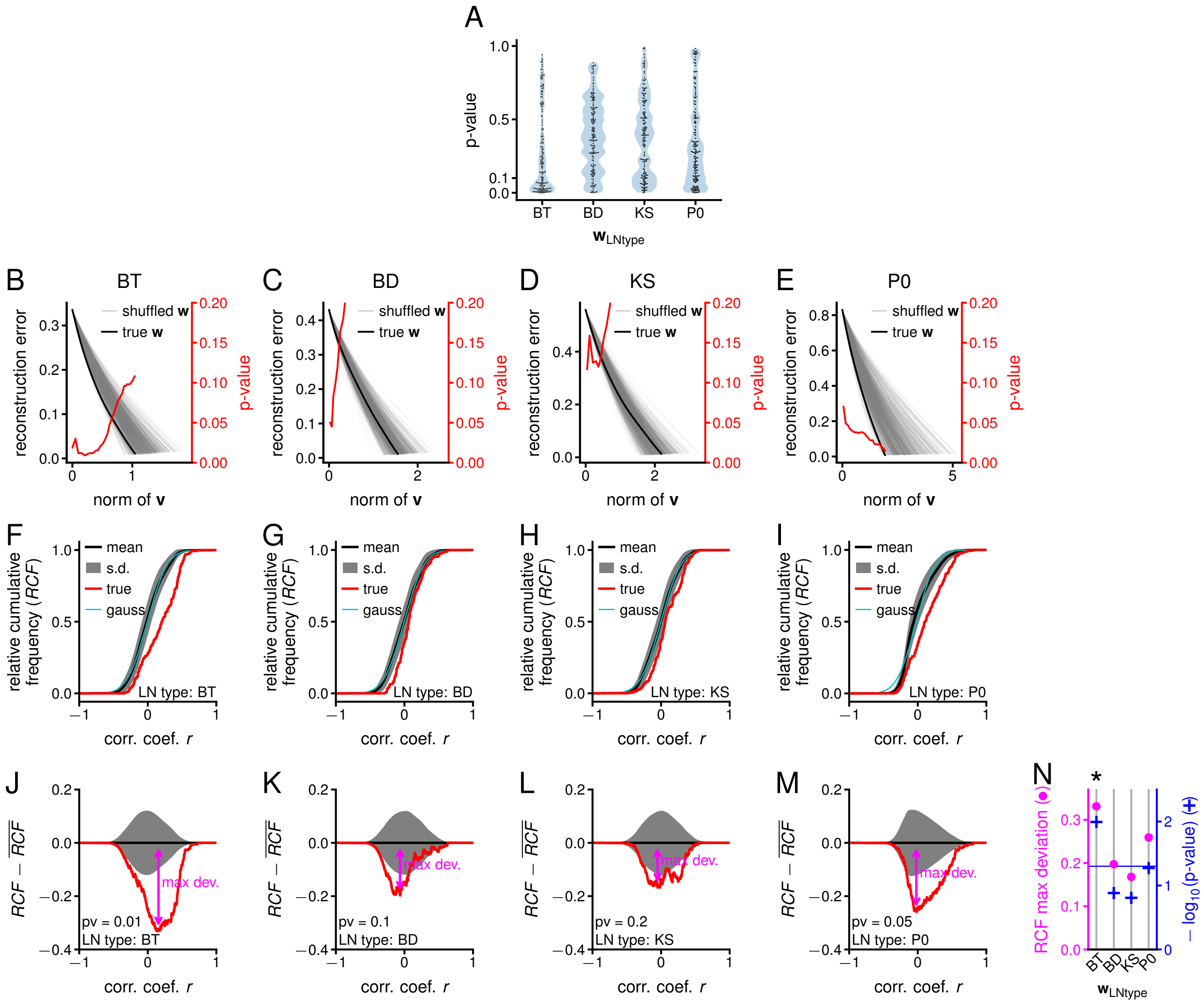

Fig. S4. Alignment of activity patterns $\mathbf{x}^{(t)}$ in ORNs and ORNs $\to$ LN synaptic count vectors $\mathbf{w}_{\text{LNtype}}$.
(**A**) Distribution of p-values arising from the significance testing in Fig. 2B. We observe that for the Broad Trio and Picky 0, the distribution of p-values is skewed towards small values, confirming that the significant correlations found are not solely a result of randomness and multiple comparisons.
(**B-E**) Black line: Reconstruction error $\|\hat{\mathbf{w}}_{\text{LNtype}} - \sum_{t=0}^{T} v_t \hat{\mathbf{x}}^{(t)}\|_2$ as a function of the L1 norm of the coefficient vector $\mathbf{v}$ (see text for details), gray lines: same as black but for a shuffled $\hat{\mathbf{w}}_{\text{LNtype}}$. Red line: proportion of randomly shuffled $\hat{\mathbf{w}}_{\text{LNtype}}$ that have a smaller reconstruction error for the same norm of $\mathbf{v}$. Broad Trio and Picky 0 have significantly better reconstructions as shown by the small p-values for an extended range of $\|\mathbf{v}\|_1$.
(**F-I**) Red line: relative cumulative frequency (RCF) of the correlation coefficients r (from each row of Fig. 2B) between each $\mathbf{w}_{\text{LNtype}}$ and all the $\{\mathbf{x}^{(t)}\}_{\text{data}}$. In other words, the RCF in a normalized cumulative histogram of all the correlation coefficients in one row of Fig. 2B. Black line and gray band: mean $\pm$ SD from the RCFs generated by 50,000 instances of shuffling the entries of $\mathbf{w}_{\text{BT}}$. Blue line: normal fit to the shuffled distribution. Apart from the LN type P0, the distribution arising from shuffling is quite close to normal. This can be explained by the fact that P0 has sparse connectivity. Bin size: 0.004.
(**J-M**) Same as (**F-I**) with the mean RCF subtracted. We define the maximum deviation as the maximum negative difference between the true and the mean RCF of correlation coefficients.
(**N**) RCF maximum deviation and $\log_{10}$ of the multi-comparison adjusted p-values (9) for each of the four ORNs $\to$ LN$_{\text{type}}$ synaptic count vectors $\mathbf{w}_{\text{LNtype}}$. *: significance at 5% FDR (false discovery rate).

A

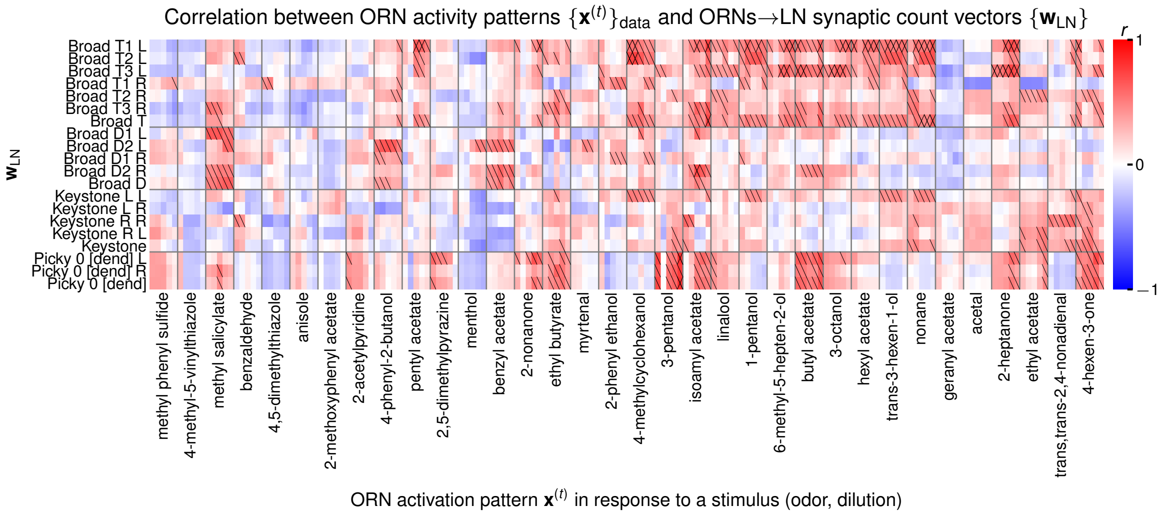

Fig. S5. Alignment of activity patterns $\mathbf{x}^{(t)}$ in ORNs and ORNs → LN synaptic count vectors $\mathbf{w}_{\mathbf{LN}}$.
(**A**) Same as Fig. 2B, for all the $\mathbf{w}_{\text{LN}}$ and $\mathbf{w}_{\text{LNtype}}$ and with all the odors labeled. The label "Broad T" corresponds to the average ORNs → LN synaptic count vector for all Broad Trio LNs; same for "Broad D", "Keystone", and "Picky 0 [dend]". These correspond to the ones shown in Fig. 2B. The individual LNs have similar correlation patterns as the average ones. Same odor order.

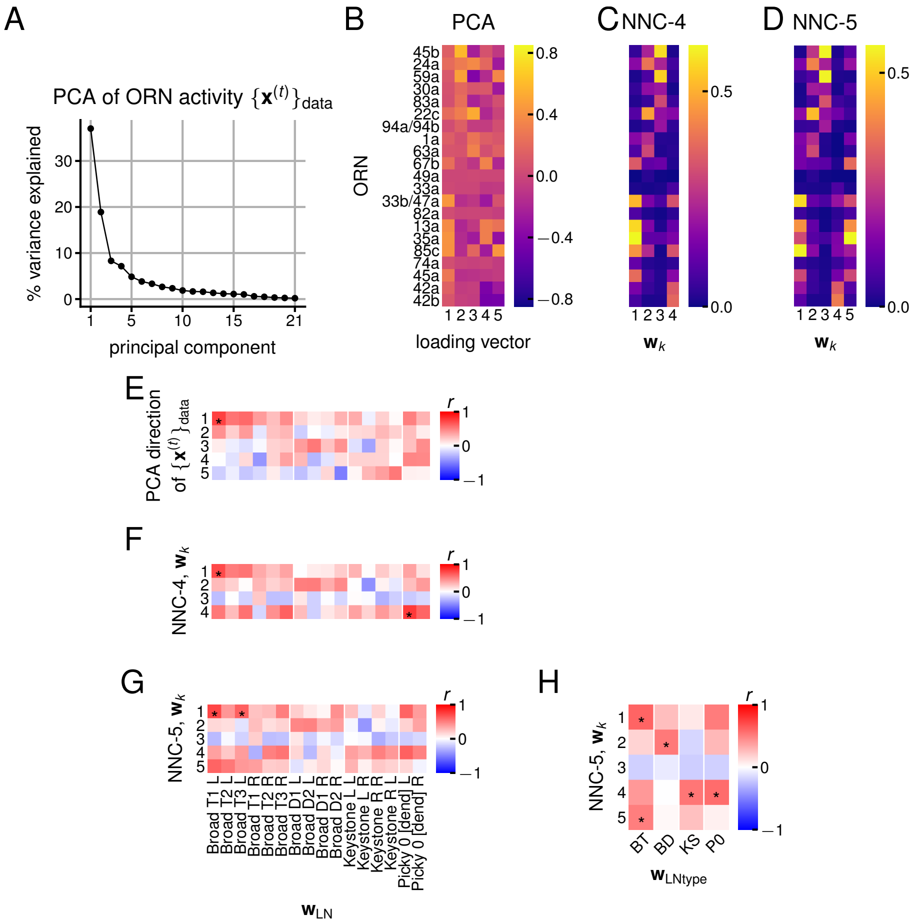

Fig. S6. PCA of ORN activity and NNC connectivity vs data connectivity.
(**A**) Percentage of the variance of the ORN activity patters $\{\mathbf{x}^{(t)}\}_{\text{data}}$ explained by the uncentered PCA. The top 4 and 5 PCA directions explain 71% and 76% of the variance, respectively.
(**B**) First 5 PCA loading vectors of $\{\mathbf{x}^{(t)}\}_{\text{data}}$.
(**C-D**) $\mathbf{w}_k$ from NNC with $K = 4, 5$ and $\rho = 1$.
(**E**) Same as Fig. 2H with all $\mathbf{w}_{\text{LN}}$.
(**F**) Same as (**E**), with $\mathbf{w}_k$ from NNC-4 instead of PCA directions.
(**G**) Same as (**F**), for NNC-5. The small number of significant points in (**E-G**) results from the higher number of hypothesis tests, which decreases the adjusted p-values in the FDR multi-hypothesis testing framework.
(**H**) Same as Fig. 3A, for NNC-5.

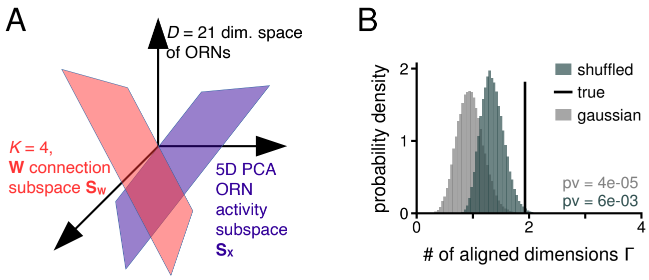

Fig. S7. Activity and connectivity subspace alignment.
(**A**) Schematic representing the comparison of the 4-dimensional connectivity (S_W) and 5-dimensional activity (S_X) subspaces in 21 dimensions ($D = 21$, dimensionality of the ORN space).
(**B**) Number of aligned dimensions Γ between the 2 subspaces of (**A**) in the data (true, $\Gamma = 1.9$), from randomly shuffling the connectivity vector entries (shuffled, mean $\Gamma = 1.3$) and from random normal vectors (Gaussian, mean $\Gamma = 1$). About one dimension is more aligned in the data than expected by random. pv: one-sided p-value.

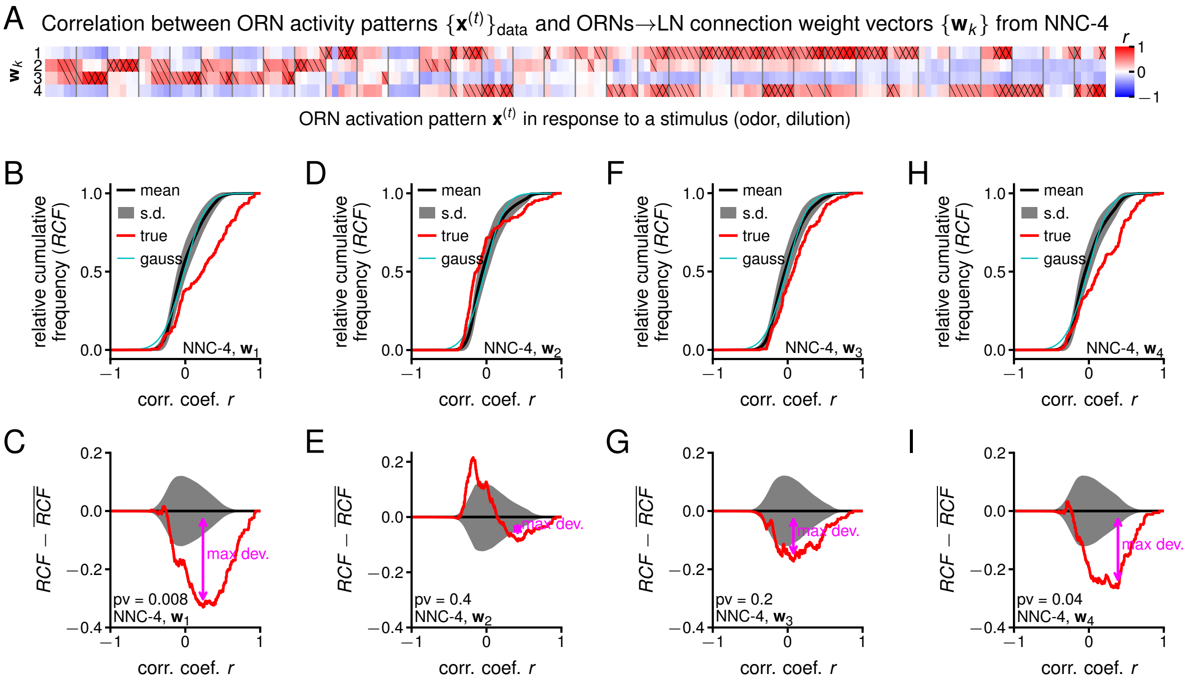

Fig. S8. Alignment of activity patterns $\{\mathrm{x}^{(t)}\}_{\text{data}}$ in ORNs and connectivity weight vectors $\{\mathrm{w}_k\}$ from NNC-4.
(**A**) Same as Fig. 2B, for the four ORNs $\rightarrow$ LN connection weight vectors $\mathbf{w}_k$ arising from NNC-4 simulations ($\rho = 1$). We see that the LNs of the NNC model, which is specifically adapted to this set of odors, have high and significant correlations with different sets of odors. $\mathbf{w}_1$ most resemble $\mathbf{w}_{\text{BT}}$, $\mathbf{w}_2$ - $\mathbf{w}_{\text{BD}}$, and $\mathbf{w}_4$ - $\mathbf{w}_{\text{P0}}$.
(**B-I**) Same as Figs. S4F to M, for the four $\mathbf{w}_k$ arising from NNC-4 simulations and with an overlaid normal fit to the shuffled distribution. These plots are quite similar to the ones based on the connectome, showing an additional match between the model and experimental data. In particular, we find two connectivity vectors ($\mathbf{w}_1$ and $\mathbf{w}_4$) that have, just as BT and P0, rather large deviations from the shuffled distribution, and the other two, just as BD and KS, are closer to the shuffled distribution.

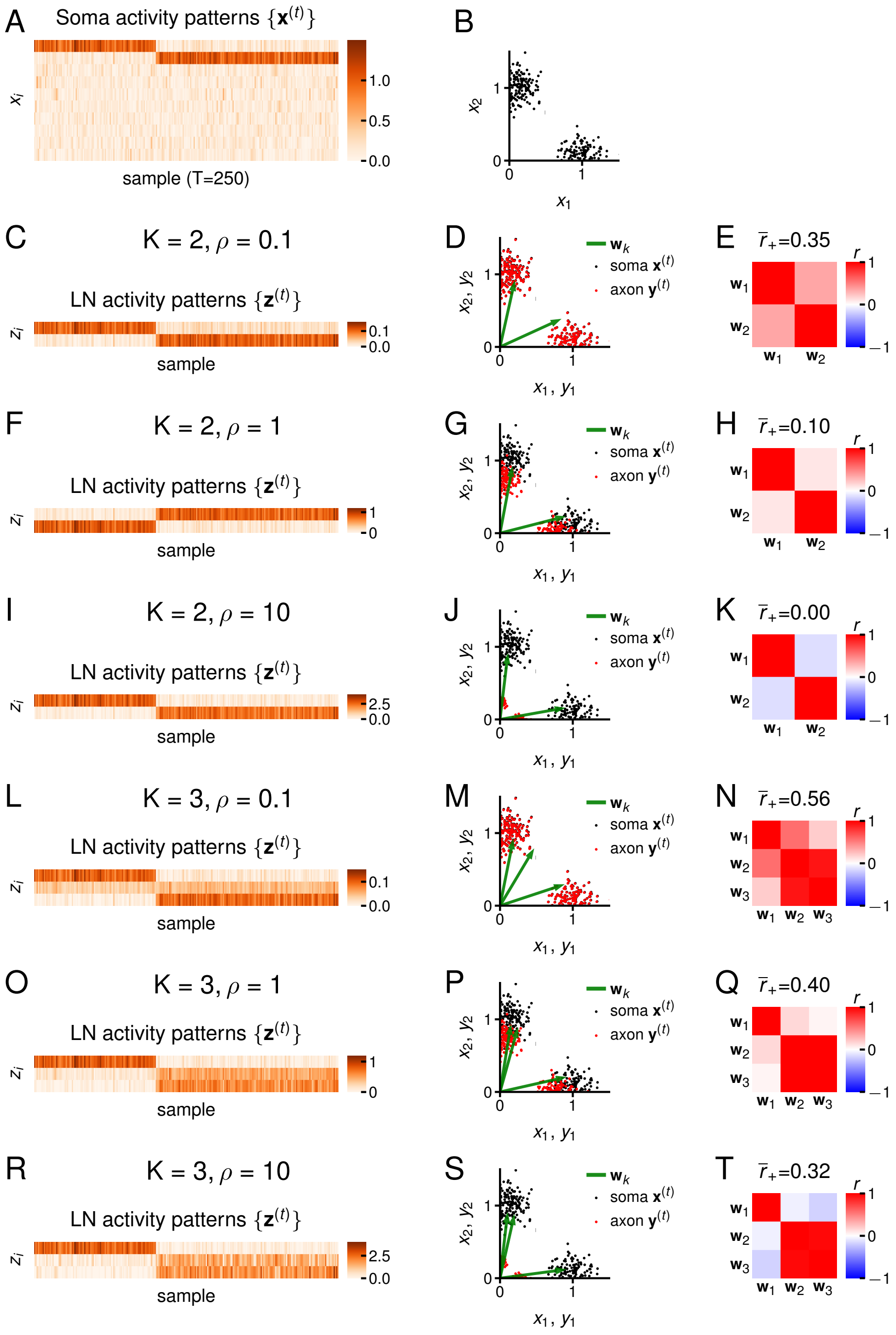

Fig. S9. Clustering by the NNC and correlation between the $\mathbf{w}_k$ for two separated clusters.
In this and the next figures, we investigate the effect of varying the input statistics $\{\mathbf{x}^{(t)}\}$, ρ (encoding the ratio between the feedforward and feedback connectivity strength), and K (the number of LNs) on the alignment between the $\{\mathbf{w}_k\}$ arising in the NNC model. In both figures, the input dataset $\{\mathbf{x}^{(t)}\}$ is in $D = 10$ dimensions and contains 250 sample points spread between 2 clusters (one of 100 points and the other of 150 points). Points were generated using a normal distribution of SD = 0.15. The absolute value was taken in each coordinate. In this figure, the 2 clusters are further apart than in the next figure, which probes the effect of changing the statistics of $\{\mathbf{x}^{(t)}\}$. In both figures we consider the case with $K = 2$ and $K = 3$, and ρ taking values $0.1, 1, 10$.
(**A**) Representation of $\{\mathbf{x}^{(t)}\}$. For the first 100 points, the normal distribution was centered at [1, 0, ...,0]; for the last 150 points, the normal distribution was centered at [0, 1, 0, ..., 0].
(**B**) Scatter plot of the first 2 dimensions of the dataset of (**A**).
(**C-T**) Output of the NNC model trained on the dataset in (**A**) for $K = 2, 3$ and for $\rho = 0.1, 1, 10$.
(caption continues on next page)

Fig. S9. Clustering by the NNC and correlation between the $\mathbf{w}_k$ for two separated clusters, continued.
(**C, F, I, L, O, R**) LN activity. As ρ increases, the activity of LNs increases in amplitude, leading to a stronger inhibition. In most cases, LN activity clearly encodes the membership of an input sample to a cluster. For $K = 3$, 2 LNs encode the membership of the cluster with more points. With stronger ρ, the 2 LNs encoding the same cluster become more similar.
(**D, G, J, M, P, S**) Scatter plot of the input $\{\mathbf{x}^{(t)}\}$ (black), output $\{\mathbf{y}^{(t)}\}$ (red), and direction of the $\mathbf{w}_k$ (green) in the first two dimensions. As ρ increases, the output becomes smaller due to a stronger inhibition, and the $\{\mathbf{w}_k\}$ become more separated, especially for $K = 2$. For $K = 3$, two $\mathbf{w}_k$ point towards the cluster with more points.
(**E, H, K, N, Q, T**) Correlation coefficients between $\mathbf{w}_k$ and mean rectified correlation coefficient $\bar{r}_+$. As ρ increases, the $\mathbf{w}_k$ describing different clusters become more decorrelated and $\bar{r}_+$ decreases. For the case when $K = 3$ and there are only 2 clusters in the dataset, two $\mathbf{w}_k$ stay correlated for even large values of ρ.

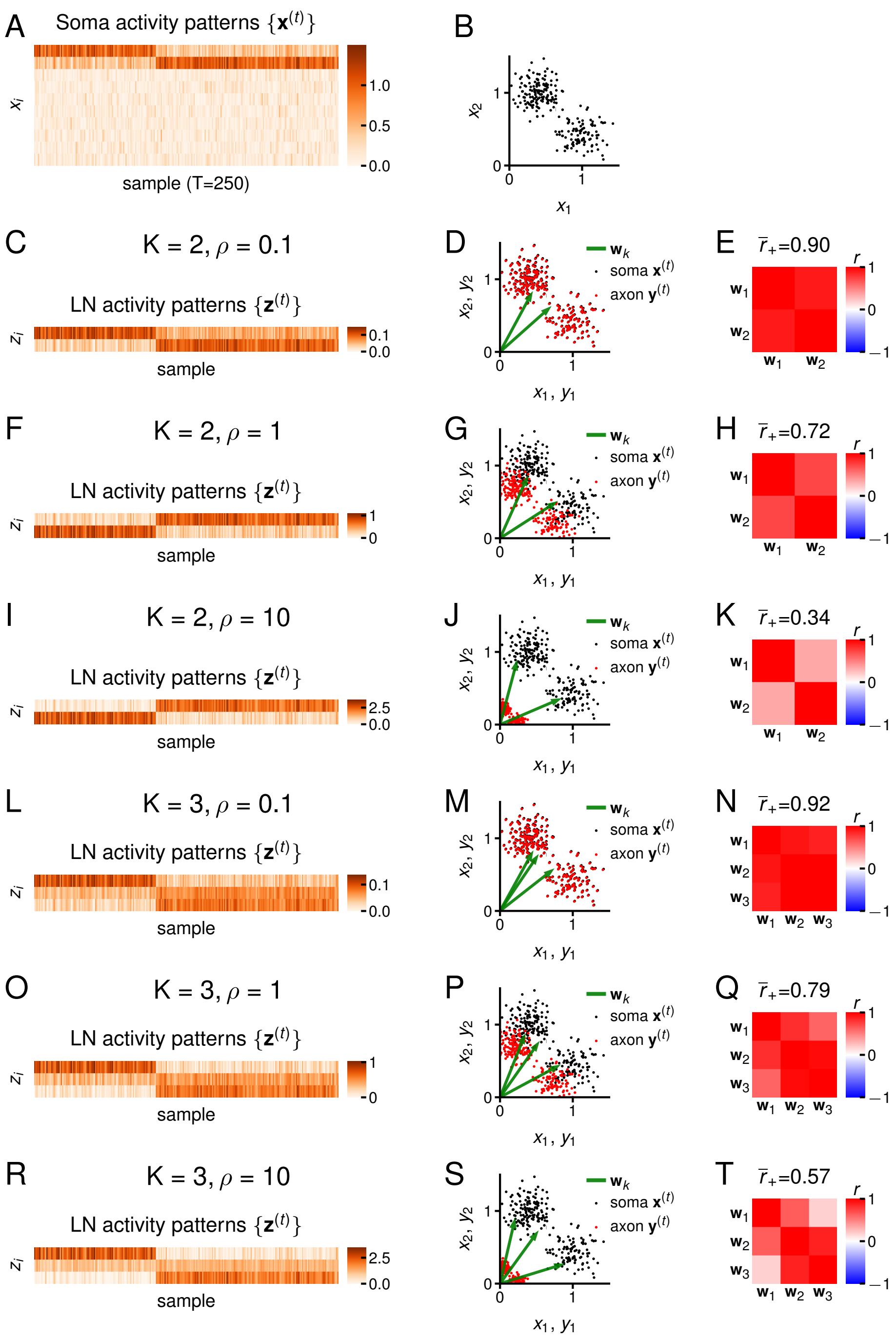

Fig. S10. Clustering by the NNC and correlation between the $\mathbf{w}_k$ for two nearby clusters.
Same as Fig. S9 but when the two clusters are closer together. For the first 100 points, the normal distribution was centered at [1, 0.4, 0, ..., 0]; for the last 150 points, the normal distribution was centered at [0.4, 1, 0, ..., 0]. One finds that for small ρ, even though the $\mathbf{w}_k$ are very correlated, at least two LNs successfully encode the cluster membership. However, increasing ρ improves the cluster separation: the angle between the black clusters is smaller than the angle between the red clusters. Finally, when $K = 3$, we observe that one of the LNs does not always take the side of one cluster. Because of the difference in the dataset with Fig. S9, the mean rectified correlation coefficient $\bar{r}_+$ between the $\mathbf{w}_k$ is always larger for this dataset, thus demonstrating how the dataset influences the $\mathbf{w}_k$.

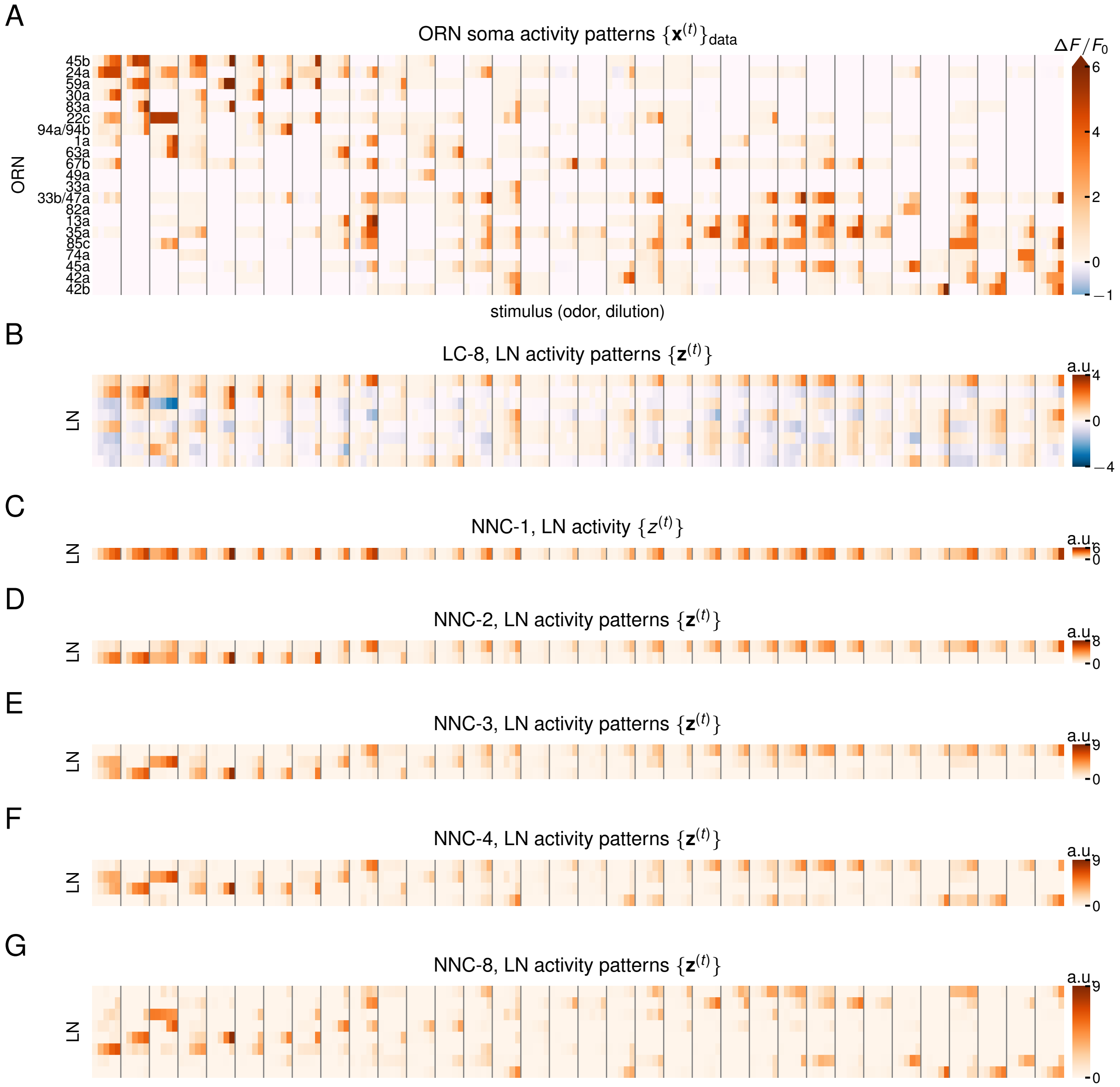

Fig. S11. Activity of LNs $\{\mathbf{z}^{(t)}\}$ in the NNC and LC.
(**A**) ORN soma activity patterns $\{\mathbf{x}^{(t)}\}_{\text{data}}$ as in Fig. S3A, replicated for convenience.
(**B**) Activity in the LNs $\{\mathbf{z}^{(t)}\}$ for the LC-8. Stimuli are aligned to the panel above. As mentioned in the text, $\{\mathbf{z}^{(t)}\}$ is undetermined up to an orthogonal matrix $\mathbf{U}_Z$. Here we set $\mathbf{U}_Z = \mathbf{I}_K$, i.e., the identity matrix. This special case corresponds to the situation where each LN encodes a PCA direction of ORN activity. For LC-K with $K \leq 8$, the response in LNs corresponds to the first K row of this matrix, multiplied by any $K \times K$ orthogonal matrix on the left. Thus, the matrix depicted in this plot shows the potential activity in LNs for any LC-K with $K \leq 8$.
(**C**) $\{z_t\}$ for the NNC-1. The activity of the LN approximately follows the total activity.
(**D**) $\{\mathbf{z}^{(t)}\}$ for the NNC-2. One can see that the 2 LNs roughly cluster the sets of odors into those activating the top ORNs and those activating the lower ORNs.
(**E-G**) $\{\mathbf{z}^{(t)}\}$ for the NNC with $K = 3, 4, 8$. One observes a more sophisticated clustering of the data. As more LNs are added, LN activity increases in sparsity. LNs are mostly active in response to the odors to which their connectivity is the most aligned (NNC-4, Fig. S8A). The activity in the LNs for the NNC is more sparse than for the LC.

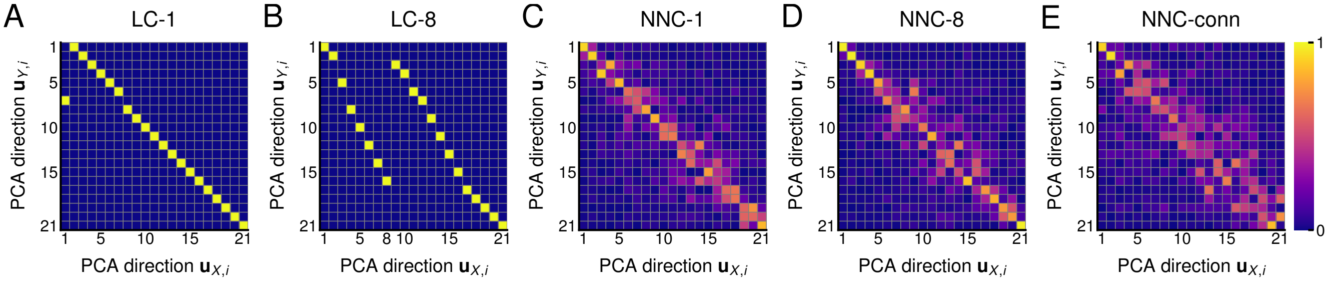

Fig. S12. PCA directions of odor representations at ORN somas vs ORN axons in LC and NNC.
This figure complements Fig. 6 to characterize the difference in PCA directions of the odor representations at ORN somas ($\{\mathbf{x}^{(t)}\}_{\text{data}}$) vs. at ORN axons ($\{\mathbf{y}^{(t)}\}$) in the LC, NNC, and NNC-conn models. We consider models LC-1, LC-8, NNC-1, NNC-8, NNC-conn, i.e., LC and NNC models with $K = 1$ and $K = 8$ LNs, as well as the NNC model constructed based on the synaptic counts in the connectome. $\{\mathbf{u}_{X,i}\}$ and $\{\mathbf{u}_{Y,i}\}$ are the PCA directions of the uncentered activity at the somas ($\{\mathbf{x}^{(t)}\}_{\text{data}}$) and axons ($\{\mathbf{y}^{(t)}\}$), respectively. There are $D = 21$ PCA directions (as the number of ORNs). To quantify the change of PCA directions, we calculate the scalar products between $\{\mathbf{u}_{X,i}\}$ and $\{\mathbf{u}_{Y,i}\}$. A scalar product of 1 (or -1), means that the direction is exactly the same; 0: means that they are perpendicular. Because PCA direction vectors are determined up to the sign, we show the absolute value of the scalar product.
Change of PCA directions has implications on the stimuli representations. If the PCA directions are strongly altered, it could mean the cloud of representation in the neural space is not only stretched but also rotated. Having a minimal rotation of the representations is potentially advantageous for downstream processing, because, since the ORN axon representation is computed dynamically through LN activation, the original representation appearing in ORN axons before the effect of LNs kick in will be maximally close to the final, converged representation. Thus downstream processing can be meaningful even before representation convergence. A lack of rotation is called a “zero-phase”. If the rotation of the stimulus was substantial between the original representation at the ORN soma and the converged representation at ORN axons, the downstream computation could potentially be wasted at stimulus presentation and give incorrect information to the brain about stimulus identity.
(**A-B**) LC-1 and LC-8. For the LC, the identity of the PCA directions is conserved, only their order changes, as can be deduced from the fact that all scalar products between $\{\mathbf{u}_{X,i}\}$ and $\{\mathbf{u}_{Y,i}\}$ are either 1 or 0. Because the variance of the first or first 8 PCA directions decreases, their global order change.
(**C-D**) NNC-1, NNC-8. For the NNC, the PCA directions at the soma and at the axon are not exactly the same, but they conserve their approximate ordering.
(**E**) NNC-conn model. Here, the PCA directions are even more intermixed than in the NNC-8 model, similar to NNC’-8 model (Fig. S17), where the LN-LN connection have been removed.

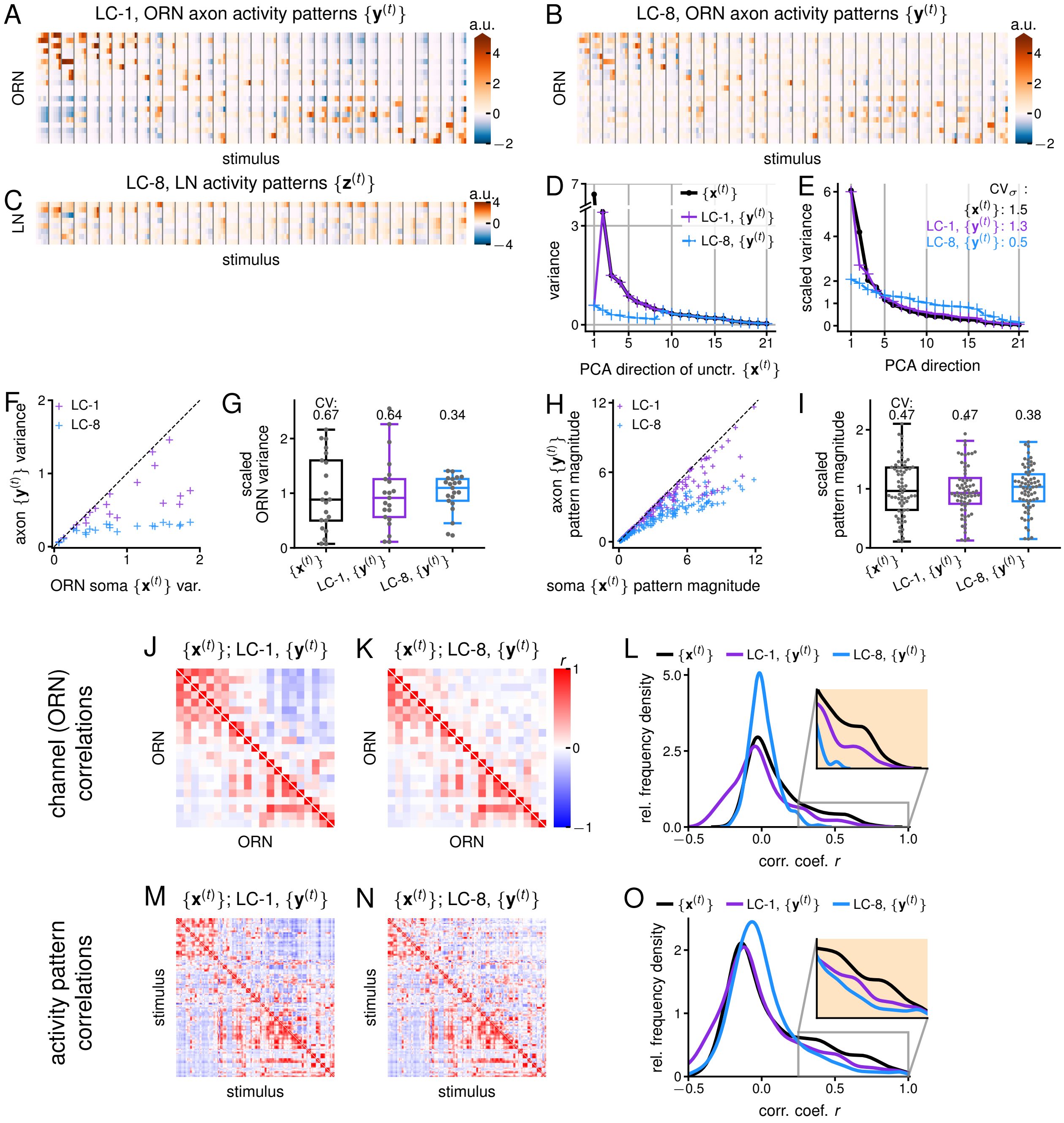

Fig. S13. Input transformation by LC-1 and LC-8 with $\rho = 2$.

This figure complements Fig. 6 to comprehensively show the computation of the LC-1 ($K = 1$ LN) and LC-8 ($K = 8$ LNs) models. Some of the plots are repeated here for convenience.

(**A-B**) ORN axon activity for the LC-1 and LC-8. Corresponds to Fig. 6B. The LC produce negative values and the LC-1 has much more negative deviations.

(**C**) LN activity in the LC-8. Repetition of Fig. S11B, shown here for convenience.

(**D-E**) Corresponds to Figs. 6C and D. For the LC-1, only the first PCA direction is dampened, thus the decrease is CV_σ is not as large as for the LC-8.

(**F-I**) Corresponds to Figs. 6E to H. Again here, the LC-1 does not exhibit much decrease in the CV of ORN variance, and no decrease in the CV of the pattern magnitude. Thus, for this dataset, multiple LNs are necessary in the LC model to have the effect of normalization.

(**J-O**) Corresponds to Figs. 6I to L. Although present, the decorrelation in the LC-1 is not as strong as in LC-8. LC-1 produces more negatively correlated ORNs and activity patterns.

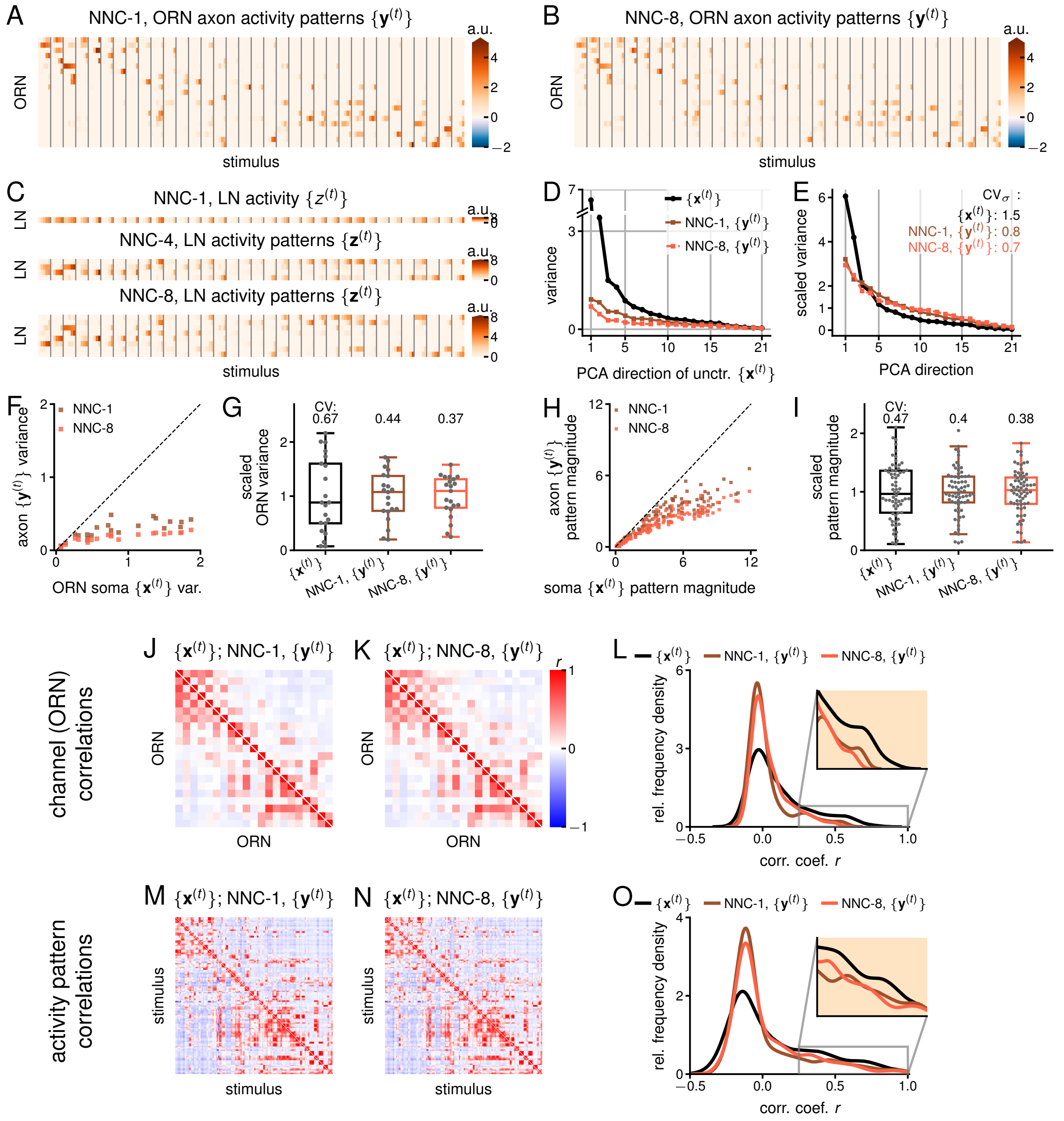

Fig. S14. Input transformation by NNC-1 and NNC-8 with $\rho = 2$.
This figure complements Fig. 6 to comprehensively show the computation of the NNC-1 ($K = 1$ LN) and NNC-8 ($K = 8$ LNs) models. The structure of the figure is the same as in Fig. S13. Some of the plots are repeated here for convenience.
(**A-B**) ORN axon activity for the NNC-1 and NNC-8. Corresponds to Fig. 6B. The ORN axon activity in the NNC-1 is comparable, but a bit stronger than in NNC-8. This is due to a weaker overall inhibition in LC-1. But note that the parameter ρ also contributes to the inhibition strength.
(**C**) LN activity in the NNC model with $K = 1, 4$ and 8. Repetition of Fig. S11B, shown here for convenience.
(**D-O**) Corresponds to Figs. 6C to L and Figs. S13D to O. Contrary to LC-1 and LC-8 that are quite different, generally NNC-1 and NNC-8 are quite similar. As for $K = 8$, in $K = 1$ the variances of all PCA directions are decreased. This contrasts to the LC, where only the variances of the top K PCA directions are affected. The NNC-1 exhibits a weaker normalization of ORN variance and pattern magnitude, but almost no different in terms of decorrelation. This differs from the LC, where there the decorrelation in the LC-8 is perceptibly stronger than in the LC-1.

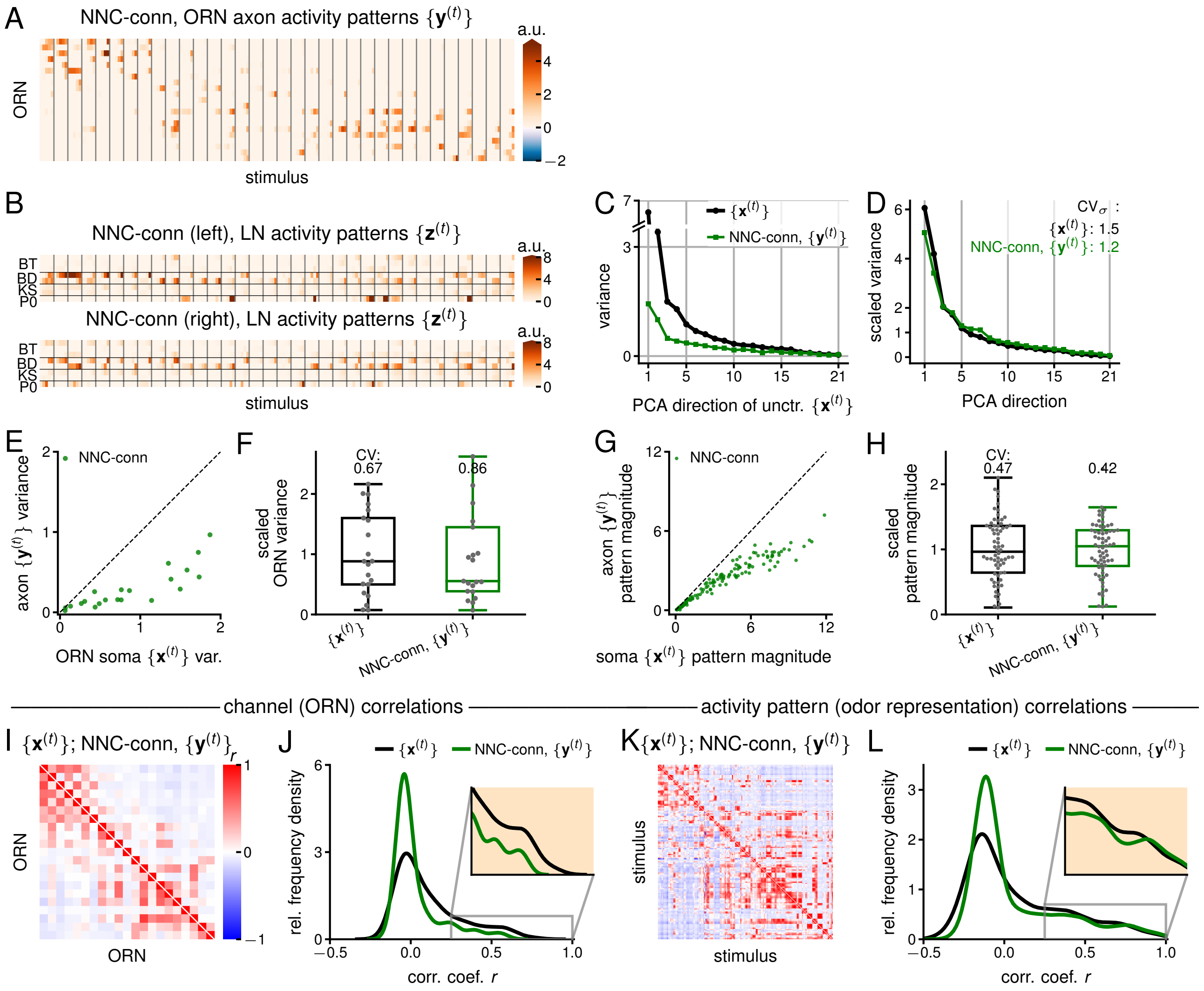

Fig. S15. Input transformation by a nonnegative circuit with synaptic weight vectors from the connectome.
This figure complements Fig. 6 and repeats some plots for convenience. See *SI Appendix*, Section 15 for implementation details. In this circuit, synaptic weights are set proportionally to the synaptic counts from the connectome (1) and we call this model NNC-conn. As a whole, apart from the increase in the CV of ORN variance, this model performs a qualitatively comparable computation to the one by NNC and LC models.
(**A**) ORN axon activity in the NNC-conn. Corresponds to Fig. 6B. This is the average activity between left and right sides. The activity in ORN axons is nonnegative and weaker than in ORN soma, as seen in the NNC model.
(**B**) LN activity in the NNC-conn. Corresponds to Fig. 6A. Showing the activity on both left and right sides. The activity in LNs is rather sparse and distributed, as in the NNC. The first three rows are the activities in the Broad Trio 1, 2, and 3 (BT); rows 4 and 5: Broad Duet 1 and 2 (BD); rows 6 and 7: Keystone L and Keystone R (KS); row 8: Picky 0 (P0). The horizontal black lines separate the 4 LN types. One observes a stronger activity in the Broad Duets. Given the uncertainty of the parameters of the model, we do not know if it is true in reality, or just a consequence of incorrect synaptic weights or leak parameters.
(**C-D**) Repeated Figs. 6C and D. In the NNC-conn the first 2 PCA directions are not as strongly dampened as in the NNC and LC, leading to a lesser decrease in the spread of the PCA variances.
(**E-H**) Repeated Figs. 6E to H. In the NNC-conn the first 2 PCA directions are not as strongly dampened as in the NNC and LC.
(**I-J**) Corresponds to Figs. 6I and J. The channels are more decorrelated at the ORN axons than at the somas as seen in the NNC and LC models.
(**K-L**) Corresponds to Figs. 6K and L. The odor representations are slightly more decorrelated at the ORN axons than at the somas in the histograms. This effect is weaker here than in the NNC model.

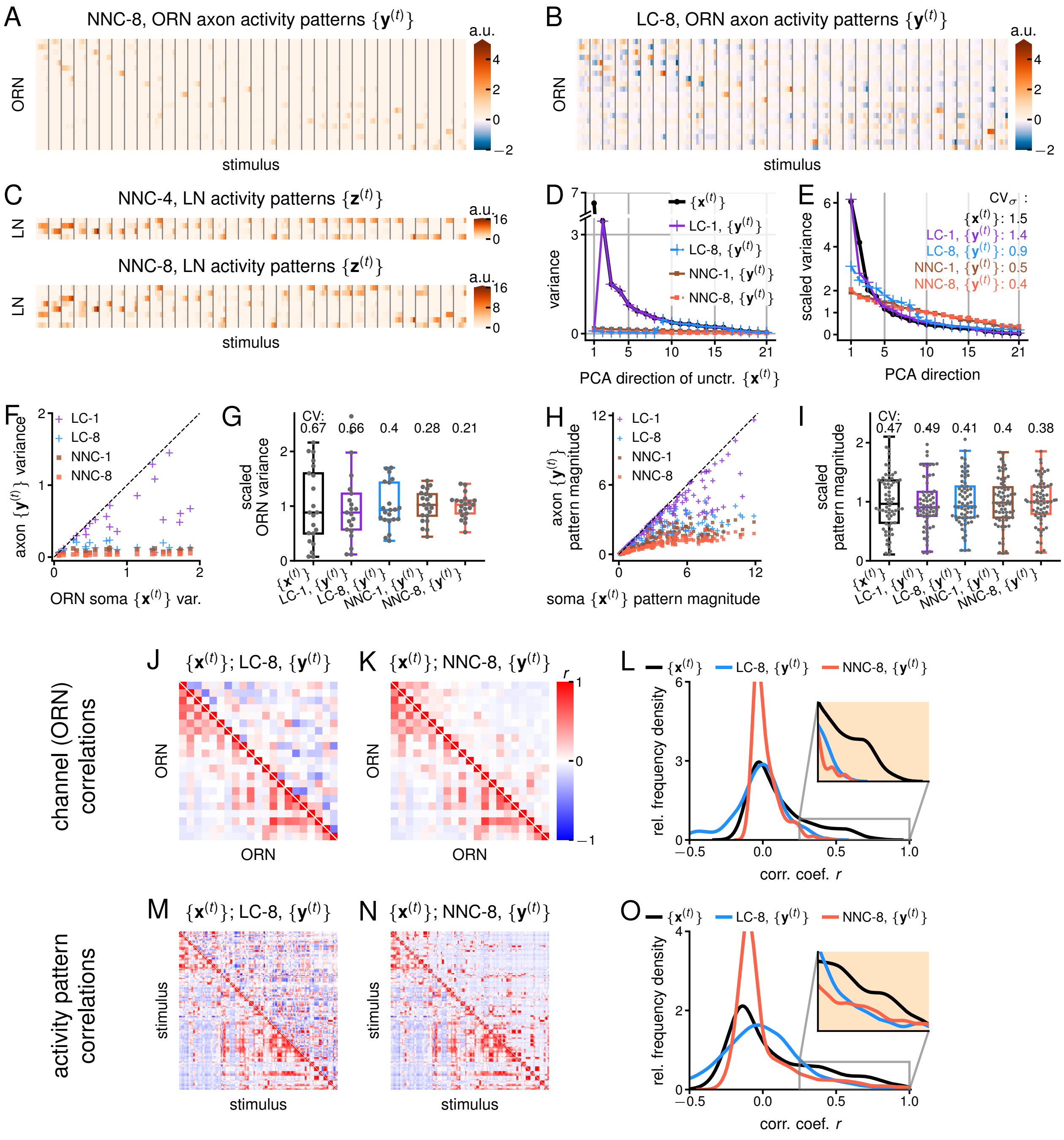

Fig. S16. Input transformation by LC and NNC with $\rho = 10$.
This figure complements the findings of Fig. 6. To better understand the effect of a stronger inhibition in the LC and NNC models we perform the same analysis as in Fig. 6 with $\rho = 10$. This setting gives a perceptively strong effect on the output and allows us to understand the effect of changing ρ. In general, we observe an even stronger dampening, flattening, and decorrelation than for $\rho = 2$.
(**A-C**) Corresponds to Figs. 6A and B. The activity for the LC and NNC at the axonal level is even weaker than for $\rho = 2$. For the LC the negative values are more perceptible and there are more values around 0. The activity in LNs is stronger.
(**D**) Corresponds to Fig. 6C. For the LC models, the PCA directions that are affected by LN inhibitions have an even smaller variance. For the NNC models, all directions are even smaller.
(**E**) Corresponds to Fig. 6D. The spread of variances (quantified by the CV_σ) is slightly bigger for the LC at $\rho = 10$ than at $\rho = 2$, because only the K first variances are even smaller, which increases the overall spread of variances in this situation. For the NNC however, because all directions are dampened, the CV is smaller here than for $\rho = 2$. (caption continues on next page)

Fig. S16. Input transformation by LC and NNC with $\rho = 10$, continued.
(**F-I**) Corresponds to Figs. 6E to H. Because LC-1 only affects a single PCA direction, the results for $\rho = 2$ and $\rho = 10$ are quite similar in terms of channel variance and pattern magnitudes for this model. For LC-8, although we observe a decrease in channel variances and pattern magnitudes, there is virtually no difference between $\rho = 2$ and $\rho = 10$ in terms of the CV of channel variances or pattern magnitudes. For the NNC models, we observe both a decrease in channel variances and pattern magnitudes, and a decrease in their CV in comparison to when $\rho = 2$. As for (**E**), the difference between LC and NNC can be attributed to the fact that LC only affects certain stimulus directions, whereas the NNC as a global effect.
(**J-L**) Corresponds to Figs. 6I and J, Figs. S13K and L, and Figs. S14K and L. At $\rho = 10$, the channels are even more decorrelated than at $\rho = 10$ as seen in the correlation matrices and the histograms. For the LC, some channels become anti-correlated.
(**M-O**) Corresponds to Figs. 6K and L, Figs. S13N and O, and Figs. S14N and O. At $\rho = 10$, the odor representations are even more decorrelated than at $\rho = 2$ as seen in the correlation matrices and the histograms. This can particularly be observed for correlation coefficients above 0.5, whose proportion is less than at $\rho = 2$.

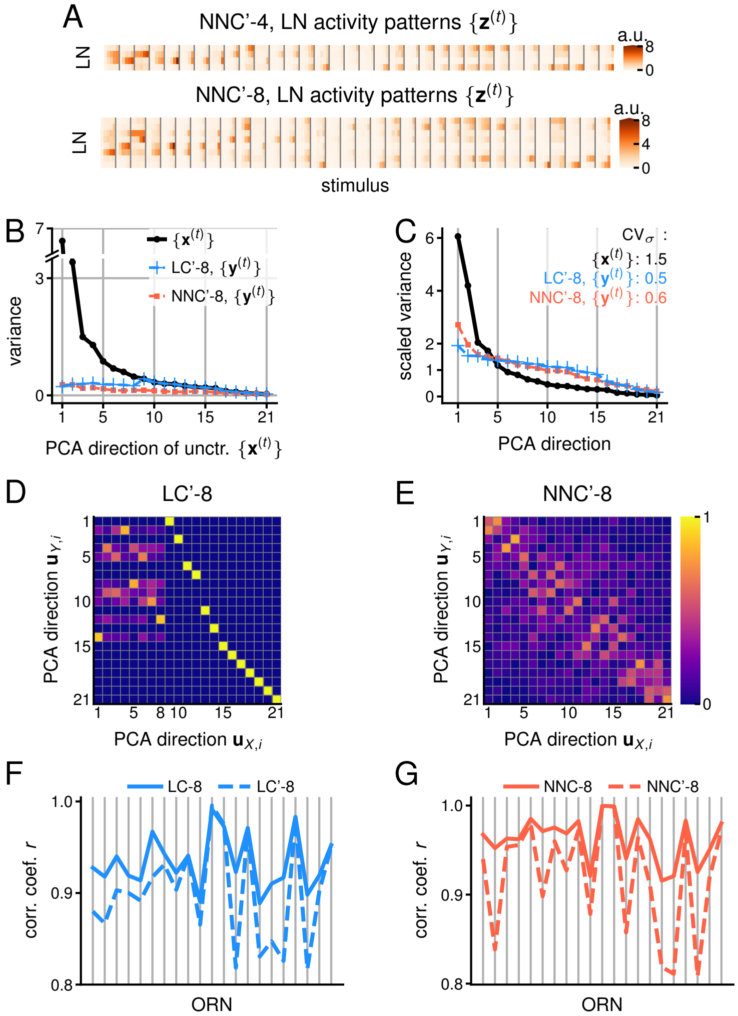

Fig. S17. Effect of removing LN-LN connections on the LC and NNC model.
We call LC' and NNC' the circuit models LC and NNC, which we trained on odor representations $\{\mathbf{x}^{(t)}\}_{\text{data}}$, and which had subsequently their LN-LN connections removed. This corresponds to setting off-diagonal values of $\mathbf{M}$ to 0. As mentioned in the text, for the LC, $\{\mathbf{z}^{(t)}\}$ is undetermined up to an orthogonal matrix $\mathbf{U}_Z$. Here we set $\mathbf{U}_Z \neq \mathbf{I}_K$. If $\mathbf{U}_Z = \mathbf{I}_K$, the off-diagonal values of $\mathbf{M}$ are already 0 (Eq. (S104)), and thus this manipulation has no effect.
(**A**) Corresponds to Fig. 6A for the NNC'-4 and NNC'-8 (circuits with $K = 4$ and $K = 8$ LNs). Although the activity in LNs is rather similar, it is less sparse. One can see activity in certain LNs when there was no activity in the NNC. This is because the LNs do not inhibit each other anymore.
(**B-C**) Corresponds to Figs. 6C and D for the LC'-8 and NNC'-8 (circuits with $K = 8$ LNs). The first 8 PCA variances in LC' in (**A**) do not monotonically decrease as in LC. The variances of the PCA directions are smaller, demonstrating a stronger inhibition. The spread of PCA variances is decreased in a similar way as for LC and NNC, showing that LC' and NNC' also perform a partial whitening.
(**D-E**) Corresponds to Fig. S12 for LC'-8 and NNC'-8. There is an increased mixture between the PCA directions of ORN somas ($\{\mathbf{x}^{(t)}\}_{\text{data}}$) and axons ($\{\mathbf{y}^{(t)}\}$), in comparison with the LC and NNC models. This means that the cloud of representations is not anymore compressed along the PCA directions of the input but along other linear combinations of PCA directions, which mixes the PCA directions.
(**F**) Correlation between the ORN soma $\{\mathbf{x}^{(t)}\}_{\text{data}}$ and ORN axon $\{\mathbf{y}^{(t)}\}$ for LC-8 and LC'-8. In LC-8, the axons of each ORN is more strongly correlated to its own soma for the LC-8 than for the LC'-8. This means that the neural representation at ORN axons is closer to one in ORN somas for the LC than in NNC.
(**G**) Same as (**E**) for NNC-8 and NNC'-8. Similar observations as for NNC' as for the LC'.

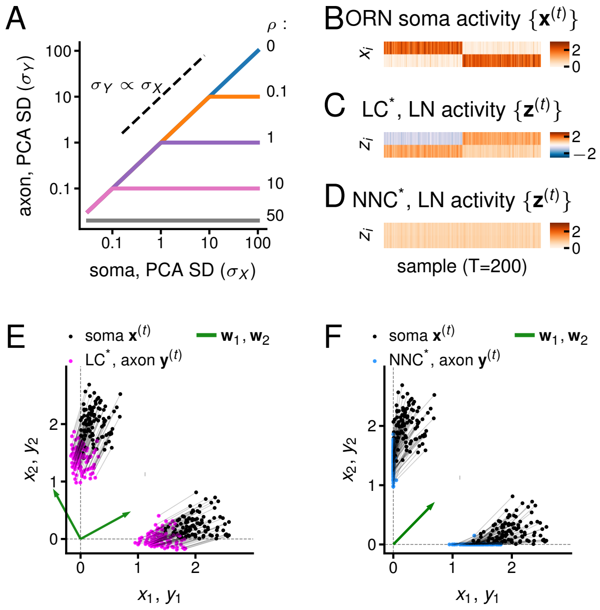

Fig. S18. LNs in circuit models without LN-LN connections.

We call LC* and NNC* the circuit models with similar architecture as the LC and NNC models, but missing the LN-LN connections from the start. This circuit corresponds to a different optimization problem (*SI Appendix*). This figure displays a similar analysis to Fig. 5.

(**A**) Transformation of the SD (σ_X, σ_Y) of PCA directions from ORN somas ($\{\mathbf{x}^{(t)}\}$) to ORN axons ($\{\mathbf{y}^{(t)}\}$) in the LC* model on a logarithmic axis, for different values of ρ, which is related to the strength of inhibition. Different line colors represent different values of ρ. For input SD smaller than $1/\rho$, the output SD remain the same. For input SD larger than $1/\rho$, the output SD becomes $1/\rho$. When $\rho = 0$, the output equals the input.

(**B**) Artificial dataset of odor representations in $D = 2$ ORN somas. The dataset was generated with two Gaussian clusters of 100 points each centered at (2, 0.) and (0., 2) with SD $= 0.3$, taking the absolute value of each coordinate. Each row is the activity in one ORN soma, each column is the representation by ORNs of one odor. This dataset is fed to the LC*-2 model (i.e., $K = 2$ LNs) (**C, E**) and the NNC*-2 model (**D, F**), $\rho = 1$.

(**C**) Each row is the activity of one LN in the LC*-2. The LNs encode the activity of the ORNs. Because there is a manifold of solutions for the LC*, LN activity can be any rotation of the activity depicted here, i.e., $\mathbf{Q} \cdot \mathbf{z}$, where $\mathbf{Q}$ is a rotation (orthogonal) matrix.

(**D**) Each row is the activity of one LN in the NNC*-2. As one can see, the activity of the LN is virtually the same, meaning in this circuit, the LNs do not perform clustering, and all are encoding the same signal.

(**E**) Scatter plot of the odor representation of dataset from (**B**) ($\{\mathbf{x}^{(t)}\}$, black) and the output at the level of ORN axons for the LC*-2 (magenta). Depicted the directions of the ORNs $\to$ LN synaptic weight vectors ($\mathbf{w}_k$) that correspond to the output in (**C**). A rotation of the LN output $\{\mathbf{z}^{(t)}\}$ would change the $\mathbf{w}_k$, but not the ORN axons output $\{\mathbf{y}^{(t)}\}$.

(**F**) Scatter plot of the odor representation of dataset from (**B**) ($\{\mathbf{x}^{(t)}\}$, black) and the output at the level of ORN axons for the NNC*-2 (blue). Note that the difference with LC* case is that all activities are nonnegative and the directions of both ORNs $\to$ LN synaptic weight vectors ($\mathbf{w}_k$) overlap, thus not resulting in any clustering (as observed in (**D**)).

Table S1. Abbreviations.

Abbreviation	Meaning
ORN	Olfactory Receptor Neuron
LN	inhibitory Local Neuron
PN	Projection Neuron, post-synaptic to ORNs
PCA	Principal Component Analysis
ZCA	Zero-phase PCA
SVD	Singular Value Decomposition
BT	Broad Trio, a type of LN
BD	Broad Duet, a type of LN
KS	Keystone, a type of LN
P0	Picky 0, a type of LN
LC	Linear Circuit, a circuit model arising from the optimization problem Eq. (4)
LC-K	LC with K LNs
NNC	NonNegative Circuit, a circuit model arising from the optimization problem Eq. (4) with the activity in ORN axons and LNs constrained to be nonnegative
NNC-K	NNC with K LNs
SNMF	Symmetric Nonnegative Matrix Factorization; e.g., (6, 7)
RCF	Relative Cumulative Frequency function
FDR	False Discovery Rate (9)
CV	Coefficient of Variation: SD/mean

Table S2. Mathematical symbols and variables.

Symbol / Variable	Meaning	Values
$\top$	matrix transpose	
$\mathrm{Tr}[\cdot]$	matrix Trace: sum of the diagonal elements	
$\mathbf{E}[\cdot]$	Expectation value	
$RCF_c(x)$	$= \frac{1}{T}\sum_{i=1}^{T} \mathbf{1}_{[-1,x]}(c_i)$: relative cumulative frequency function of a set of correlation coefficients	$0 \leq RFC_c(x) \leq 1$
$\mathbf{1}_A(y)$	indicator function of a given set A: $\mathbf{1}_A(y) = 1$ if $y \in A$, and $\mathbf{1}_A(y) = 0$ otherwise	0 or 1
r	Pearson's correlation coefficient	$-1 \leq r \leq 1$
r_+	$= \max[0, r]$ - rectified correlation coefficient	$0 \leq r \leq 1$
$\overline{r}_+$	average r_+ - mean rectified correlation coefficient	$0 \leq r \leq 1$
D	number of ORNs	21
K	number of LNs in different circuit models	from 1 to 8
$\mathbf{w}_{\text{LN}}$	D dimensional column vector, containing the number of synapses in parallel (synaptic counts) between each of the D ORNs and a specific single LN	as in Berck et al., 2016 (1), see Fig. 1B
$\mathbf{w}_{\text{LNtype}}$	$= \frac{1}{n}\sum_{\text{LN}\in\text{LNtype}} \mathbf{w}_{\text{LN}}$, D dimensional column vector, each entry is the average synaptic count from an ORN onto a given LN type LNtype (which contains n members); for Broad Trio - $n = 6$, Broad Duel - $n = 4$, Keystone - $n = 4$, Picky 0 - $n = 2$.	calculated from Berck et al., 2016 (1), see Fig. 1B
$\mathbf{x}^{(t)}$	D dimensional column vector, representing the activity of ORN soma	arbitrary
$\{\mathbf{x}^{(t)}\}$	a set of T $\mathbf{x}^{(t)}$, can refer to any (abstract) dataset	arbitrary
$\{\mathbf{x}^{(t)}\}_{\text{data}}$	set of the 170 $\mathbf{x}^{(t)}$ taken from the measurements (2), as the maximum Ca^{2+}fluorescence activation (2)	Si et al., 2019 (2)
$\mathbf{y}^{(t)}$	D dimensional column vector, representing the activity of ORN axons	
$\{\mathbf{y}^{(t)}\}$	a set of T $\mathbf{y}^{(t)}$	
$\mathbf{z}^{(t)}$	K dimensional column vector, representing the activity of LNs	
$\{\mathbf{z}^{(t)}\}$	a set of T $\mathbf{z}^{(t)}$	
Γ	measure of alignment between 2 subspaces A and B	$0 \leq \Gamma \leq \min[\dim(A), \dim(B)]$
p or pv	p-value	$0 \leq p \leq 1$
T	number of inputs/samples $\mathbf{x}^{(t)}$	170 for the Si et al., 2019 dataset (2), otherwise arbitrary
$\mathbf{w}_k$	D dimensional column vector, containing the synaptic weights between each of the D ORNs and a specific single LN. Note that in the model, the feedforward connection weight vectors are $\rho^2\mathbf{w}_k$ and the feedback connection weight vectors are $\mathbf{w}_k$	usually arising from the model
$\mathbf{W}$	$= [\mathbf{w}_1, ..., \mathbf{w}_K]$, $D \times K$ matrix containing the (feedforward) synaptic counts or synaptic weights between ORNs and LNs	either from Berck et al., 2016 (1) or from model simulations
$\mathbf{M}$	$= \{m_{i,j}\}_{i,j=1...K}$, $K \times K$ matrix containing the synaptic counts or synaptic weights between LNs; $m_{i,i}$ relates to the leak term of LN i	either from Berck et al., 2016 (1) or from model simulations
ρ	parameter of the circuit model, that encodes the strength of the feedback inhibition relative to the feedforward excitation	in the simulations $0.1 \leq \rho \leq 10$
γ	parameter of the circuit model, that only scales the activity in LNs and the synaptic weights, without affecting the nature of the computation	$\gamma = 1$ in the paper
u	unit with the physical dimension as $\mathbf{X}$, $\mathbf{Y}$, and $\mathbf{Z}$	e.g., spikes $\cdot\, s^{-1}$
$\mathbf{X}$	$= [\mathbf{x}^{(1)}, ..., \mathbf{x}^{(T)}]$, $D \times T$ matrix of $\mathbf{x}^{(t)}$	depends on the $\{\mathbf{x}^{(t)}\}$ considered
$\mathbf{Y}$	$= [\mathbf{y}^{(1)}, ..., \mathbf{y}^{(T)}]$, $D \times T$ matrix of $\mathbf{y}^{(t)}$	
$\mathbf{Z}$	$= [\mathbf{z}^{(1)}, ..., \mathbf{z}^{(T)}]$, $D \times T$ matrix of $\mathbf{z}^{(t)}$	
$\mathbf{A}^*$	the optimal $\mathbf{A}$ for the optimization problem, $\mathbf{A}$ can stand for $\mathbf{Y}$, $\mathbf{Z}$, $\mathbf{W}$, $\mathbf{M}$, etc. The * is often dropped in the text to simplify the notation when it is clear that one is talking about the optimal solution and not the variable. It is dropped in the results of the main text.	
$\{\mathbf{u}_i\}_{i=1...D}$	D PCA directions of the uncentered dataset $\{\mathbf{x}^{(t)}\}$, corresponds to the left singular vectors of the matrix $\mathbf{X}$	depends on the $\{\mathbf{x}^{(t)}\}$ considered
$\{\sigma^2_{X,i}\}_{i=1...D}$	D PCA variances of the uncentered dataset $\{\mathbf{x}^{(t)}\}$, $\{\sigma_{X,i}\}_{i=1...D}$ correspond to the square of the singular values of the matrix $\mathbf{X}$	depends on the $\{\mathbf{x}^{(t)}\}$ considered
$\{\sigma^2_{Y,i}\}_{i=1...D}$	D PCA variances of the uncentered dataset $\{\mathbf{y}^{(t)}\}$, $\{\sigma_{Y,i}\}_{i=1...D}$ correspond to the square of the singular values of the matrix $\mathbf{Y}$	

References

1. ME Berck, et al., The wiring diagram of a glomerular olfactory system. *eLife* **5**, e14859 (2016).
2. G Si, et al., Structured odorant response patterns across a complete olfactory receptor neuron population. *Neuron* **101**, 950–962.e7 (2019).
3. P Virtanen, et al., SciPy 1.0: fundamental algorithms for scientific computing in Python. *Nat. Methods* **17**, 261–272 (2020).
4. C Pehlevan, T Hu, DB Chklovskii, A Hebbian/Anti-Hebbian Neural Network for Linear Subspace Learning: A Derivation from Multidimensional Scaling of Streaming Data. *Neural computation* **1872**, 1–35 (2015).
5. C Pehlevan, A Sengupta, DB Chklovskii, Why do similarity matching objectives lead to Hebbian/anti-Hebbian networks? *Neural computation* **30**, 84–124 (2018).
6. C Pehlevan, DB Chklovskii, A Hebbian/Anti-Hebbian network derived from online non-negative matrix factorization can cluster and discover sparse features. *Conf. Rec. - Asilomar Conf. on Signals, Syst. Comput.* **2015-April**, 769–775 (2015).
7. D Kuang, H Park, C Ding, Symmetric nonnegative matrix factorization for graph clustering. *Int. Conf. on Data Min.* pp. 494–505 (2012).
8. D Lipshutz, C Pehlevan, DB Chklovskii, Interneurons accelerate learning dynamics in recurrent neural networks for statistical adaptation (2022).
9. Y Benjamini, Y Hochberg, Controlling the false discovery rate: a practical and powerful approach to multiple testing. *J. Royal Stat. Soc. Ser. B (Methodological)* **57**, 289–300 (1995).
